# Power to the people? Food democracy initiatives’ contributions to democratic goods

**DOI:** 10.1007/s10460-022-10322-5

**Published:** 2022-07-06

**Authors:** Jeroen J. L. Candel

**Affiliations:** Public Administration and Policy Group, Wageningen University, Hollandseweg 1, 6706KN Wageningen, The Netherlands

**Keywords:** Food democracy, Food policy, Democratic innovation, Food system, Food policy councils, Transparency

## Abstract

In order to foster a transition of the food system toward more sustainable outcomes, scholars have increasingly pointed at the need for organizing strengthened food democracy. By increasing the participation of citizens and food system actors, democratic innovations, such as food policy councils, are believed to promote the quality and legitimacy of food policymaking. However, the question of whether and how food democracy initiatives do indeed contribute to more democratic modes of governance largely remains unexplored. This study addresses this gap by performing a systematic literature review of the existing scholarship on food democracy, assessing democratic innovations for their contributions to four democratic goods: inclusiveness, popular control, considered judgment and transparency. The analysis shows that food democracy initiatives tend to be dominated by organized interests, have more influence on agenda-setting and implementation compared to decision-making, and generally aim for some form of deliberation or knowledge exchange. The precise selection mechanisms, processes and quality of deliberation, and transparency of democratic innovations remain important research gaps. The paper ends with a plea to better connect food democracy scholarship with the broader political sciences, as well as various suggestions for future research.

**Supplementary Information:**

The online version contains supplementary material available at 10.1007/s10460-022-10322-5.

## Introduction

The global food system is under great pressure for reform. Current ways of producing and consuming food drive some of the most pressing challenges society faces. Globally, the food system contributes around a third of anthropogenic greenhouse gas emissions (Crippa et al. 2021) and is a key driver of biodiversity loss and land-use change (Springmann et al. 2018), the depletion of groundwater resources (Wada et al. 2010) and pollution (Diaz and Rosenberg 2008). Unhealthy diets have resulted in a rapid increase of non-communicable diseases (Branca et al. 2019), endangering citizens’ welfare, as once more corroborated during the COVID-19 pandemic (Butler and Barrientos 2020), and putting a strain on public budgets (Candari et al. 2017). Additionally, many people living below the poverty line still lack access to sufficient and nutritious food (FAO et al. 2020). Meanwhile, labor conditions in the food chain are distressing, as illustrated by frequent farmer protests across the globe and ongoing concerns about the food system’s reliance on cheap and illegal labor (ILO 2020). It is for these reasons that governments, academics, civil society movements and numerous business leaders have called for a transition of the global food system toward more sustainable outcomes.

Arguably, the quest for more sustainable food systems is first and foremost a political challenge: realizing a transition of the food system requires effective governance mechanisms across policy sectors and levels, which to date remain lacking in most contexts (Barling et al. 2002; Candel and Pereira 2017). Moreover, success will be conditional on the willingness and ability of millions, if not billions, of people, from farmers and fishermen to consumers, to adjust everyday practices (Spaargaren et al. 2012). So far, the views and experiences of food system actors have hardly been incorporated into formal decision-making processes. Food policy scholars have been highly critical of the concentration of power in the hands of well-organized private interests and policy elites (McKeon 2015; Clapp 2021).

For these reasons, civil society movements, scholars and (some) governments have increasingly questioned how to better involve citizens and food system actors in food policymaking, in order to develop more effective and legitimate interventions. Both the development and study of new forms of participation and (co-)decision-making have come to be labelled as the quest for *food democracy*, referring to the degree of control that individuals and communities have over the functioning of local, national or transnational food systems. Whereas food democracy scholars have identified a broad range of innovative democratic practices emerging within civil society and food value chains (e.g., Hassanein 2003; Renting et al. 2012), this study is primarily interested in those food democracy initiatives that qualify as ‘democratic innovations’, i.e. innovative arrangements designed to mitigate democratic deficits of the policy process in traditional democratic institutions. Examples of such initiatives in the realm of food include the development of urban and regional food policy councils (Schiff 2008; Rocha 2009), the International Monsanto Tribunal (Busscher et al. 2020), and the use of deliberative citizens’ summits at national level.

Despite the high hopes surrounding these and other food democracy initiatives, they have remained ad-hoc and fragmented, raising doubts about their lasting impacts on the quality of democratic decision-making. Relatedly, while recent years have witnessed a burgeoning literature on food democracy, these studies have remained largely disconnected from larger debates on democratic innovation within political science. As a result, the evidence base about how food democracy initiatives have affected democratic goods remains absent. This is not to say that no valuable insights have been obtained, but to date these have not been integrated into a systematic and comparative research agenda. This study aims to address this gap by performing a systematic literature review that synthesizes existing studies of food democracy initiatives through a ‘democratic goods’ lens. The central question the study aims to answer is: *to what extent and how have democratic innovations within the realm of food contributed to democratic goods?* The democratic goods on which the analysis focuses are: (i) inclusiveness, (ii) popular control, (iii) considered judgment and (iv) transparency (Smith 2009). Obtaining a better understanding of these contributions is vital for both scientific and societal debates on the (de)merits of food democracy innovations.

The paper proceeds with setting out the conceptual approach, further elaborating the food democracy and democratic innovation concepts. Subsequently, the systematic literature review methods used are discussed. The fourth sectoin presents the synthesis, providing insights into the types of democratic innovations and associated outcomes emerging from the food democracy literature. The paper ends with a critical reflection on the state-of-the-art and various suggestions for future avenues of research.

## Conceptual approach

### Food democracy

Originally coined by food policy scholar Tim Lang (1999; 2005), the concept of food democracy has rapidly grown into a key avenue of debate and research within the food governance scholarship (e.g., Hassanein 2003; Hamilton 2005; Johnston et al. 2009). In their editorial accompanying a recent special issue on the topic, Bornemann and Weiland (2019a, p. 4) explain this interest by the concept’s potential to allow for “new insights into the democratic conditions and consequences of recent developments in the food system”, while, conversely, it can “shed new light on the consequences of recent democratic transformations for the governance of contemporary food systems.” Despite this growing interest, the concept remains characterized by diverging interpretations and considerable ambiguity. Behringer and Feindt (2019), in this respect, distinguish between two distinct articulations of food democracy discourse that have developed over time. A first articulation they refer to as “liberal food democracy” and has its roots in political consumerism, emphasizing the steering role consumers can adopt through their daily consumption choices (see also: Lorenzini 2019). The second articulation, labelled “strong food democracy”, departs from this market-based orientation and focuses on the emergence of citizen-led processes and initiatives through which participation and agonism are organized in alternative ways (see also the work on deep food democracy, e.g., Carlson and Chappell 2015). This perspective draws attention to the broad range of innovative food governance arrangements that have emerged in recent years, which are the prime interest of this study. One could add that despite the concept’s anti-hegemonic roots, such innovations are not exclusively initiated by citizens but may also be government-led (Griend et al. 2019). Moreover, questions of food democracy could, in principle, also relate to the functioning of traditional representative institutions themselves, although this has remained largely ignored to date (Baldy and Kruse 2019).

Apart from food democracy, various related concepts and discourses with a strong democratic dimension or connotation have emerged. The most resonating of these are ‘food sovereignty’ and ‘food justice’. The food sovereignty concept was originally developed by farmer movements in the Global South, notably La Vía Campesina, and has diffused as a counter-discourse to the dominant, and allegedly neoliberal, food security discourse. Proponents of food sovereignty call for the inclusion of small-scale producers in collective decision-making arrangements and, more generally, propel the sovereignty of local communities in shaping their food systems (McMichael 2014; Dekeyser et al. 2018). Likewise, food justice activists and scholarship draw attention to inequalities of race, class and gender in the current food system, and call for the development of alternative models and practices (Glennie and Alkon 2018). Both concepts have been used by civil society movements to legitimize novel democratic practices, because of which they are included in this study’s review (see methods section).

Building on the above, this study does not aim to provide an exhaustive account of all interpretations and manifestations of food democracy, but is primarily interested in those initiatives and arrangements in the realm of food systems that can be qualified as ‘democratic innovations’, i.e. novel ways of organizing citizen participation in the formal democratic process (see below). The most prominent example of such innovations is the emergence of ‘food policy councils’, i.e. civil society organizations which aim for food system transformation through influencing existing political processes and institutions (Schiff 2008; Prové et al. 2019). Other types of democratic innovations within the realm of food include the emergence of citizen tribunals (Busscher et al. 2020), hackatons (Termeer and Bruinsma 2016), or citizen summits at national level, such as France’s ‘National Food Conference’ (Candel et al. 2020). Whereas the academic corpus about such initiatives, food policy councils in particular, is rapidly growing, relatively few systematic and comparative assessments going beyond single-*n* thick case descriptions have been undertaken to date. In addition, while the democratic potential of these initiatives in fostering new forms of participation and more inclusive institutions have been widely acknowledged and propagated, they have hardly been connected to and approached from the broader political science scholarship on the functioning and impacts of democratic innovations. This study aims to take a first step in that direction.

### Democratic innovations and democratic goods

The quest for food democracy resonates with recent debates in political science about the broader need for democratic innovation in liberal democracies. Theorizing democratic innovation has its roots in various branches of democratic scholarship, most notably on participatory and deliberative democracy (Fung 2006; Saward 2006). Scholars in this tradition generally presume that liberal democracy is in hot water, as growing economic inequality (Schäfer 2012), increased levels of false information circulating through social media (Bennett and Livingston 2018), a worsening quality of deliberation (Gora and Wilde 2020), and a re-emergence of identity politics (Börzel and Risse 2018), have challenged the effectiveness and legitimacy of traditional democratic institutions in governing society towards desired directions (Mounk 2018). To mitigate these challenges and revive citizens’ commitment to and participation in the democratic process, governments, civil society movements and academics have initiated and tested a wide variety of democratic innovations in recent decades. The commonality of these innovations is that they aim for improved democratic processes and outcomes through increasing and deepening citizen participation in political decision-making (Smith 2009). Moreover, many of these innovations, particularly also in the realm of food (Moragues-Faus 2017), aim to go beyond post-political practices of managerialism and technocracy by providing new spaces for dissensus, particularly for those who previously lacked a voice in political decision-making (O’Flynn 2019). As such, democratic innovations, when carefully designed and operating in synergy with representative institutions (Fung 2006), are expected to play an important role in addressing the pressing challenges that liberal democracies face.

This paper’s review of food democracy initiatives focuses on both the *type* and *outcome* of democratic innovations. Regarding the former, democratic innovations come in different types, ranging from referenda and mini-publics to participatory budgeting and e-participation tools (OECD 2020). Different types of democratic innovations also differ in the schools of thought that underlie them, resulting in variation in whether they, for example, favor direct democracy or the strengthening of existing representative models, or whether they are rooted in deliberative or more agonistic approaches. A commonality is that most initiatives are concerned with strengthening *participatory* democratic innovation, while other types of, non-participatory, improvements to representative institutions have largely been ignored within this literature (Smith 2019). Additionally, a key defining characteristic is that these democratic innovations are intended to mitigate shortcomings of traditional democratic institutions and therefore, in a direct or looser way, engage with formal policy processes. Consequently, self-organizing, market-based, or other types of innovative democratic practices without such an explicit link to the policy process fall outside of the scope of this study.

There can also be considerable variation *within* the same type of democratic innovation. There has, for example, been a vivid academic debate on what constitutes a ‘mini-public’, which are commonly understood as ‘institutions in which a diverse body of citizens.. reason together about an issue of public concern’ (Smith and Setälä 2018). Whereas some scholars have argued that a mini-public is defined by a random selection of participants and the presence of neutral process facilitators, others allow for more variation in designs (Ryan and Smith 2014).

Regarding the outcomes of democratic innovations, a common approach is to study the extent to which they contribute to so-called ‘democratic goods’. The most common conceptualization of these democratic goods is provided by Smith (2009), who distinguishes between inclusiveness, popular control, considered judgment and transparency, which are summarized in Table 1. These criteria cover both aspects of input and output legitimacy, i.e. procedural fairness and the ability to produce effective outcomes (Scharpf 1997).

**Table 1:** Overview of democratic goods, adopted from Smith (2009)

Democratic good	Explanation
Inclusiveness	The ability of citizens from across different social groups to evenly participate in political decision-making. Includes both formal characteristics of selection mechanisms and the extent to which in practice institutional inducements motivate the engagement of citizens from across groups, so as to avoid marginalization or exclusion
Popular control	The degree in which participants are afforded increased influence and control within the decision-making process, covering problem definition, option analysis, option selection and implementation
Considered judgment	The capacity of citizens to make thoughtful and reflective judgments, including understanding of both the technical details of the issue under consideration and the perspective of other citizens
Transparency	The openness of proceedings to both participants and the wider public

Whereas the state-of-the-art on democratic innovations show promising findings for individual cases in contributions to these four democratic goods, most democratic innovations have not yet lived up to their potential in terms of mitigating the systemic challenges to democracy. So far, initiatives have largely been applied randomly and ad-hoc, with little integration into existing representative institutions and processes (Geissel 2019). This fragmentation is reflected in research on the topic, as many studies have remained limited to one or few exemplary cases, with little attention to more mundane forms of enhancing democracy (Smith 2019). Moreover, scholars have pointed out that, despite their post-political ambitions, many democratic innovations in practice tend to *de*politicize conflict (Meriluoto 2021), as such having system-reinforcing rather than transformative effects (Goetz et al. 2020).These observations have resulted in calls for more systemic designs, taking the interconnections between sub-entities and governance levels into account (Owen and Smith 2015), as well as more comparative research designs (Ryan 2019). The present study contributes to the latter by providing a first comparative assessment of democratic innovations within the realm of food policy.

## Methodological approach

To examine current insights into food democracy initiatives’ contributions to democratic goods, a systematic review of the state-of-the-art was performed. Compared to traditional reviews, systematic review methods reduce researcher bias in the identification, selection and analysis of relevant publications, allowing for greater transparency and reproducibility of the research steps taken (Petticrew and Roberts 2006). A broad search query, consisting of: (i) the term food democracy, or (ii) specific types of food democracy initiatives such as food policy councils, citizen tribunals, participatory and collaborative governance arrangements, or (iii) the related concepts of food sovereignty and food justice mentioned within a distance of 20 words from democracy, was applied (Online resource 1). An initial search of titles, abstracts and keywords in Scopus, which is the most comprehensive database of scientific journal publications, in May 2021 resulted in 193 results. Applying the inclusion and exclusion criteria (Online resource 1) to the abstracts, titles and keywords resulted in a preliminary selection of 71 publications. Subsequent application of the criteria to the full articles resulted in a final database of 33 publications. Importantly, only studies published in English were included, possibly resulting in a geographical bias. No methodological quality assessment of these publications was performed.

All articles were read and relevant passages were extracted into a data extraction matrix (Online resource 2). The data extraction matrix for each publication provides: (i) the geographical locus and period of the democratic innovation(s) described, (ii) the type of democratic innovation, (iii) the conceptual and methodological approach of the paper, and (iv) any relevant insights into initiatives’ impacts on the four democratic goods of inclusiveness, popular control, considered judgment and transparency.

Subsequently, the data extraction matrix was coded using a codebook and the coding software *Atlas.ti*. Codes were developed deductively, based on Smith’s (2009) volume on democratic innovation, complemented with Camilla Adelle’s (2019) research on the role of knowledge in food democracy to further specify the various types of knowledge under the dimension of considered judgment. Additionally, inductive codes that emerged from the data were added, particularly regarding the different types of actors involved. Table 2 provides an overview of the codes used (see Online resource 3 for the full codebook). After coding, all quotations were compared for common patterns and (dis)similarities, resulting in the synthesis presented in the results section.

**Table 2:** Code categories used per democratic good

Inclusiveness	1. Groups that were (largely) included or excluded; separate codes for specific groups 2. Selection mechanisms; separate codes for specific selection mechanisms 3. Presence of institutional inducements to engage citizens from across groups 4. General reflections about inclusiveness
Popular control	1. Agenda-setting 2. Policy formulation 3. Decision-making 4. Implementation 5. Evaluation For each of codes 1–5 specific sub-codes for large influence/ control, some influence/ control and no or hardly any influence/ control 6. General reflections about popular control
Considered judgment	1. Types of knowledge and information considered; separate codes for specific types of knowledge 2. Process of deliberation; separate codes for presence or absence of various deliberative good practices 3. General reflections about considered judgment
Transparency	1. Efforts taken to open proceedings to participants (or lack thereof) 2. Efforts taken to open proceedings to wider public (or lack thereof) 3. General reflections about transparency

## Results

### Description of the dataset

Table 3 provides an overview of the food democracy initiatives covered in the review. A large majority of the studies included focus on local or regional food policy councils in North America and Europe. It is not clear whether this is due to the increasing popularity of this type of democratic innovation, or due to a bias in the food democracy scholarship. Limited attention to citizen tribunals, other types of collaborative arrangements, indigenous practices and governance initiatives in the Global South suggest that the latter plays *some* role at least.

**Table 3:** Overview of democratic innovations covered in the dataset

Author(s), (year)	Type of democratic innovation(s)	Place and period of study
Andreola et al. (2021)	Food policy council	Trento, Italy; 2019–2020
Baldy and Kruse (2019)	Civil dialogues and expert dialogue	Two smaller cities in Southern Germany (anonymised); years unknown
Bassarab et al. (2019)	Food policy councils	United States; 2018
Blay-Palmer (2009)	Food policy council	Toronto, Canada; 1990–2009
Boossabong (2017)	Collaborative governance network on urban agriculture	Bangkok, Thailand; 2010–2012
Calancie et al. (2018)	Food policy councils	United States, Canada and Tribal & First Nations; 2015
Calancie et al. (2017a, b)	Food policy councils	United States, Canada and Tribal & First Nations; 2015
Calancie et al. (2017a, b)	Food policy council	Adams County, Pennsylvania, United States; 2014
Clancy et al. (2008)	Food policy councils	North America
Clark et al. (2017)	Food policy council	Franklin County, Ohio, United States; 2012–2015
Clayton et al. (2015)	Food policy councils	United States; 2011–2012
Fridman and Lenters (2013)	Food policy council	Toronto, Canada; 2011
Giambartolomei et al. (2021)	Food policy councils	Cork, Ireland & Bergamo, Italy; 2016
Godek (2021)	Community networks; SSAN (Law of Food and Nutritional Sovereignty and Security) committees: COMUSSANs	Nicaragua; 2007–2018
Hasson (2019)	Food policy council	London, United Kingdom; 2017–2019
Henson and Munsey (2014)	Food policy council	Birmingham, Alabama, United States; 2010–2011
Horst (2017)	Food policy council	Puget Sound region, Washington, United States; 2010–2015
Koski et al. (2018)	Food policy council	A Western region of the United States (anonymised); 2013–2014
Lange et al. (2021)	Food policy councils	United States; 2014
MacRae (1994)	Food policy council	Toronto, Canada; 1990–1994
Mah and Thang (2013)	Food policy council	Toronto, Canada; 2010–2011
Mangnus et al. (2019)	Food policy council (game)	Kyoto, Japan; years unknown
Mooney et al. (2014)	Food policy councils	United States; 2006–2013
Pothukuchi and Kaufman (1999)	Food policy councils	United States; years unknown
Prové et al. (2019)	Food policy councils	Ghent, Belgium & Philadelphia, Pennsylvania, United States; 2013–2015
Roberts (2010)	Food policy council	Toronto, Canada; years unknown
Sadler et al. (2015)	Food policy council (in the making)	Flint, Michigan, United States; 2011–2012
Santo and Moragues-Faus (2019)	Trans-local food policy networks	United States and United Kingdom; 2016
Schiff (2008)	Food policy councils	United States and Canada; years unknown
Siddiki et al. (2015)	Food policy councils	United States; years unknown
Sieveking (2019)	Food policy council	Oldenburg, Germany; 2016–2018
Thompson et al. (2020)	Food policy council	Oktibbeha County, Mississippi, United States; 2016–2019
Zerbian and De Luis Romero (2021)	Food policy platform	Madrid, Spain; 2019

Studies varied considerably in terms of their conceptual and methodological approaches, ranging from studies providing thick empirical case descriptions without clear conceptual focus, to studies addressing specific conceptual questions on themes such as collaborative governance, framing or the role of advocacy coalitions. The latter shows that studies of food democracy initiatives have been relatively well connected with broader theorization in political science, although hardly with democratic theory, or the scholarly debate on democratic innovation more specifically. Methodologically, most studies made use of interviews, field observations or surveys. There were no studies directly assessing food democracy initiatives’ impacts on democratic goods, e.g., through experimental set-ups or before-after assessments. Given the large amount of anecdotal and indirect evidence, the synthesis presented in this section should be considered as a first attempt at theory-building, providing a starting point for more systematic and comparative studies.

### Inclusiveness

The meta-analysis shows that there is considerable diversity in which type of actors are reported to be involved in food democracy initiatives. Actor groups that were mostly observed to be (largely) included in democratic innovations were (both urban and ‘conventional’) farmers (16 publications), government officials or politicians (16), NGOs and civil society (16) and food chain actors other than primary producers and consumers, e.g. local businesses or retailers (13). Other groups, such as citizens (8), researchers, academics and students (6), schools and educational institutes (6), healthcare and public health actors (7), and labour unions (1) were mentioned less frequently. Most likely, the involvement of some of these groups, such as citizens, is underreported, as researchers may consider their inclusion as evident and not requiring explicit reflection. The overall image that arises, however, is that those governing or working in the food system, as well as organized interests, tend to be more often involved than other types of professionals and citizens.

The latter particularly applies when looking at those groups which were explicitly referred to as being (largely) excluded. Various studies reported a lack of inclusion of citizens in general (3), citizens from low SES groups (3), or from specific ethnic backgrounds (2). In a discussion of the Baltimore Food Policy Initiative, Bassarab et al. (2019), for example, drawing on earlier work of Swartz et al. (2018, p. 33), argue that those choosing to participate in the city’s Food Policy Action Coalition “do not reflect the majority of Baltimore’s population and are generally not people directly affected by food system problems”. In a fierce critique of the food policy council in Birmingham, Alabama, Henson and Munsey (2014) state that the local food movement is “deeply racialized” due to the lack of involvement of the city’s black community, which makes up the largest population group. Borrowing from the work of Bourdieu (1977), they argue that these exclusionary practices are not purposeful, but rather “the result of the more or less automatic operation of the habitus, produced in White space, and functioning in White fields.” (p. 1015) Other groups that were found to be excluded in a number of studies were farmers (4), government officials (2) and NGOs and civil society (5). Regarding the latter, Koski et al. (2018) found that a lack of capacity and resources can be a major constraint to non-profits’ participation in a food policy council.

Membership matters, as the types of actors participating in food democracy initiatives have been found to have a large influence on the types of issues that are discussed and ultimately acted upon (Siddiki et al. 2015; Bassarab et al. 2019). Calancie et al. (2017a, b), for example, refer to a study by Hays et al. (2000) to argue that greater racial diversity is associated with community coalitions’ ability to influence public policy. At the same time, overall participation rates do not tell the fully story, as even when there is a large diversity of actors on paper, degrees of participation may vary considerably. Koski et al. (2018) in this respect speak of a “council within a council” to denote that a small group of actors’ influence within the food policy council was enhanced by their consistently high attendance at meetings. Limited time and human resources proved major constraints for citizens and civil society groups in cities such as Bergamo, Cork (Giambartolomei et al. 2021), and Oldenburg (Sieveking 2019).

In terms of selection mechanisms, relatively few studies reported how participants to food democracy initiatives got selected. In those that did, the selection mechanism that was found to be most common was the invitation of participants by governmental actors (7 studies). In a comparative study of food policy councils in North America, for example, Clancy et al. (2008) found that in most councils members were appointed by either the mayor, county or state legislatures. Similarly, participants of the London Food Board had to be appointed by the mayor (Hasson 2019). Three studies reported participants to be selected by non-governmental actors, e.g. food policy council organizers originating from civil society, and two studies indicated that there was no selection mechanism, meaning that participation was open to all who were interested. Sortition, generally considered a good practice for mini-public style democratic innovations, was used as selection mechanism in none of the food democracy initiatives under study.

Relatively few studies (6) mentioned the presence of institutional inducements to engage citizens from across diverse groups. Exceptions included two anonymous southern German cities, where citizens were actively invited through different media (Baldy and Kruse 2019), the use of supportive working groups in Austin, for which citizens were actively invited (Bassarab et al. 2019), or the use of ‘communicative fora’ that were opened to peri-urban farmers and slum communities in Bangkok to share their knowledge and concerns after being affected by flooding (Boossabong 2017).

### Popular control

The state-of-the-art shows that there is considerable variation in the degree of popular control that participants of food democracy initiatives (can) exercise over the various phases of the policy cycle. The overall image that arises is that—in their relation to public policy—food democracy initiatives play relatively large roles in agenda-setting (17 studies indicating large influence or control, 2 studies medium influence) and policy formulation (8 vs. 4), a more modest role in implementation (4 vs. 3), and hardly any role in actual decision-making and policy evaluation.

In terms of agenda-setting and policy formulation, many food democracy initiatives, notably food policy councils, have been found to play an important role in translating food-related concerns and ideas within the community to policy agendas, as various studies for example find for the much-studied food policy council of Toronto (MacRae 1994; Mah and Thang 2013). In many cases, local governments actively reach out to food policy councils to receive policy advice. In some cities, such as Philadelphia, food policy councils are even integrated within the administration to foster collaboration across sectoral departments (Prové et al. 2019). Various studies found that participants were also closely involved in policy implementation. Schiff (2008), for example, observes that government-initiated food policy councils after their first years often shift from a focus on policy development to the implementation of programs once recommendations have been made.

On the contrary, the influence on decision-making is found to be very limited. This was often due to the simple fact that in most democratic systems decision-making powers are reserved for elected politicians (e.g., Baldy and Kruse 2019). In some cases, however, this lack of involvement resulted from a disinterest from the side of government. In the extreme case of the Food and Nutrition Sovereignty Committees in Nicaragua, Godek (2021) found that the government intentionally used these committees as “vehicles for state co-optation” by filling them with people loyal to the ruling party. Also, some food policy councils deliberately choose not to focus on policy, or even outwardly oppose formal policymaking, even when they often prove to implicitly do policy-relevant and -influencing work (Schiff 2008; Mooney et al. 2014). The latter suggests that despite limited direct influence on decision-making, through their agenda-setting and policy formulation roles, food democracy initiatives *do* affect policy adoption in more indirect ways. Lange et al. (2021), in this respect found that having a food policy council is associated with the presence of (more) municipal-level policies or practices to improve access to healthy foods. For policy evaluation, hardly any participation of citizens or food system actors has been described. However, it is unclear whether this is due to limited scholarly attention to such processes, the absence of participatory processes, or limited evaluations of food policies in the first place.

Within these overarching patterns, notable differences between the (experienced) efficacy of food democracy initiatives are observed, for which various explanatory conditions are provided. First, there are notable differences in the competences that different layers of government may exercise over food system issues, whereby local governments often have limited influence over bigger questions (Bassarab et al. 2019). Second, resources and capacity are key. Siddiki et al. (2015) found that food policy councils that lacked stable funding from local or regional governments generally had fewer and narrower outputs. Third, the more general willingness on behalf of government, including elected politicians, to engage with democratic innovations and adopt their outputs into decision-making proves crucial (Bassarab et al. 2019). Negative experiences with government commitment result in stakeholder fatigue and low expectations about efficacy (Baldy and Kruse 2019).

### Considered judgment

Compared to inclusiveness- and popular control-related aspects, relatively few insights have been obtained on the extent to which interactions within food democracy initiatives contribute to the democratic good of considered judgment. To start, few studies included in the review explicitly reported on the types of knowledge and information being exchanged within democratic initiatives. The type of knowledge that is mentioned most often is experiential knowledge of food system actors and stakeholders (5 studies), such as farmers. Andreola et al. (2021), for example, show how experiential knowledge of producer representatives, small-scale producers and food activists was used in heated debates about the potential of cooperative agricultural models in Trento’s food policy council. Scientific and cultural or indigenous knowledge were both observed to be included by two studies; policy or political knowledge in none of the studies. The views and perspectives of marginalized groups, e.g. those suffering from food insecurity, were found to be included by in Bangkok’s collaborative governance network on urban agriculture (Boossabong 2017), while largely excluded in the food policy council of Birmingham, Alabama (Henson and Munsey 2014).

Similarly, few studies have reported on whether or not good practices of deliberation, which many food democracy initiatives explicitly or implicitly aim for, have been applied. Ten studies reported that, indeed, some sort of deliberation or knowledge exchange has taken place in the democratic innovations under study. For example, MacRae (1994) shows how in the early days of the Toronto Food Policy Council representatives of different sectors engaged in discussions to understand each other’s views, resulting in decisions that went beyond the lowest-common-denominator position. Two studies reported the absence of such deliberation. Both in the cases of Trento (Andreola et al. 2021) and Madrid (Zerbian and de Luis Romero 2021), interactions between stakeholders were found to lack depth and genuine exchange. Only two studies mentioned the presence of neutral process facilitators (Fridman and Lenters 2013; Calancie, Stritzinger, et al. 2017a, b). Findings on whether participants of food democracy initiatives have equal opportunities to engage in deliberations or share their perspectives are mixed (3 studies indicating they do, 4 they don’t). The same applies to participants’ willingness to reflect on their own and others’ frames and come to a shared understanding of problems and/or solutions (3 versus 3). Whether or not participants engage in deliberations without instruction from or consultation with any principals was reflected upon in only three studies (2 studies yes, 1 no). Siddiki et al. (2015, p. 544), for example, found that the involvement of government officials in North American food policy councils can slow down decision-making, as these “served as proxies for agency heads”, having “to take all issues back to their administrators for approval or disapproval”. Only two studies reported on the actual language being used in the interactions within the initiatives under study (Henson and Munsey 2014; Baldy and Kruse 2019). Both these studies found the language to be insufficiently inclusive, as such not allowing for equal participation. Henson and Munsey (2014, p. 1013), for example, state that in Birmingham’s food policy council, “[t]he language used was technical and focused on how to package food movement ideology—the importance of local food and farmers, the benefits for health (specifically childhood obesity)—in ways that would be palatable to middle- and upper-class Whites.”

### Transparency

Of the four democratic goods, transparency proved the one least reported on in the reviewed studies of food democracy initiatives. Only one study included observations about transparency toward initiatives’ participants, i.e. the efforts taken to open proceedings. In her study of the Oldenburg food policy council, Sieveking (2019) finds mixed evidence for these efforts. On the one hand, she observes that minutes of meetings were always being taken, so that people were able to follow what had been discussed. On the other, she states that “as time went on, it became increasingly difficult for the members to monitor their activities” (p. 53). As work committees did not report consistently, proceedings became less clear to newcomers.

Regarding transparency toward wider publics of non-participating actors, five studies observe efforts taken to open food democracy initiatives’ proceedings. In his comparative study of food policy councils in North America, Schiff (2008), for example, found that councils communicate their ideas and information through a number of outlets, including information booths, events, published and online materials. In two studies, a lack of such transparency was found. Clancy et al. (2008), for example, describe that whereas some North American food policy councils invest considerably in their exposure, others deliberately opt for a low public profile, as they believe a behind the scenes way of working is more effective in influencing policy. For similar reasons, many food policy councils evade media exposure (Schiff 2008). In the case of two anonymous Southern German cities, Baldy and Kruse (2019) observed that information about the selection processes through which participants to expert dialogues were invited was not transparent.

On the overall importance of transparency for the success of food democracy initiatives, Baldy and Kruse (2019) reflect that an openness of exchanges toward wider audiences is an important prerequisite for true deliberation and dialogue. At the same time, efforts to open up proceedings on behalf of food democracy initiatives might in themselves not be sufficient, as little awareness among (potential) beneficiaries may make that these efforts do not find fertile ground (Zerbian and de Luis Romero 2021).

## Conclusions and discussion

This study started from the objective of synthesizing the state-of-the-art on the extent to which democratic innovations aimed at mitigating the democratic deficits of traditional democratic institutions in the realm of food have proven to contribute to the democratic goods of inclusiveness, popular control, considered judgment and transparency. The meta-analysis presented in the previous section shows that scholarship on these type of food democratic innovations (in contrast to political consumerism) is limited yet nascent. In this respect, the food democracy scholarship mirrors the practice of food governance, as many governments have only recently started to explore notions of food democracy and related concepts (Smaal et al. 2021). This also explains why scholars so far have mainly concentrated on food policy councils, which—at least in North America and Europe—have been the most common type of democratic innovation in food-related policy processes. It would be interesting to complement and compare these insights with other types of emerging democratic innovations, such as citizen summits and citizen tribunals.

Despite the small corpus of evidence, some cautious conclusions regarding food democracy initiatives’ contributions to the four democratic goods can already be drawn, especially for inclusiveness and popular control, which have received most attention so far. For *inclusiveness*, participation in food democracy initiatives proves to be highest among food system professionals, such as farmers, governmental actors and non-profit organizations. Whereas there seems to be some underreporting of the participation of citizens, there is ground for concern about the limited involvement of citizens from marginalized groups, e.g., with low socio-economic status or with specific ethnic backgrounds. However, participation rates do not tell the full story, as the scope and depth of participation differs along the resources that groups have available, again favouring better organized interests. The selection mechanisms that are used remain a bit of a black box; participation by governmental invitation being observed most often. Similarly, the presence of institutional inducements to engage citizens from across diverse groups has hardly been reflected upon.

Contributions to *popular control* are clearest for the phases of agenda-setting, policy formulation and, to a lesser extent, implementation. Participants of food democracy initiatives prove to have much less influence on ultimate decision-making and evaluation. These findings suggest that food democracy initiatives have primarily contributed to translating societal views and ideas into policy processes, as well executing specific programs. It should be noted, however, that considerable differences exist between initiatives, partly explained by the competences, resources and political buy-in available. The precise mechanisms through which these factors and broader political contexts shape democratic outcomes would be an important future avenue of research (cf. Biesbroek and Candel 2019).

Levels and processes of *considered judgment* proved to have attracted relatively little scholarly attention to date. Many food democracy initiatives do indeed seem to involve some sort of deliberation and/or exchange of knowledge, but the precise interactions, types of knowledge included, use of deliberative good practices, and use of language all remain virgin territory. The same applies to the democratic good of *transparency*: so far, the efforts that food democracy initiatives’ organizers have taken to open up proceedings toward both participants and broader publics remain a blind spot. Future comparative studies will have to establish to what extent such efforts exist.

The range of gaps this study identified shows that much additional research is needed to arrive at a more comprehensive understanding of the conditions under which food democracy initiatives (may) contribute to democratic goods. In unrolling such a research agenda, it is imperative to (better) connect food democracy scholarship with broader political science, drawing on and contributing to democratic innovation theory, as well as making use of recent advancements in methodological approaches (Elstub and Escobar 2019). This body of scholarship also provides a plethora of design principles, which food democracy researchers can use when advising governments or designing democratic initiatives or experiments of their own.

Following from the above, it is still too early to tell how food democracy initiatives compare to democratic innovations in other domains, such as climate change or medical-ethical issues. To date, there has been little comparative research of similar type of democratic innovations across different domains, which would allow for assessing whether the topic of deliberation plays a role in the first place. In theory, food-related issues may have a considerable mobilizing potential among citizens (cf. Bornemann and Weiland 2019b), making food system governance a promising experimentation ground for broader democratic innovation. As such, food democracy initiatives may provide lessons and insights that could help to mitigate the larger crisis liberal democracies find themselves in.

A related question deals with the expediency of investing in food democracy initiatives in non-democratic societies. On the one hand, it could be argued that such initiatives could grow into seeds of broader transformative change (cf. Bennett et al. 2016). At the same time, however, Godek’s (2021) study of Food and Nutrition Sovereignty Committees in Nicaragua shows that democratic innovations can be powerful tools for state co-optation and result in the further marginalization of vulnerable groups. As the current evidence base of food democracy initiatives in non-democratic societies is very thin, future research will have to show whether and how the latter mechanisms can be prevented.

Importantly, food democracy initiatives are not the Holy Grail for addressing the democratic deficit in global food systems. The democratic innovation literature has shown that while individual initiatives may yield promising outcomes, repairing the wider deficit requires a systemic turn, improving the democratic quality of political systems at large. Such a turn would involve connecting democratic innovations across levels and contexts (Owen and Smith 2015), as well as complementing and connecting these participatory arrangements with democratic innovations *in* traditional representative institutions (Smith 2019). What such a systemic turn would entail for food systems governance should be a central question in a future research agenda.

More generally, it remains an open question to what extent, and under what conditions, democratic innovations actually manage to move beyond the post-political dynamics of technocracy and managerialism they seek to overcome. Moreover, it would be worth further exploring and debating the very foundations that underly most of the democratic innovation movement, as some argue its liberalist assumptions may actually be at the root of many societal woes. Connecting democratic innovation scholarship with alternative schools of political philosophy, such as communitarianism or environmentalism, could result in a larger variety of problem analyses and democratic experiments, possibly enlarging their potential to mitigate food system challenges. In this respect, it would be worth adding a fifth dimension to Smith’s typology, focusing on the basal question of whether participants think ‘democratic innovation’ as such lies at the heart of their concerns, what their concerns are, and how they see those concerns relating to the societal woes identified. Broadening the current study’s focus to a wider array of innovative democratic practices in the food system, such as community-supported agriculture or buying groups, could be a fertile entry point for such exploration.

Despite the many open questions, governments and other organizers of food democracy initiatives can already draw lessons from the meta-analysis presented in this study. More careful consideration of selection mechanisms, investing in efforts to involve marginalized groups, ensuring sufficient resources and political buy-in, adopting good practices of deliberation, and enhancing transparency, are all ways of increasing the impact of existing and future initiatives. Many types of democratic innovation, such as participatory budgeting, e-participation and genuine mini-publics, have hardly been used in the food domain and would merit further experimentation. Given the urgency of the food system crisis and the need for rapid behavioural change, there is little time to loose in fostering more effective and legitimate governance arrangements.

## Supplementary Information

Below is the link to the electronic supplementary material.

Supplementary file1 (DOCX 12 kb)

Supplementary file2 (DOCX 164 kb)

Supplementary file3 (DOCX 13 kb)

## Abbreviations

NGOs: Nongovernmental Organizations
SES: Socioeconomic status

## References

[CR1] Adelle C (2019). The role of knowledge in food democracy. Politics and Governance.

[CR2] Andreola M, Pianegonda A, Favargiotti S, Forno F (2021). Urban food strategy in the making: Context, conventions and contestations. Agriculture (switzerland).

[CR3] Baldy J, Kruse S (2019). Food democracy from the top down? State-driven participation processes for local food system transformations towards sustainability. Politics and Governance.

[CR4] Barling D, Lang T, Caraher M (2002). Joined–up food policy? The trials of governance, public policy and the food system. Social Policy & Administration.

[CR5] Bassarab K, Clark JK, Santo R, Palmer A (2019). Finding our way to food democracy: Lessons from US food policy council governance. Politics and Governance.

[CR6] Behringer J, Feindt PH (2019). How shall we judge agri-food governance? Legitimacy constructions in food democracy and co-regulation discourses. Politics and Governance.

[CR7] Bennett EM, Solan M, Biggs R, McPhearson T, Norström AV, Olsson P, Pereira L (2016). Bright spots: Seeds of a good Anthropocene. Frontiers in Ecology and the Environment.

[CR8] Bennett WL, Livingston S (2018). The disinformation order: Disruptive communication and the decline of democratic institutions. European Journal of Communication.

[CR9] Biesbroek R, Candel JJL (2019). Mechanisms for policy (dis)integration: Explaining food policy and climate change adaptation policy in the Netherlands. Policy Sciences.

[CR10] Blay-Palmer A (2009). The Canadian pioneer: The genesis of urban food policy in Toronto. International Planning Studies.

[CR11] Boossabong P, Allen A, Griffin L, Johnson C (2017). Floods and food in the city: Lessons from collaborative governance within the policy network on urban agriculture in Bangkok, Thailand. Environmental justice and urban resilience in the global south.

[CR12] Bornemann B, Weiland S (2019). Editorial: new perspectives on food democracy. Politics and Governance.

[CR13] Bornemann B, Weiland S (2019). Empowering people—Democratising the food system? Exploring the democratic potential of food-related empowerment forms. Politics and Governance.

[CR14] Börzel TA, Risse T (2018). From the euro to the Schengen crises: European integration theories, politicization, and identity politics. Journal of European Public Policy.

[CR15] Bourdieu P (1977). Outline of a theory of practice.

[CR16] Branca F, Lartey A, Oenema S, Aguayo V, Stordalen GA, Richardson R, Arvelo M, Afshin A (2019). Transforming the food system to fight non-communicable diseases. BMJ.

[CR17] Busscher N, Colombo EL, van der Ploeg L, Gabella JI, Leguizamón A (2020). Civil society challenges the global food system: The International Monsanto Tribunal. Globalizations.

[CR18] Butler MJ, Barrientos RM (2020). The impact of nutrition on COVID-19 susceptibility and long-term consequences. Brain, Behavior, and Immunity.

[CR19] Calancie L, Allen NE, Ng SW, Weiner BJ, Ward DS, Ware WB, Ammerman AS (2018). Evaluating food policy councils using structural equation modeling. American Journal of Community Psychology.

[CR20] Calancie, L., N.E. Allen, B.J. Weiner, S.W. Ng, D.S. Ward, and A. Ammerman. 2017a. Food policy council self-assessment tool: Development, testing, and results. *Preventing Chronic Disease* 14(3). 10.5888/pcd14.160281 PMC5338598 28253474

[CR21] Calancie L, Stritzinger N, Konich J, Horton C, Allen NE, Ng SW, Weiner BJ, Ammerman AS (2017). Food policy council case study describing cross-sector collaboration for food system change in a rural setting. Progress in Community Health Partnerships: Research, Education, and Action.

[CR22] Candari, C.J., J. Cylus, and E. Nolte. 2017. *Assessing the economic costs of unhealthy diets and low physical activity: An evidence review and proposed framework*. 47. Health Policy Series. Geneva: World Health Organization. 28787114

[CR23] Candel JJL, Parsons K, Barling D, Loudiyi S (2020). The relationship between Europeanisation and policy styles: A study of agricultural and public health policymaking in three EU Member States. Journal of European Public Policy.

[CR24] Candel JJL, Pereira L (2017). Towards integrated food policy: Main challenges and steps ahead. Environmental Science & Policy.

[CR25] Carlson J, Chappell J (2015). Deepening food democracy.

[CR26] Clancy K, Hammer J, Lippoldt D, Clare HC (2008). Food policy councils-past, present, and future. Remaking the North American food system: Strategies for sustainability.

[CR27] Clapp J (2021). The problem with growing corporate concentration and power in the global food system. Nature Food.

[CR28] Clark JK, Marquis C, Raja S, Knezevic I, Blay-Palmer A, Levkoe CZ, Mount P, Nelson E (2017). The local food policy audit: Spanning the civic-political agrifood divide. Nourishing communities: From fractured food systems to transformative pathways.

[CR29] Clayton ML, Frattaroli S, Palmer A, Pollack KM (2015). The role of partnerships in U.S. food policy council policy activities. PLoS ONE.

[CR30] Crippa M, Solazzo E, Guizzardi D, Monforti-Ferrario F, Tubiello FN, Leip A (2021). Food systems are responsible for a third of global anthropogenic GHG emissions. Nature Food.

[CR31] Dekeyser K, Korsten L, Fioramonti L (2018). Food sovereignty: Shifting debates on democratic food governance. Food Security.

[CR32] Diaz RJ, Rosenberg R (2008). Spreading dead zones and consequences for marine ecosystems. Science.

[CR33] Elstub S, Escobar O (2019). Handbook of democratic innovation and governance.

[CR34] FAO, IFAD, UNICEF, WFP, and WHO (2020). The state of food security and nutrition in the world 2020: Transforming food systems for affordable healthy diets.

[CR35] Fridman J, Lenters L (2013). Kitchen as food hub: Adaptive food systems governance in the City of Toronto. Local Environment.

[CR36] Fung A (2006). Varieties of participation in complex governance. Public Administration Review.

[CR37] Geissel B, Elstub S, Escobar O (2019). Democratic innovations in Europe. Handbook of democratic innovation and governance.

[CR38] Giambartolomei G, Forno F, Sage C (2021). How food policies emerge: The pivotal role of policy entrepreneurs as brokers and bridges of people and ideas. Food Policy.

[CR39] Glennie C, Alkon AH (2018). Food justice: Cultivating the field. Environmental Research Letters.

[CR40] Godek W (2021). Food sovereignty policies and the quest to democratize food system governance in Nicaragua. Agriculture and Human Values.

[CR41] Goetz A, Gotchev B, Richter I, Nicolaus K (2020). Introduction to the special issue: reform or revolution? What is at stake in democratic sustainability transformations. Sustainability: Science, Practice and Policy.

[CR42] Gora A, de Wilde P (2020). The essence of democratic backsliding in the European Union: Deliberation and rule of law. Journal of European Public Policy.

[CR43] van de Griend J, Duncan J, Wiskerke JSC (2019). How civil servants frame participation: Balancing municipal responsibility with citizen initiative in Ede's food policy. Politics and Governance.

[CR44] Hamilton ND (2005). Food democracy II: Revolution or restoration. Journal of Food Law and Policy.

[CR45] Hassanein N (2003). Practicing food democracy: A pragmatic politics of transformation. Journal of Rural Studies.

[CR46] Hasson A (2019). Building London’s food democracy: Assessing the contributions of urban agriculture to local food decision-making. Politics and Governance.

[CR47] Hays CE, Hays SP, DeVille JO, Mulhall PF (2000). Capacity for effectiveness: The relationship between coalition structure and community impact. Evaluation and Program Planning.

[CR48] Henson Z, Munsey G (2014). Race, culture, and practice: Segregation and local food in Birmingham, Alabama. Urban Geography.

[CR49] Horst M (2017). Food justice and municipal government in the USA. Planning Theory and Practice.

[CR50] ILO (2020). COVID-19 and the impact on agriculture and food security.

[CR51] Johnston J, Biro A, MacKendrick N (2009). Lost in the supermarket: The corporate-organic foodscape and the struggle for food democracy. Antipode.

[CR52] Koski C, Siddiki S, Sadiq A, Carboni J (2018). Representation in collaborative governance: A case study of a food policy council. The American Review of Public Administration.

[CR53] Lang T, Koc M, MacRae R, Mougeot LJA, Welsh J (1999). Food policy for the 21st century. For hunger-proof cities: Sustainable urban food systems.

[CR54] Lang T (2005). Food control or food democracy? Re-engaging nutrition with society and the environment. Public Health Nutrition.

[CR55] Lange SJ, Calancie L, Onufrak SJ, Reddy KT, Palmer A, Warnock AL (2021). Associations between food policy councils and policies that support healthy food access: A national survey of community policy supports. Nutrients.

[CR56] Lorenzini J (2019). Food activism and citizens' democratic engagements: What we can learn from market-based political participation?. Politics and Governance.

[CR57] MacRae R (1994). So Why is the City of Toronto concerned about food and agriculture policy? A short history of the Toronto Food Policy Council. Culture & Agriculture.

[CR58] Mah CL, Thang H (2013). Cultivating Food Connections: The Toronto Food Strategy and municipal deliberation on food. International Planning Studies.

[CR59] Mangnus, A.C., J.M. Vervoort, S.R. McGreevy, K. Ota, C.D.D. Rupprecht, M. Oga, and M. Kobayashi. 2019. New pathways for governing food system transformations: A pluralistic practice-based futures approach using visioning, back-casting, and serious gaming. *Ecology and Society* 24(4).

[CR60] McKeon N (2015). Food security governance: Empowering communities, regulating corporations.

[CR61] McMichael P (2014). Historicizing food sovereignty. The Journal of Peasant Studies.

[CR62] Meriluoto T (2021). Struggles over expertise: Practices of politicization and depoliticization in participatory democracy. Democratic Theory.

[CR63] Mooney, P.H., K. Tanaka, and G. Ciciurkaite. 2014. *Food policy council movement in North America: a convergence of alternative local agrifood interests?* Vol. 21. Research in Rural Sociology and Development.

[CR64] Moragues-Faus A (2017). Emancipatory or neoliberal food politics? Exploring the "politics of collectivity" of buying groups in the search for egalitarian food democracies. Antipode.

[CR65] Mounk Y (2018). The people vs. democracy: Why our freedom is in danger and how to save it.

[CR66] OECD (2020). Innovative citizen participation and new democratic institutions: Catching the deliberative wave.

[CR67] O’Flynn I, Elstub S, Escobar O (2019). Democratic innovations and theories of democracy. Handbook of democratic innovations and governance.

[CR68] Owen D, Smith G (2015). Survey article: Deliberation, democracy, and the systemic turn. Journal of Political Philosophy.

[CR69] Petticrew M, Roberts H (2006). Systematic reviews in the social sciences: A practical guide.

[CR70] Pothukuchi K, Kaufman JL (1999). Placing the food system on the urban agenda: The role of municipal institutions in food systems planning. Agriculture and Human Values.

[CR71] Prové C, de Krom MPMM, Dessein J (2019). Politics of scale in urban agriculture governance: A transatlantic comparison of food policy councils. Journal of Rural Studies.

[CR72] Renting H, Schermer M, Rossi A (2012). Building food democracy: Exploring civic food networks and newly emerging forms of food citizenship. The International Journal of Sociology of Agriculture and Food.

[CR73] Roberts W, Blay-Palmer A (2010). Food policy encounters of a third kind: How the Toronto food policy council socializes for sustain-ability. Imagining sustainable food systems: Theory and practice.

[CR74] Rocha C (2009). Developments in national policies for food and nutrition security in Brazil. Development Policy Review.

[CR75] Ryan M, Elstub S, Escobar O (2019). Comparative approaches to the study of democratic innovation. Handbook of democratic innovation and governance.

[CR76] Ryan M, Smith G, Grönlund K, Bächtiger A, Setälä M (2014). Defining mini-publics. Deliberative mini-publics: Involving citizens in the democratic process.

[CR77] Sadler RC, Arku G, Gilliland JA (2015). Local food networks as catalysts for food policy change to improve health and build the economy. Local Environment.

[CR78] Santo R, Moragues-Faus A (2019). Towards a trans-local food governance: Exploring the transformative capacity of food policy assemblages in the US and UK. Geoforum.

[CR79] Saward M, Dryzek JS, Honig B, Phillips A (2006). Democracy and citizenship: Expanding domains. The Oxford handbook of political theory.

[CR80] Schäfer A (2012). Consequences of social inequality for democracy in Western Europe. Zeitschrift Für Vergleichende Politikwissenschaft.

[CR81] Scharpf FW (1997). Economic integration, democracy and the welfare state. Journal of European Public Policy.

[CR82] Schiff R (2008). The role of food policy councils in developing sustainable food systems. Journal of Hunger & Environmental Nutrition.

[CR83] Siddiki SN, Carboni JL, Koski C, Sadiq A-A (2015). How policy rules shape the structure and performance of collaborative governance arrangements. Public Administration Review.

[CR84] Sieveking A (2019). Food policy councils as loci for practising food democracy? Insights from the case of Oldenburg, Germany. Politics and Governance.

[CR85] Smaal SAL, Dessein J, Wind BJ, Rogge E (2021). Social justice-oriented narratives in European urban food strategies: Bringing forward redistribution, recognition and representation. Agriculture and Human Values.

[CR86] Smith G (2009). Democratic innovations: Designing institutions for citizen participation. Theories of Institutional Design.

[CR87] Smith G, Elstub S, Escobar O (2019). Reflections on the theory and practice of democratic innovations. Handbook of democratic innovation and governance.

[CR88] Smith G, Setälä M, Bächtiger A, Dryzek JS, Mansbridge J, Warren M (2018). Mini-publics and deliberative democracy. The Oxford handbook of deliberative democracy.

[CR89] Spaargaren G, Oosterveer P, Loeber A (2012). Food practices in transition: Changing food consumption, retail and production in the age of reflexive modernity.

[CR90] Springmann M, Clark M, Mason-D’Croz D, Wiebe K, Bodirsky BL, Lassaletta L, de Vries W (2018). Options for keeping the food system within environmental limits. Nature.

[CR91] Swartz H, Santo R, Neff RA, Barling D, Fanzo J (2018). Promoting sustainable food system change adidst inequity: A case study of Baltimore, Maryland. Advances in food security and sustainability.

[CR92] Termeer CJAM, Bruinsma A (2016). ICT-enabled boundary spanning arrangements in collaborative sustainability governance. Current Opinion in Environmental Sustainability.

[CR93] Thompson D, Johnson KR, Cistrunk KM, Vancil-Leap A, Nyatta T, Hossfeld L, Rico Méndez G, Jones C (2020). Assemblage, food justice, and intersectionality in rural Mississippi: The Oktibbeha Food Policy Council. Sociological Spectrum.

[CR94] Wada Y, van Beek LPH, van Kempen CM, Reckman JWTM, Vasak S, Bierkens MFP (2010). Global depletion of groundwater resources. Geophysical Research Letters.

[CR95] Zerbian, T., and E. de Luis Romero. 2021. The role of cities in good governance for food security: lessons from Madrid’s urban food strategy. *Territory, Politics, Governance*.

