# Supplementary material for: Power to the people? Food democracy initiatives’ contributions to democratic goods
Source: Agric Human Values. 2022 Jul 6;39(4):1477–89. doi: 10.1007/s10460-022-10322-5 (PMC9258474; doi:10.1007/s10460-022-10322-5)
Supplement: Supplementary file 1 — Supplementary file1 (DOCX 12 kb) [file 10460_2022_10322_MOESM1_ESM.docx]

**Online resource 1 Search strategy**

**Query:**

TITLE-ABS-KEY ( “food democrac*” OR “food policy council” OR (“citizens tribunal” W/20 *food*) OR (“participatory governance” W/20 *food*) OR (“collaborative governance” W/20 *food*) OR (“food sovereignty” W/20 democra*) OR (“food justice” W/20 democra*) )

Search performed on 27 May 2021

**Inclusion criteria:**

1. Written in English

2. Include journal articles and book chapters; exclude conference papers and other outputs

3. Describes or analyses the practical functioning of one or more concrete democratic innovation(s) (i.e. arrangements or initiatives) in the food governance domain

**Exclusion:**

1. Other languages

2. Conference articles, books

3. Democratizing movements etc. (internal working/ procedures of civil society movements)

4. Market-based types of democratization
