# Supplementary material for: Power to the people? Food democracy initiatives’ contributions to democratic goods
Source: Agric Human Values. 2022 Jul 6;39(4):1477–89. doi: 10.1007/s10460-022-10322-5 (PMC9258474; doi:10.1007/s10460-022-10322-5)
Supplement: Supplementary file 2 — Supplementary file2 (DOCX 164 kb) [file 10460_2022_10322_MOESM2_ESM.docx]

**Online resource 2 Data extraction matrix**

| **Authors, year, title of publication** | **Locus + period** | **Type of DI** | **Conceptual and methodological approach** | **Inclusiveness**  The ability of citizens from across different social groups to evenly participate in political decision-making. Includes both formal characteristics of selection mechanisms and the extent to which in practice institutional inducements motivate the engagement of citizens from across groups, so as to avoid marginalization or exclusion. | **Popular control**  The degree that participants are afforded increased influence and control within the decision-making process, covering problem definition, option analysis, option selection and implementation. | **Considered judgment**  The capacity of citizens to make thoughtful and reflective judgments, including understanding of both the technical details of the issue under consideration and the perspective of other citizens. | **Transparency**  The openness of proceedings to both participants and the wider public. |
| --- | --- | --- | --- | --- | --- | --- | --- |
| Andreola et al., 2021, Urban Food Strategy in the Making: Context, Conventions and Contestations | Trento, Italy; 2019-2020 | Food Policy Council: Nutrire Trento (also referred to as Round Table). Jointly convened by the Municipality and the University of Trento. | Economics of conventions approach in the context of building a local food policy.  Multi-method qualitative approach combining in-depth interviews, document analysis and direct observations of construction Trento’s Urban Food Strategy. | On the other hand, the Alternative Food Networks in Trento appear very limited to specific cliques of the population and less inclined to dialogue with other local entities in order to further spread sustainable practices among new producers or consumers. Therefore, they tend to take those closed and self-referential  approaches, with little impact on the local food chain. This trait emerged over and over again during the meetings of the Tavolo di Nutrire Trento, the decision-making board of the city’s participatory project.  Jointly convened by the Municipality and the University of Trento, the Round Table is  an informal space in which all stakeholders can spontaneously participate. The working  group currently involves producers, firms, researchers, professionals, schools, associations,  and groups of citizens and is constantly looking for new stakeholders. The Round Table has  inclusive governance, as revealed by interviews with the participants themselves, which  allows all interested stakeholders to participate in meetings, discuss and make proposals.  In their attempt to bring together as many local agri-food actors as possible, the project  partner institutions decided not to exclude conventional agricultural producers from the  initiative. This allowed 125 actors from the area to participate in the meetings, with a  constant flow of new stakeholder, but it also determined the withdrawal of some “purist”  producers and activists who demanded the adoption of organic as the main criterion.  The existence of an environmental convention that is not shared by all the actors in the  Round Table became explicit with the decision on the criteria for admission to the Nutrire  Trento platform.  The enlargement of the network to non-organic producers was certainly a central  decision of the project. It opened a door for professional associations that do not embrace  alternative production methods, or at least that are not based exclusively on them, such as  Coldiretti, CIA, and ACLI Terra. However, it has led to the distancing of many other actors,  especially those belonging to solidarity purchasing groups. This was underlined by several  interviewees. | Decision-making body of the city’s participatory food strategy project.  It started by stimulating a public debate on key  issues related to food and the opportunity to build an urban strategy for food, bringing  together actors from the food sector. This was made possible by three events in 2017.  These initiatives aim to create a new awareness among participants, not only on the issues  involved, but also on the experiences already existing in the area, and to involve them in  the construction of the Nutrire Trento project. Therefore, a multilateral round table was  established where all the players in the food system could discuss, share initiatives, and  identify new objectives, working together with the institutions.  The Round Table has various functions. It is the consultative board deciding the  criteria for joining the project and the platform, which is designed to coordinate and  increase the visibility of on-going initiatives in the region.  The Round Table prepares thematic events and produces information and promotional  material, such as the conference “Food, Territory, and Sustainability. New food strategies  and local policies to feed cities” on 15th and 16th November 2019. These initiatives aim to  contribute to the development of new awareness among citizens on the values of agriculture  and local food, including those promoted by the so-called Alternative Food Networks,  and to renew and strengthen the relationship between the agricultural sector and urban  community. | What seems to be profoundly linked to this situation, and especially with the inflexibility  of the supply chain, is the lack of a common interpretative key to these phenomena  among the actors seeking change. This can also be seen in their participation in the meetings  of the Nutrire Trento Table.  There is no dominant convention that can coordinate the interpretations of the stakeholders  and the efforts of the Table, which is inevitably slowed down and weakened. In each of the dimensions of the context—economic, political, cultural, and environmental—  different and contrasting frames emerge showing a fragmented scenario.  Within the economic theme, a very divisive topic emerges for local stakeholders,  i.e., the issue of cooperatives. Local stakeholders are aware of the importance that the  cooperative model has had in the history of Trentino. Nevertheless, a fracture has occurred  among the participants of the Table: producer representatives repeatedly recognize the  fundamental role that cooperatives have played in guaranteeing stability and prosperity to  the agro-food sector. Food activists and small-scale producers, on the other hand, seem to  identify some problematic dimensions in the cooperative model that hamper the sector’s  transition toward a more sustainable future and to the adoption of methods perceived as  better, healthier both for consumers and the environment.  In fact, the Trentino food chain has a production structure that puts economic sustainability  before the environmental one. In addition, stakeholders are not able to find  a sufficient degree of consensus on the interpretation of local issues and therefore no dominant convention emerges. Producers’ representatives seem to adopt a civic convention,  focusing on the positive contribution of the cooperative system to the prosperity  of the agricultural sector and on the benefits it has given to small farming communities  in Trentino. Activists and some small-scale producers, on the other hand, adopt an environmental  convention, talking about the problems related to monocultures spread by the cooperative model. Even more, they partially contest the social benefits brought by this model: although they acknowledge some merits in terms of profitability of the sector, the cooperative system has also caused real distortions through the verticalization of the relationship with producers and the subsidiary policy.  A direct relationship would probably involve sharing of knowledge that would help  achieve widespread awareness of each other’s needs and criticisms. Logically, this would  facilitate the implementation of systemic corrections.  However, what emerges from the interviews is that the division between the two sides  of the chain is not only due to divergent positions of the various stakeholders but also  due to a certain self-celebratory narrative that describes Trentino as an idyllic, flawless,  and peerless place. Although the general situation is probably better than other Italian  realities, as we have seen in the chapter dedicated to the context, there are quite a few  problems and the interviewees themselves note this discrepancy.  The combined effect of poor interaction between the parties and the exaltation of a model that in reality presents critical issues may have strengthened Trentino’s structural  inflexibility. In this case, there is no discordant interpretation among the respondents, but  between them and the rest of the population. |  |
| Baldy & Kruse, 2019, Food Democracy from the Top Down? State-Driven Participation Processes for Local Food System Transformations towards Sustainability | Two smaller cities in Southern Germany (names not given); ? | Civil dialogues; expert dialogue | Framing: how local actors frame government-initiated participation processes as processes of food democracy.  Transdisciplinary approach. Participatory observations and semi-structured interviews with involved actors. | Civil dialogues invited all citizens through different media (newspaper, homepage, billboards) to participate and develop measures and goals for the sustainable transformation of the local food system  For the expert dialog, the city administration chose  and personally invited the participants, who represented  local businesses, environmental organizations  and social agencies. This selection process  was not made transparent by the city administration.  However, it became obvious that the administration  had selected people who seemed to be  relevant actors for urban development and with  whom they already had experience of positive  cooperation.  Steering group made up of personally invited administrative staff and members of the city council.  Another transparency issue was who participated in  the three formats; interviewees emphasized that it was  important for participants to know who else would or  would not engage. This also helped participants to reflect  on their own role within the participation process.  While some questioned whether they were the right person  to address the issues discussed (Interviewee B25),  others considered themselves to be “chosen” experts  (Interviewee A16) as they had been selected by the hosting  city administration. | Goals and measures developed in both civil dialogues and expert dialogues; in a closing meeting these were combined and discussed in order to prepare a final list for the steering group. Steering group discussed the goals and measures proposed by the civil and expert dialogues and selected or modified these before voting on them (in official city council meetings).  In both cities, many interviewees did not expect much efficacy  from the participation processes in general. Some  expected the outcome to become a “drawer concept”  (Interviewee A13), representing the interviewees’ fear  that the outcomes would metaphorically disappear into  a drawer rather than be implemented in practice. Others  were explicitly skeptical about the quality of the result  and its capacity to achieve changes in the local food system  (cf. Interviewees B5, B15). One reason for limited  expectations is a perceived deficit within the participation  process, especially concerning the mutual exchange  of knowledge and ideas. Other reasons involve negative  experiences with previous participation processes:  “I have already accompanied many such projects. And  apart from a lot of foam there is usually nothing left”  (Interviewee B9, cf. B24). Finally, the city administrations  initiating the processes were not expected to have  enough influence on relevant policy levels or on significant  sectors, for instance on agricultural policy.  Despite the identified problem framing concerning  a perceived lack of agency, city authorities are considered  pivotal for affecting change through participation  processes. Interviewees from city authorities and from  other stakeholder groups emphasized that the administration  has a role-model function for local citizens: “if  we don’t manage to change the school meals in our  canteen…where the city has a direct influence… then  we have failed” (Interviewee A24). Though sustainable  food system transformation at the local level is a relatively  new issue for public actors, many interviewees demanded  that the city administration use its scope for action,  e.g., in the field of school nutrition and public procurement,  to achieve increasing sustainability or at least  increasing regionality within the local food system. | Interviewees emphasize that mutual exchange of knowledge  about local food systems is an important basis for  true deliberation within the participation processes. This  not only involves sharing different forms of knowledge  among diverse food-related actors who would otherwise  not come together (e.g., members of urban gardening initiatives,  city planners, farmers, cooks and heads of nursing  homes), but it also addresses the problem that lacking  knowledge about the local food system hinders actors  from participating in transformation processes in the first  place and risks excluding issues relevant to a sustainable  food system: “We were not aware that…[food] was also  a topic that…had a very important influence on our CO2  emissions” (Interviewee A17). This quote illustrates that  not all members of the city administration were even  aware of the climate impacts of food before starting the  participation process. Meanwhile, the analysis revealed  a high level of contestation among participants regarding  what issues should be included in mutual knowledge exchange.  Additionally, some interviewees were very open  to mutual learning, while others assumed that they already  had all the relevant knowledge about sustainable  food system transformations.  During the participation processes, the question  was raised as to which actors can legitimately organize  and take part in the participation process and thereby  gain the chance to bring their knowledge into the deliberation  of ideas. Connected to these questions is the  credibility of knowledge. Interestingly, actors from local  food businesses as well as civil society actors question  the credibility of the knowledge claims of the other party,  accusing them of hidden goals or values. While interviewees  from civil society organizations assume that local  entrepreneurs are only engaging because they expect  personal profit, interviewees from local businesses assume  that civil society actors advocate growth-critical positions.  For many interviewees, two types of knowledge  were especially credible: knowledge of like-minded participants  and scientific knowledge, especially if this could  be used to underpin their own opinions.  The second relevant aspect for sharing ideas on local  food system transformation is a shared language. It  was criticized that the language used during the participation  process was partly too scientific and hindered the  engagement of citizens and other stakeholder groups.  One interviewee  revised his assumption about his co-participants  who he had initially described as being oriented by  self-interest (Interviewee A16). This shows that learning  takes place within participation processes, which can increase  credibility and trust among diverse and partly opposing  participants. | The  analysis shows that many interviewees consider openness  and the exchange of issues and ideas at eye level  to be important prerequisites for true deliberation and  dialog, thereby criticizing rigidly structured, one-way procedures.  Another emphasized prerequisite for mutual exchange  is transparency in both the design and outcomes  of the debate. |
| Bassarab et al., 2019, Finding Our Way to Food Democracy: Lessons from US Food Policy Council Governance | United States; 2018 | Food policy councils | Examines the relationship between FPCs’ organizational structure, relationship to government, and membership and its priorities.  2018 survey of FPCs in the United States. Descriptive statistics, quantitative analysis and three illustrative cases. | Most FPCs (71%) focus  their work at the local level: city/municipality, county,  or both city/municipality and county. Only 8% of FPCs  work at the state level and 22% work at the regional level  (multiple counties). Additionally, the majority of FPCs are  embedded in an institution: 35% are sponsored by a nonprofit  organization, 29% are embedded in government,  and 6% are housed in a university. Another 18% are unincorporated  (grassroots) groups and 12% are nonprofit  organizations (Table 1).  Being embedded in government is only one way for  FPCs to connect with government. An FPC may be created  by legislation (17% of FPCs); include government employees or elected officials as members1 (86%); have  members appointed by government (21%); receive inkind  support—meeting space or administrative help—  from government (40%); or receive funding from government  grants or appropriations (35%)―Table 2. We also  account for FPCs that have no relationship to government.  FPCs’ membership consists of professional stakeholders,  public administrators, and elected officials from across  the food supply chain and interrelated issue areas, e.g.,  environment, education, economic development, and  health care. FPC membership also includes lay stakeholders,  referred to as community members in the survey.  FPCs could report their membership composition by selecting  as many membership categories represented on  their council as necessary (Table 3). On average, FPCs  selected 10.84 (standard deviation 3.55) of the total 19  membership categories, with a range from 1 to 19. The  membership of the majority of FPCs (92%) includes a  community member. All but two FPCs have representation  from professional sectors.  [Illustration 1:]  Adams County, Pennsylvania, is a mostly rural county  with an estimated population of just over 100,000 in  2017. The main town in the county, Gettysburg, had a  population of around 7,600 in 2017. While the Adams  County Food Policy Council (ACFPC) was established  through a county proclamation in 2009, it is housed  within Healthy Adams County, a nonprofit organization.  The FPC’s structure is non-hierarchical; there is no official  leader, but logistics and meetings are coordinated by a  facilitator. While membership is self-selecting and open  to anyone who wants to participate, it mainly attracts  professionals from government, academia, health care,  and nonprofits working on related issues. Approximately  12 to 15 people regularly attend meetings in a volunteer  capacity. Community input is sought periodically during  a forum whereby anyone who wants to participate is invited  to share their opinions and outreach to community  members who receive services from organizations that  participate in the ACFPC. This input informs the ACFPC’s  actions, although there is not a formal process for determining  policy priorities.  [Illustration 2:]  Baltimore is the largest city in the state of Maryland  and has a majority African American population. Housed  within the city government and funded by the city, the  Baltimore Food Policy Initiative (BFPI) engages government  staff, elected officials, and professional and lay  stakeholders through three approaches: 1) intra-agency  collaboration, 2) the Food Policy Action Coalition (FPAC),  and 3) the Resident Food Equity Advisors (RFEA). The  City’s Food Policy Director and two staff housed in the  Planning Department work with other government staff  throughout the city on food systems issues.  FPAC is an open network of more than 60 selfselected  people, mostly professionals from area nonprofit  and community organizations, businesses, as well  as university faculty and students whose work intersects  with food systems. The network meets quarterly. This  format allows for participation from a wider network of  actors, but those who choose to participate in FPAC do  not reflect the majority of Baltimore’s population and  are generally not people directly affected by food system  problems (Swartz, Santo, & Neff, 2018).  ...  Recognizing the need for a more deliberate means of  eliciting residents’ perspectives, BFPI formed the RFEA in  2017. Supported by a stipend, one resident from each  council district in the city is selected through an application  process to serve as an adviser to BFPI staff on food  system issues facing the city. Over the course of six meetings,  RFEA and BFPI staff convene to hear presentations  from content experts, converse about their experiences  related to the issue, deliberate about solutions, and craft  appropriate policy recommendations. While BFPI staff  make the final decision about recommendations, they  are there to learn from residents about what they think  will work. Advisers are invited to meet with their elected  council member to share their experiences and to talk  about the group’s recommended food policy strategies.  By recognizing and integrating the voices of those most  affected by food policy decisions into their work, BFPI is  facilitating a transition in democratic participation from  representational to participatory democracy.  [Illustration 3:]  Austin, the state capital of Texas, is home to nearly  a million people. Established in 2008 through local ordinances,  the Austin Travis County Food Policy Board  (ATCFPB) is a 13-member advisory board for the City  of Austin and surrounding Travis County. Board members  are appointed by the City Council and the County  Commissioners. Members are encouraged to represent  a diversity of sectors across the food supply chain  but there are no required sector-specific positions.  Appointed members decide which policies to recommend  to local government staff and elected officials. The  Board is supported by four working groups with open,  self-selected membership and coordinated by City and  County staff. Working groups serve as technical advisers  to the Board around specific food systems issues. Citizens  are invited to participate in a working group or to express  their opinions during an allotted time at the beginning of  the Board’s monthly meetings.  This study shows that members matter; membership  is related to a wide range of policy priorities. Some policy  priorities have significant relationships with multiple  membership categories. For example, the priority of food  waste and recovery bears significant relationships with  members representing anti-hunger organizations (recipients  of excess food), food processing and distribution  (involved in coordinating logistics for delivery of excess  food), food waste, health care (for whom hunger and  health are intricately linked), natural resources and environment  (for whom wasted food represents wasted  water and greenhouse gas emissions), and philanthropy.  These relationships align with national campaigns to reduce  food waste. This alignment raises questions about  the degree to which national efforts influence the decisions  of FPCs and whether FPCs can serve as a gateway  to increase civic engagement at a national level.  Additionally, for some membership sectors, there is  a relationship between the policy priority and the sector  that the member represents. For instance, FPCs with  members representing economic development are more  likely to prioritize economic development policies while  those with farmers, ranchers, or small producer advocacy  groups are more likely to prioritize food production  policies. The relationships between sector representation  and policy priorities are of particular interest in con-sideration of whose values are being represented by the  FPC. In an analysis of two local government planning processes,  Baldy and Kruse (2019) point out that the values  represented by members participating in the processes  are complex and varied and based both on their personal  experiences and professional role. Further research is  needed to understand if members are representing the  interests of their employer, an area of the food system  for which they have a deep interest, or a particular demographic  of the community.  FPCs struggle with community representation both  in determining who counts as community members and  how community members are included in FPC decisions.  While most FPCs report to have members that represent  the community, it is often unclear how FPCs define  community members. Do they include lay stakeholders  with a vested interest in an issue and willing to  participate for free, people affected by problems with  the food system, socially marginalized communities, lowincome  communities, minority populations, or people  traditionally excluded from economic and political processes?  A few lay stakeholders amongst a dozen paid professionals  could cause an imbalance in decision-making.  Collaborative governance approaches can be designed  to build the capacity of members at a disadvantage  (Emerson & Nabatchi, 2015).  Not all mechanisms for participation are equal, but  the survey used for this article does not collect information  about member selection (open, self-selecting,  or targeted recruitment) or authority (voting members  of a steering committee or non-voting expert advisers  of a working group). These distinctions are important  to understand the power dynamics among members in  setting the policy agenda for an FPC. For instance, the  ACFPC and the BFPI Resident Food Equity Advisors use  an open, self-selecting process for member participation,  yet mostly paid professionals participate. Such a format  poses the risk that members are recruited through existing  networks, which could inhibit inclusiveness and  exacerbate inequity and underrepresentation of certain  groups. Additionally, some members, such as professionals,  may be perceived to have more expertise or authority  because they have more resources and capacity.  They may also represent the priorities of organizations  outside of the community. Further, as Sieveking  (2019) found with the Oldenburg FPC in Germany, openness  of membership, and the resulting diversity, can bog  down decision-making processes. Allowing elected officials  to appoint members, such as the ATCFPB however,  could also exclude stakeholders who traditionally lack  economic capital, knowledge, or capacity to engage in  the policy process. FPCs struggle to find a balance between  harnessing the energy of those eager to transform  food systems, learning from those with food systems  and policy expertise, and empowering those impacted  by food systems issues.  A membership process that deliberately places those  who experience the effects of policy decisions in a position  to influence policy can add to the effectiveness  and equity of policy decisions (Fung & Wright, 2001). The  BFPI Resident Food Equity Advisors program elevates the  authority of residents in food policy decisions by specifically  selecting residents to work directly with local government  staff on policy recommendations. The ACFPC  also tries to elevate the perspectives of residents who  are affected by food systems issues in the FPC’s decisions  and actions through direct outreach to them. Our findings  reinforce those of the Koski et al. (2018) case that  the engagement process is important to ensure member  representation on paper, in practice, and in the selection  of policy priorities. | This article focuses on the policy priorities  of an FPC because the policy priorities drive where  an FPC invests its resources. Policy priorities reflect the  food systems issues that FPC members identify as critical  to address collectively. Policy outputs (e.g., legislation)  and policy outcomes (e.g., individual health changes) can  take years to achieve (Sabatier & Jenkins-Smith, 1999)  and are difficult to track and measure for one FPC let  alone across hundreds of FPCs. The outcomes of specific  policy changes are especially hard to measure due to  the complexity of interrelated elements across a system.  These challenges with policy outputs and outcomes explain  why the most comprehensive dataset available on  FPCs, used for this article, only tracks FPC policy priorities.  Our findings on the relationships between an FPC’s form  and policy priorities from the bivariate analysis, shown  in Table 5, demonstrate that membership composition  and relationship to government have more bearing on  the policy priorities of an FPC than the organizational  structure. Organizational structure has one significant relationship  to policy priorities, namely if an FPC is a grassroots  coalition, embedded in a nonprofit, or a nonprofit  itself, it is more likely to have policy priorities around production.  In contrast, both membership and relationship  to government have several significant relationships with  policy priorities.  As Table 6 shows, most types of relationships that  an FPC has with government have inverse relationships  with some policy priorities. Seven out of the ten significant  relationships suggest that FPCs put less priority on  certain issues. In other words, having a relationship with  government is related to what FPCs do not prioritize.  For example, FPCs that are embedded in government,  have government support (in-kind or financial), or have  government-appointed members are less likely to prioritize  food production policy issues. Conversely, FPCs with  no connection to government are more likely to prioritize  food production.  Overall, the relationships between a membership category  and the corresponding policy priority are positively  and significantly correlated (Table 7). Appendix A pro-vides a full reporting of all relationships. The priorities of  food waste/recovery, anti-hunger/emergency food, and  land use planning have more significant relationships to  membership categories than other priorities. One explanation  is that some policy priorities are common across  all FPCs (e.g., healthy food access) or prioritized overall  by very few FPCs (e.g., land use planning). Additionally,  some membership categories are related to more policy  priorities than others. For example, having members representing  faith-based organizations is significantly correlated  to three of the seven policy priorities while  members representing anti-hunger/emergency food are  correlated with two policy priorities. Government staff,  elected officials, and community members are not correlated  with any policy priorities. Because nearly all FPCs  have community members, we would not expect any  significant differences in relationship to policy priorities  across councils.  [Illustration 2:]  Quarterly  meetings consist of formal presentations and informal  networking, whereby FPAC members learn from one another  and share ideas with BFPI staff on current policy  issues. FPAC members also approach BFPI staff as policy  issues arise because of relationship building efforts  by BFPI staff outside of the meetings. FPAC members’ input,  along with BFPI staff’s own assessment of the feasibility  of changing a policy, help BFPI select which policies  to focus on. In this interaction, FPAC fulfills a comple-  Politics and Governance, 2019, Volume 7, Issue 4, Pages 32–47 38  mentary role as described by Klijn and Skelcher (2007) to  BFPI, allowing professional and lay stakeholders to listen  and learn about policy issues in the city and to have their  “say” in providing advice in the policy process.  ...  Over the course of six meetings,  RFEA and BFPI staff convene to hear presentations  from content experts, converse about their experiences  related to the issue, deliberate about solutions, and craft  appropriate policy recommendations. While BFPI staff  make the final decision about recommendations, they  are there to learn from residents about what they think  will work. Advisers are invited to meet with their elected  council member to share their experiences and to talk  about the group’s recommended food policy strategies.  An FPC’s relationship to government also bears a  number of significant relationships to its policy priorities.  Unlike membership, however, there is a significant inverse  relationship between food production and an FPC’s  relationship to local government. One plausible explanation  is that many regulatory issues (e.g., crop insurance,  organic certification, food safety) facing farmers  and ranchers are the result of federal policy decisions.  While some FPCs do follow and advocate on federal policies,  participation in the federal policy process requires  different strategies and diminishes the effectiveness of  policies to address place-based problems.  The aim of FPCs to create greater pathways for citizen  participation in the policy process can only be  achieved with government cooperation. Elected officials  and public administrators must be willing to engage in colearning,  deliberation, and power-sharing with members  of society, as shown in the case of the BFPI RFEA program.  The effectiveness and sustainability of an FPC as a collaborative  governance network is questionable without this  willingness. Other scholarship on FPCs has shown that  an unwillingness by government staff to make the policy  process accessible to citizens and attempts to impose  the agenda of appointed or elected officials on an FPC  can reduce the effectiveness or lead to the dissolution of  FPCs (Coplen & Cuneo, 2015; Gupta et al., 2018; van de  Griend, Duncan, & Wiskerke, 2019). | [Illustration 3:]  As appointed representatives, Board members must  navigate suggestions from self-selected members of  working groups, the preferences of a broader network,  as well as their own professional and personal interests.  This balancing act led the Board to punt on a decision  on a paid sick leave bill proposed by the Austin City  Council. Faced with a recommendation from the Healthy  Food Access Working Group to support the bill, as well  as mixed recommendations to support and not support  the bill from business and community organizations from  the wider Austin community, the Board decided to neither  support nor oppose the bill. The bill, however, was  eventually passed by Austin City Council because of overwhelming  support expressed by attendees at a community  input meeting held by the Austin City Council (Morrill  et al., 2018). At the center of the debate was a complex issue  steeped in opposition from businesses that are better  resourced and have more access to public officials than  other stakeholders, such as workers and their representatives.  Collaborative governance networks are prone to imbalance  if there is not a way to equalize the capacity, resources,  authority, and status of members (Ansell & Gash,  2008). The ATCFPB tried to balance the views of proponents  from the working group and community organizations  with opposing views from Board members and businesses  through a null vote. This vote held the ATCFPB accountable  to both the Board and the working group but  ultimately did not represent the desire of the community.  Organizational structure is not an irrelevant factor  for FPCs, but our findings echo DiGiulio’s (2017) findings  that it does not drive policy decisions. An FPC’s organizational  structure can influence membership composition  and the power dynamics amongst members, particularly  for FPCs embedded in government or where members  are appointed. In the case of the ATCFPB, the members  appointed to the Board by local elected officials represented  certain interests (e.g., businesses) that had opinions  contrary to the desires of the community. For BFPI,  an FPC embedded in government, determining its policy  agenda and strategies often depends on political timing  and agency collaboration and readiness. |  |
| Blay-Palmer, 2009, The Canadian Pioneer: The Genesis of Urban Food Policy in Toronto | Toronto, Canada; 1990-2009 | Food policy council | Thick description of the inception of the TFPC.  Interviews, personal observation, personal notes TFPC member. | The TFPC is a sub-committee of the Toronto Board of Health. The Council is run by  Acting Manager Dr. Wayne Roberts, who is supported by one-and-a-half full-time staff  and 20 volunteer council members. Until the fall of 2008, Roberts’ position was nonmanagerial  and was supported by a half-time administrative assistant. Roberts assumed  his role at the TFPC in 1996. The TFPC coordinator has always had to rely heavily on  its volunteer board members. The volunteer board includes a mix of city councillors  and citizen experts. The current slate has community activists, physicians, gardeners,  academics, UN advisors, immigrants, farmers, conservationists, planners and refugees.  The breadth of board member experience allows the TFPC to provide authoritative,  credible input on an extensive range of food-related issues, such as:  ...  As MacRae observes:  FPC was an intermediary between citizen input and City Council. We didn’t run big  consultations, but we did orchestrate deputations at the Board of Health and City  Council. We were really an organization of organizations. So this was in some  people’s view an insulating layer, but realistically not that many citizens have the  skill to participate in these kinds of processes, but we did attract those with such  skills. The anti-poverty agenda was always front and centre. It was clear that we  wouldn’t survive if we weren’t having some impact on food access for low-income  citizens; hence the early focus on welfare reform, Field to Table and school food.  Entering the new millennium, the FHAC and TFPC were looking for positive food  security initiatives to unite the city under a food umbrella. The TFPC/FHAC secured  $300,000 as part of their efforts to turn policy into practice. This money was allocated  to advocacy, animation and targeted community development. As the first step towards  action, they solicited community grant applications. Disappointingly, there were no applications  from the most challenged communities. This sparked the insight that community  capacity building was needed so communities in need could apply for grants. The TFPC  moved to help groups develop the skills and tools to write grant applications. It also  became clear that to be able to apply for grants, communities needed to move beyond  surviving to taking responsibility for community assets. This then empowered communities  to take the next step and be able to identify their own needs. This allowed disadvantaged  groups to articulate their needs. In turn, the city could understand their challenges  and meet them half way. | The Toronto Food Policy Council has a small staff and modest budget. It has no  authority to pass or enforce laws. It’s the power of ideas, inspired individuals and  empowered communities that give us influence.  The Council is free to make its own decisions on food policy issues. Staff working  with the TFPC is employed by, and responsible to, Toronto Public Health. This innovative  and effective arrangement has gained international respect from public health,  community food security and sustainable agriculture organisations (TFPC, 2009a).  According to one TFPC member:  [T]he way the TFPC is structured, it is a council of citizens. We come with our own  opinions, not organizations. It is a place where you can think independently of any  other opinions. It is very positive. We have a thorough knowledge of food issues  while everyone brings their own expertise, very diverse. We look at food issuesfrom our life experience regarding food. . . The council does not write policy, it  can only give advice on policies, does not have the power to enact policy, that is a  challenge.  Maintaining funding and profile for sustainable food issues and the TFPC is difficult.  There is still substantial push-back from nutritionists, environmentalists, planners and  other city bureaucrats about the importance of food and the need for a holistic approach  to policy. For example, despite having food in the official plan, planning officials need  constant reminders about its relevance and linkages. There is also the need to educate  and maintain leadership on food issues. This requires the TFPC to adopt an inclusive  approach that frequently brings conflicting interests to the same table. Hence, facilitating  dialogue is another role played by the TFPC. | According to one TFPC member:  [T]he way the TFPC is structured, it is a council of citizens. We come with our own  opinions, not organizations. It is a place where you can think independently of any  other opinions. It is very positive. We have a thorough knowledge of food issues  while everyone brings their own expertise, very diverse. We look at food issuesfrom our life experience regarding food. . . The council does not write policy, it  can only give advice on policies, does not have the power to enact policy, that is a  challenge. |  |
| Boossabong, 2017, Floods and Food in the City: Lessons from Collaborative Governance Within the Policy Network on Urban Agriculture in Bangkok, Thailand | Bangkok, Thailand; 2010-2012 | Collaborative governance network on urban agriculture | Collaborative governance within policy networks  Observation of 11 communicative forums; interviews with 161 representatives of 64 constituent organizations and groups; three focus groups with core organizations and groups | This  network was formed in 2010 within the City Farm programme, to promote  an alternative urban food system in Bangkok. The programme  was led by the Health Promotion Foundation—a public organization  under the Prime Minister’s Office—in collaboration with non-governmental  organizations including the Sustainable Agriculture Foundation,  the Media Centre for Development and the Working Group on Food  for Change. They cooperated with a network of social and green enterprises  known as the City Farm Association. The Bangkok Metropolitan  Administration and District Administration Offices, and regional  and local governments also took part in the City Farm programme by  encouraging various communities to develop community gardens. They  also supported training in farming practices by developing the City Farm  Learning Centre, with headquarters in their respective offices.  Other public organizations participating in the UA policy network  included the research units of several public universities supporting the  production of knowledge and dissemination of information on foodgrowing.  The focus of the universities for urban food policy was on  linking food with health, local economy, environment, social welfare  and education. The programme managed to engage various groups of  the urban poor and marginalized citizens, such as the city’s network of  slum dwellers and the informal labour network. These groups were supported  by community-based organizations such as the Human Settlement  Foundation and the Foundation for Labour and Employment Promotion.  The recognition of local knowledge was not only a way to enhance  resilience, but also to promote cultural recognition of the victims,  defined as an aspect of justice. The policy network was able to promote the meaningful participation of victims by including them in the deliberative  process, to exercise their right to define risks and needs and to  propose solutions based on their knowledge.  First,  the organization of communicative fora that were opened to the victims  to raise their voices, without interventions and censorship, in itself promoted  resilience and justice. Through this process, peri-urban farmers  and slum communities located along the river, and their practical knowledge,  were recognized within the deliberative process. | [lists all kinds of actions undertaken; does not become clear how these were decided on precisely, or by whom]  The recognition of local knowledge was not only a way to enhance  resilience, but also to promote cultural recognition of the victims,  defined as an aspect of justice. The policy network was able to promote the meaningful participation of victims by including them in the deliberative  process, to exercise their right to define risks and needs and to  propose solutions based on their knowledge.  In addition, those  affected by the floods developed social safety nets in collaboration with  the policy network, which in turn strengthened their adaptive capacity.  They proposed different ways (from those adopted by central government)  to define risks, such as by comparing water level with their  heights, by explaining food inaccessibility in specific local contexts, and  by addressing particular risks (e.g. recent locations of snakes and crocodiles).  Deliberation and action were interlinked: after discussing problems  and possible solutions, policy network members took action to address  them; and after finishing one mission they started another discussion.  In some cases, deliberation and action took place at the same time  (e.g. seed-sharing events and showcases of food innovation). The UA  policy network was able to contribute to the development of a ‘communicative  solidarity’. Discussions facilitated learning, mutual understanding  and agreement. Furthermore, by discussing the normative  principles that should guide actions, the network was able to build discourse  coalitions—  partnerships of policy network members and victims  having a particular agreement. | As mentioned in the Introduction, the notion of resilience is close to  the Thai local dialect term Yeed Yuin (to be flexible to change), reflecting  a normative principle embedded in Thai culture. As highlighted by  Sivaraksa (2011), to be flexible equates in fact with the Thai style of  living. The discussion between policy network members and the flood  victims also reflected this cultural aspect. Although the flexible way of  life (flexible livelihood) does not mean the same as resilience (at least as  defined in the Western academic world), it is a discursive practice of resilience  promotion endorsed by the policy network. The network’s constituent  organizations and groups referred to Yeed Yuin as a strategy to  recall this Thai norm for developing a discourse coalition. For example,  network members encouraged flood victims to grow what they like to eat and to eat what they grow. They also supported the sharing of adaptive  practices across communities, such as using broken helmets and foam  food-boxes as containers for growing food.  The discussion between UA policy network members and flood victims  also reflected an understanding of resilience and justice (in terms of  the social construction of their discourses) through their expectation of  what a ‘good society’ would look like—‘a dream for a self-reliant society’.  Influenced by the Thai traditional way of life and the King’s speech  on self-sufficiency and low-input farming approaches for self-subsistence,  self-reliance has become over the years the state philosophy. Even if selfreliance  has not yet been achieved, this philosophy has created a sense of  a ‘good society’ that informs the aspirations of many Thai people.  Discussions between policy network members on self-reliant ideology  also brought about their vision that local knowledge should be recognized  and promoted to cope with the disaster. Self-reliance on victims’  own knowledge can be interpreted as a means of enhancing resilience,  because local knowledge was seen as being more sensitive to local problems  and therefore more apt to shape flexible livelihoods. In debates and  interaction between the UA policy network members and the victims  of the flood, ‘sacred knowledge’ (as termed by Habermas et al. 2010)  played a role in healing victims’ anxiety and fear, allowing them to have  a strong sense of self-awareness to cope with the problems. For example,  many victims prayed to the river to protect them by assuming that the  river was their mother. Some policy network members (e.g. the coordinator  of the Media Centre for Development) also gave consolation to a  victim community by mentioning that, she believed, if we pay respect to  nature, nature in return will be kind to us.  The recognition of local knowledge was not only a way to enhance  resilience, but also to promote cultural recognition of the victims,  defined as an aspect of justice. The policy network was able to promote the meaningful participation of victims by including them in the deliberative  process, to exercise their right to define risks and needs and to  propose solutions based on their knowledge. An example of a solution  based on recognized local knowledge was the use of locally made effective  microorganisms to enhance soil quality for growing short-life vegetables  and improving the quality of polluted water.  In addition, those  affected by the floods developed social safety nets in collaboration with  the policy network, which in turn strengthened their adaptive capacity.  They proposed different ways (from those adopted by central government)  to define risks, such as by comparing water level with their  heights, by explaining food inaccessibility in specific local contexts, and  by addressing particular risks (e.g. recent locations of snakes and crocodiles).  Some of them were able to exercise communicative power by forcing  the ‘unforced force of better argument’ (that is persuading others of  the value of a particular idea and bringing them to implement it). Then,  the deliberative agreement reached among policy network members about target groups and strategies brought about collective actions  undertaken in collaboration with those affected by the floods. |  |
| Calancie et al., 2018, Evaluating Food Policy Councils Using Structural Equation Modeling | US, Canada and Tribal & First Nations; 2015 (?) | Food policy councils | Food Policy Council Framework, adapted from a parsimonious community collaborative model that was developed and empirically tested by Allen and colleagues in the context of the system and community response to family violence  Food Policy Council Self-Assessment Tool that asks FPC members about their perceptions of their council; send to two contact persons from each of the 282 councils listed on the 2015 Food Policy Network’s Online Directory; statistical analysis. | Our results show that interventions aiming to strengthen FPCs should be directed toward increasing Organizational Capacity components (leadership, breadth of active membership, inclusivity of council climate, and formality of council structure) since they may increase Council Effectiveness more than efforts directed at increasing Social Capital. Moreover, efforts to increase Organizational Capacity are likely to increase Social Capital. |  |  |  |
| Calancie et al., 2017, Food Policy Council Self-Assessment Tool: Development, Testing, and Results | US, Canada and Tribal & First Nations; 2015 (?) | Food policy councils | Food Policy Council Self-Assessment Tool, see previous | In testing this assessment tool, we found several important patterns.  Most FPC members are white women aged 35 to 65 years.  A study of the relationship between community coalition factors  and community impact found that greater racial diversity was associated  with coalitions’ ability to change public policy and improve  community prevention systems (31). FPCs may consider  strategies for increasing diversity among their members. More research  is needed to determine what member engagement strategies  are effective at increasing member diversity. When looking at the  mean scores for each concept scale, breadth of active membership,  credibility, and impact had the lowest means. These are areas  where councils may choose to direct more energy or seek external  support such as training, technical support, and resources. Councils  with low breadth of active membership scores, for example,  may consider engaging potential members in a variety of settings.  Councils can hold public forums where community members can  provide input on the councils’ activities, or they can boost active  participation by aligning council priorities with members’ goals  and values. |  |  |  |
| Calancie et al., 2017, Food Policy Council Case Study Describing Cross-sector Collaboration for Food System Change in a Rural Setting | Adams County, Pennsylvania, United States; 2014 | Food policy council | Descriptive case study of a rural FPC  8 interviews with FPC members |  | The ACFPC has the important and unique function of developing, supporting, and maintaining food access-related programs that cross multiple sectors and populations in Adams County. The council also acts as a resource for country residents looking to implement food system-related changes in their community.  ACFPC members are working toward policy solutions to address food system issues. Council members described their programs (such as the Healthy Options program) as “filling the gap” where food policy and systems have failed groups in the community. As one council member described, “[our programs] have had significant impact on a small number of people but I think now we’re trying to think a little bigger than that so it’s not small initiatives for couple hundred people but really shifting the way our food systems works.” Council efforts have already led to organizational policy changes, such as accepting EBT at farmers’ markets and influencing food recovery policies at restaurants and grocery stores. Currently, the council is exploring approaches for affecting other food-related policies in their community. One interviewee recommended school policy as one option, although noted that school systems could be difficult to navigate owing to bureaucracy. Other members suggested reaching out to local officials and request that they join or participate with the council in some capacity. ...  Many council members described challenges influencing policy change. Some said that federal-level policies, such as immigration and food safety regulations, affect their local food system. This reality can feel frustrating, because the council is not currently in a position to influence federal policy. One member saw the opportunity in this challenge and said, “I think with very little training and lots and lots of passion, knowledge, study, support and collaborative work, we’ve been able to make some significant community changes. It would be really exciting to figure out how do we lend our voice, or engage and entice other FPCs to join us to figure out where we can make changes at the state or federal level.” Several council members noted that legislators have their own agenda, which can be both a barrier and opportunity for policy change, depending on whether food systems issues can get on that agenda. | Council members commonly cited the egalitarian atmosphere as one of the greatest strengths of the ECFPC. One member noted, “I think people feel that their voices and contributions are really valued.” This tended to be a common thought and was true for most people; “[E]veryone no matter their background, no matter age, we are all heard.” Interviewees attributed their feelings of egaliteraianism to the council’s structure, small size and group dynamics. Several interviewees commented that some personalities within the council occasionally clashed, but that in general everyone “played nice” during council meetings to move the group forward. A strong commitment to the group’s mission seemed to override occasional personal conflicts. Also, council members are volunteers and thus are internally motivated to participate. Although the council does not have an elected or appointed leader, several interviewees referred to another council member as an informal leader. Council members reported that equality within the council “works well” and that the group makes decisions through consensus. Most participants mentioned the importance of the AmeriCorps Vista member acting as a facilitator during council meetings to ensure they run smoothly, set meeting agendas by asking for input from all the council members via email, and to conduct the day-to-day administration of the council. |  |
| Clancy et al., 2008, Food Policy Councils: Past, Present, and Future | North America | Food policy councils | Review of history and performance of government=sanctioned FPCs that have a minimum three-year history of operation  Interviews with key leaders from FPCs; review of relevant literature and archival material | In most councils the members were or are appointed by the mayor or county or state legislatures, and most have ex officio or special representatives from government agencies. The types of groups represented on councils vary. Some have membership that focuses on urban food access, while others include a wider range of farming, health, and environmental interests. Most of the groups have either paid or in-kind staff from agencies such as a department of health or Cooperative Extension or from nongovernmental organizations. Most, except Toronto, have little or no consistent funding.  Council membership ranges from 9 to 24 individuals with an average of 12 to 14 members. Councilors are officially appointed by city, county, state, or other government department leaders; in practice, most additional appointments are made by recommendation of the incumbent councilors. Most councils try to maintain a mix of specific food system stakeholders from such areas as farming and agriculture, anti-hunger, health, food industry (such as processors and retailers), government agencies (such as departments of health and social services), and nonprofit organizations. Several councils include city or country councilors or commissioners, while others mandate representation by specific government departments, either as official or ex officio members.  A final point of advice is inclusion of broad representation from across the food system in the development and operation of the FPC. Respondents cited diversity of food systems stakeholders as key for starting a FPC, even though such broad representation may make narrowing the council focus to a manageable agenda more challenging. | Examining their own words as taken from various resolutions, council goal statements, bylaws, and so on, one can see what FPCs are created to do:  - Advise and make recommendations to state, city, and county government on food policy issues;  - Monitor and evaluate the performance of the local food system  - Foster better communications among all actors in the food system, including policymakers and the public  - Assist residents in understanding the food system and food policy  - Act as a forum for discussions on improving the food supply  - Educate FPC members about each others’ roles and concerns  - Plan and oversee food system projects  - Facilitate research on food issues  Six of the eight councils engage(d) in policy activities of various kinds, although how frequently or visibly depends on the specific council. Especially in the local councils, policy activities have to be done carefully to keep all the members comfortable. For this reason one council engages with administrative processes but not legislative proposals per se. Another made forays into the policy realm but was not successful and has not tried again. In the state councils it has been more of a given that policy recommendations will be on the agenda.  Although only St. Paul explicitly mentioned its role as a network facilitator, many councils engage in such activity as they bring diverse stakeholders together with the result of increased understanding of food system issues and/or new or better programming.  Government sanction is important so as to “get government on record as seeing their role in food planning.” While no respondent claimed to have *the* model legislative language for the creations of FPCs, government sanction was seen as critical to establishing the legitimacy of the councils. Even with the sanctions FPCs still find they struggle to establish their own place alongside other better-established commissions, such as those for water or the environment. |  | Councils are mixed in their interest and ability to garner public attention. Some councils see a lack of public visibility as a liability. However, others do not have visibility as a priority. In fact, those councils see a low public profile as creating a stronger ability to work behind the scenes in less threatening, but more-effective, interaction and networking with government agencies. Most of the councils that would like to crate a stronger public image report limited success. They try to engage the public through programs, events, and public forums but have not often attracted large audiences or local media (Connecticut and Iowa being the major exceptions). ... Ironically, councils with prominent members may find it difficult to gain public recognition as it is “challenging to clarify when the council is being represented as opposed to the individual with overlapping duties and affiliations.” |
| Clark et al., 2017, The Local Food Policy Audit: Spanning the Civic-Political Agrifood Divide | Franklin County, Ohio, United States; 2012-2015 | Food policy council | Advocacy coalition framework  Food policy audit; community-based participatory research | As a result, the Franklin  County Local Food Council was established in September of 2011. It represents  members from the local government, agriculture, education, retail, distribution and  advocacy sectors of the local food system. This council is an all-volunteer grassroots  group with no formal affiliation to local government. | When the food council formed, the overwhelming majority of members did not  want to work on policy issues. Although the Central Ohio Local Food Assessment  and Plan contained five recommendations under the goal of “…removing] policy  barriers to a local food system,” many members of the food council were hesitant  because they understood the goals they set as a council to focus more on practices,  processes and education than on policy (MORPC’s Agriculture and Food Systems  Working Group 2010). In addition, food council members were worried that funders  would be hesitant to fund policy work. This aversion to policy work resulted in  the word “policy” being left out of the food council’s name, in spite of the group  intentionally modeling itself after food *policy* councils throughout the country.  The audit findings and resulting recommendations may have been the turning  point that began to transform the Franklin County Local Food Council from a  civically-oriented advocacy coalition into a politically-oriented one. In September  of 2012, shortly after the audit was completed, the food council convened to engage  in strategic planning for the coming year. During the strategic planning session the  audit was used to establish background for the work of the council. Ultimately, the  council voted to form three working groups, one of which became the Policy  Working Group, in addition to an Access Working Group and a Supply Chain and  Economic Development Working Group.  Since the affirmation of policy as one of the Council’s three main areas of work,  the Policy Working Group has made great strides in achieving policy-related  actions and objectives. The working group made it an immediate priority to  schedule regular briefings with the Franklin County Commissioners, facilitated by a  local government staff person who was a food council and Policy Working Group  member. Initial meetings with the Commissioners focused on the results of the audit  with particular emphasis on the highest priority identified by the food council: to  work with the Commissioners to pass a comprehensive resolution prioritizing  public health, ecological sustainability and economic development objectives with  regards to the local food system. Fortunately, the president of the Commission had  already bought into local food issues, having supported a community garden initiative  with local refugee populations, as well as a county-funded healthy corner  store pilot program. This commissioner was enthusiastic about the comprehensive  resolution and cooperated with the food council on the creation and passage of the  resolution in time for National Food Day in October of 2013. Although a  non-binding policy, resolution No. 0809-13 explicitly articulates “Franklin  County’s commitment to a strong and resilient local food system” and expresses  support for the mission and vision of the food council while including a commitment  to continue supporting the council’s work in Franklin County (Greer 2013).  Briefings with the Franklin County Commissioners not only yielded the resolution,  but also produced ongoing local government support for the work and  objectives of the council. The president of the Commission committed to encouraging  other local governments to adopt strategies and policies similar to the food  policy audit and resolution, spurring broad-based support for the food council’s  8 The Local Food Policy Audit: Spanning the Civic-Political … 141  values as a policy advocacy coalition while emphasizing the importance of policy in  achieving food system goals. Additionally, the outward expression of support for  local food economies embodied in the resolution led to the Commissioners soliciting  a local food economic development plan for Franklin County. That plan is  now in progress and is being guided by a coalition of partners that includes Franklin  County Planning and Economic Development, Columbus Public Health and a local  food education non-profit. |  |  |
| Clayton et al., 2015, The Role of Partnerships in U.S. Food Policy Council Policy Activities | United States; 2011-2012 | Food policy councils | Investigates the role of partnerships in food systems policy change.  Interviews with representatives and policy experts. | A few FPCs talked about the role of policy expert partners  in helping them to prioritize their policy agenda. For one representative from a local-level,  Midwest-based FPC with a policy partner, this meant engaging partners in FPC meetings or  other activities. For another local-level, Southern FPC without a policy partner, they obtained  input on their policy agenda by sending the agenda to “all the elected officials in [the] community”  and asking for input. | One federal agency representative  described how partnering with government representatives can raise awareness among  policymakers about FPCs and their potential contribution to policy discussions. Partners’ access  to ‘higher level’ stakeholders (e.g., state legislators and agency leaders) and their familiarity  with policy tools (e.g., statements of support) can create new opportunities for FPCs. As this  policy expert explained, ...  In cases where FPCs don’t have policymakers as partners, they may still build reputations as  food policy stakeholders through partnership in government-related groups. As one local-level  FPC interviewee explained, ...  Both our policy expert and FPC interviewees expressed how  partnerships with government leaders and researchers may promote FPC legitimacy through  strategic policy engagement. One policy expert suggested that he could identify when policymakers  are ready to initiate a new policy idea and help FPCs take advantage of that opportunity  by increasing awareness about a problem and a potential solution. Partners can also steer FPCs  away from controversial issues, which may preserve FPCs’ legitimacy in the policy arena, as  one representative from a local-level FPC located in the Midwest explained,  “[This policymaker has] been really great and really protective to make sure that the food policy  council doesn’t become the advocacy arm of that policy and then be forever identified with  this really contentious [issue].”  A few FPC and policy expert respondents talked  about the potential for FPCs to get distracted by the process and lose site of the end goal when  FPCs have had “a little bit of time on the ground” and become involved in issue politics. These  respondents, all associated with state-level FPCs and engaged in state and federal-level policy  work, felt partnerships with policy experts and national organizations helped to refocus FPCs  on a policy end goal. As one policy expert explained,  ‘Policy is a much bigger, bigger lever. So I think I kind of bring that perspective too, we’re doing  all this, we’re bringing these stakeholders together to look at our food system but for the end  goal of making policy change and encouraging our policymakers to do that.”  One representative from a local-level FPC located in the West that did not have policy expert  partners, talked about the need to have “the right infrastructure in place” to do policy work. According to this interviewee, strategic partners who can give FPCs access to stakeholders, provide  strategic policy support, and increase an FPC’s capacity to engage in time consuming policy  processes are an essential part of the infrastructure that FPCs do not have on their own. ...  Several representatives from  local-level FPCs found that partnerships were important for aligning FPC’s policy goals with  stakeholders’ knowledge, needs, and interests; the nature of this influence differed based on  partner type. For example, a representative from a Midwestern FPC talked about partnering  with a non-profit group that inventories vacant land, and credited this group’s land system  with informing the FPC’s approach to urban agriculture policy. For others, relationships with  the community uncovered locally-relevant targets for policy change. One representative from a  Southern FPC explained,  “[Local urban gardeners] identified lots of different policy barriers, zoning, land acquisition,  things like the water, and utility company policies around community gardens. There were  whole lists of different policy issues they identified.” |  |  |
| Fridman & Lenters, 2013, Kitchen as food hub: adaptive food systems governance in the City of Toronto | Toronto, Canada; 2011 | Food policy council | Conceptualize community kitchens as potential food hubs and argue TFPC and TFS mark efforts of inter-scalar urban organising and adaptive food systems governance.  Based on research placements; participatory action research. | The TFPC is a citizen council made  up of 30 citizen stakeholders that span the whole diversity of the food system: from eaters to  farmers, community workers to representatives from the food industry and food business  owners. The TFPC has designated seats for farmers, youth, and city councillors. | The TFPC was founded in 1991 as a sub-committee of the Board of Health to advise the  city on food policy issues as well as advocate for healthy, sustainable, equitable food policies  and programmes throughout the City of Toronto.  The  TFPC’s mandate is to (1) advise and support TPH in the development of food security policies  and programmes; (2) advocate for innovative community food security programmes  across the city; (3) foster dialogue about food issues; and more recently, (4) serve as the  community reference group for the TFS. The TFPC has a full-time coordinator and administrative  support provided by TPH.  The TFS is the newest food-focused entity to be embedded in the municipal government.  The TFS was initiated in 2007, championed by the TFPC members. The TFPC coordinator  at the time supported the TFS development and worked along with other senior city  staff, TFPC members, and food sector leaders in the development of the Food Strategy. The  result of this collaborative effort was a report entitled “Making Food Connections”, released  in 2010 and endorsed by the Board of Health and City Manager. The TFS team was established  to guide the implementation of the strategy. The team is located in the Healthy Communities  directorate of TPH and comprises 4.5 full-time staff, who report to the Director of  the Healthy Communities directorate. The TFPC staff are now a part of this team, all  focused on bringing a food systems perspective to TPH and the City of Toronto. The  TFS’s mandate is to support a health-focused food system and the Food Strategy identifies  six priority areas to guide this work. These are (1) support food-friendly neighbourhoods;  (2) make food a centrepiece of Toronto’s new green economy; (3) eliminate hunger in  Toronto; (4) empower residents with food skills and information; (5) connect city and countryside  through food; and (6) embed food systems thinking in government (Toronto Public  Health 2010). A key approach of the TFS is to create an enabling policy environment for  new food systems initiatives to flourish. This is done by collaborating across TPH and other  city divisions, as well as with the community, on a variety of food systems initiatives (see  Mah and Thang 2013, for a more complete description of the TFS’s approach). Although  the TFPC and TFS have slightly different mandates, they collaborate very closely, work  synergistically, and share office space.  The TFPC with its many  community and business leaders around the table now serves as the community reference  group for the TFS. Food Strategy updates are regularly on the TFPC agenda, and many  TFPC members are involved in Food Strategy initiatives. The TFPC is particularly well placed to accomplish the task of *horizon scanning*, that is, looking for food opportunities  both inside and outside of government. The TFPC continues to develop and inform  policy recommendations that are put forward to both the Board of Health and the City  Council. Since its inception in 2010, the TFS team represents new capacity for food  systems thinking at the municipal level in Toronto.  The TFPC  and TFS, however, are unique in their position inside the municipal government to  advise, enable, and implement policy and programmes that directly shape and influence  the food system.  The TFPC and TFS act as bridging agents between the community, community organisations  and agencies, and city divisions, staff, and policy-makers. Because of the way that  the TFPC and TFS work together with community partners, community-level information  is both transferred and translated into the corridors of municipal government. In this  process, the TFPC and TFS play the important role of translating community concerns  and priorities, so that they can be interpreted in the municipal government environment  and can be communicated for policy purposes (Hajer and Versteeg 2005, Carr 2010).  When information regarding community activities and food-related dilemmas at the community  level are shared at TFPC meetings at City Hall, either by the public or by a  TFPC member, the information is relayed to a diverse TFPC membership, participating  city councillors, and the city staff being the TFPC coordinator and the TFS team.  Between council meetings, the close working relationship of the TFPC and the TFS  allows for ideas from the community to form into initiatives. Community kitchens are  one example where the TFPC and TFS team bring the interests and concern of community  groups into the realm of municipal government, framing community issues as food systems  issues relevant to the city and catalysing staff action. Dialogue is now underway among  policy-makers within the city to facilitate access to kitchens and increase community  kitchen space throughout the city.  The TFS and TFPC shape the policy landscape through informal channels by connecting  community groups to each other, by helping groups navigate the policy environment on  a case-by-case basis (using these experiences as opportunities for both staff and community  members to learn more about the food system and its complex governance networks), and  by supporting pilot projects. Connections are built between communities and the municipality,  self-organisation is promoted and validated, and finally, community leadership is  nurtured. This inter-scalar bridging requires forging deliberate connections between communities,  agencies and organisations, and city staff across departments, jurisdictions, scales, and sectors. It is in this way that the attributes of bridging, self-organisation, and  leadership are expressed. These activities demonstrate possibility and give a platform to  existing motivation, innovation, and creativity within the community. |  |  |
| Giambartolomei et al., 2021, How food policies emerge: The pivotal role of policy entrepreneurs as brokers and bridges of people and ideas | Cork, Ireland & Bergamo, Italy; 2016 | Food policy councils | Policy entrepreneurship and collaborative leadership  Interviews; field observations | The Cork Food Policy Council (CFPC) emerged out of a three-year  community food project led by the Northside Community Health  Initiative (NICHE), that, in an area of marked social disadvantage, had  generated a high level of local engagement. Seeking to build on the  energy and good will of this project led by NICHE, during the summer of  2013 a public meeting to gauge interest in a city-wide ‘sustainable food  project’ was convened by the three key players at this stage: the coordinator  of Cork Healthy Cities, the Director of NICHE who had managed  the community food initiative project, and one of the co-authors, who  was asked to become Chair of the CFPC, given his knowledge of the food  policy field. Given that the public response was so positive, a steering  committee was assembled by invitation, principally on the grounds of  trying to ensure representation from across all sectors of the food system.  The sectors represented by the initial participants of the Steering  Committee are shown in Table 2.  [Table with actors from different groups: academic, public health, civil society, city council, small food businesses  Yet because of the successful  experience of the initial community food initiative on the north-side of  the city, there was legitimacy and credibility of such work in the eyes of  individuals and grassroots organisations. For the Cork Healthy Cities  coordinator, food is regarded as an “ideal tool to bring people together”  (interviewee 9). A particular success has been to achieve ‘buy-in’, not  just from local political representatives, but from key personnel in other  sectors and agencies who would not normally regard health as part of  their brief but who have championed the Healthy Cities agenda.  In the early stages of establishing the CFPC steering committee invitations  to join were extended to two key staff at CCC: the Director of  Environment and Recreation Services and to a member of the Planning  Department. However, their involvement was not directly related to  their institutional mandate with both wanting to participate given their  personal interests and identification with the goals of the CFPC (Interviewees  7 and 8). Indeed, in interviews both stressed the difficulties  of introducing the idea of a FPC to their colleagues, with its multidisciplinary  and cross-sectorial aims rather clashing with the norms of a  bureaucratic municipal organisation which retains a very short-term  and silo approach to local government (Interviewees 2, 3, 4, 8 and 9).  Indeed, cross-departmental and cross-sectorial collaboration still appears  as an inappropriate way of proceeding in local policy-making  (Interviewee 4).  One of the key challenges faced by the CFPC, and noted by outside  observers, has been the difficulty of including greater representation  from the agricultural sector. Although a locally well-known organic  grower participated in the Steering Committee for the first year, she was  unable to sustain her involvement given the demands of the farm.  Finding someone to replace her has proven extremely difficult given the  nature of agricultural production in the Cork region which relies heavily  on dairy and beef. Indeed, with 81 percent of farmed land in Ireland  given over to intensive grass (silage, hay, and pasture) for cattle rearing  and only one percent growing vegetables - the lowest level in Europe –  the mindset of productivism for export has cast a long shadow over Irish  food for a long period (Sage and Kenny, 2017) and, arguably, this has  served to impede the development of short food supply chains in the  city-region.  The Bergamo “Agriculture Roundtable” (Tavolo Agricoltura, hereafter  AR) was established in 2015 as an informal food policy council.  Convened monthly by Bergamo City Council (BCC), from the very  beginning the AR involved representatives and stakeholders from many  sectors of the food system such as: agriculture trade unions (Coldiretti  and Confagricultura), the local association for the safeguard of the local  natural parks (Parco dei Colli), the Botanical Garden of Bergamo, the  CORES research Group7 of the University of Bergamo and several actors  belonging to Sustainable Citizenship (SC), the local solidarity economy  network (see Table 3). The AR was part of a wider strategy that the  Municipality of Bergamo developed within a project called “Nutrire  Bergamo” (literally: “Feeding Bergamo”) which aimed at building a  collaboration among local food actors in order to provide the city with  higher quality food, as well as to valorise the urban and peri-urban  agricultural areas. The AR did not emerge as a formal policy programme,  but rather as a way to embrace the many initiatives around  food and agriculture that were already in place but lacked public support  and institutional recognition.  To conclude this case-study, we suggest that if the lack of participation  by grassroots organisations is a commonly reported experience in  the process of creating an UFS, this seems less evident in the Bergamo  case. On the contrary, what emerges from analysis here is that grassroots  organisations play a vitally important role in shaping the policy debate  around food and sustainability within the territory. Clearly, the presence  of an already established coalition of civil society groups represented a  crucial factor to favour an inclusive food policy initiative (the AR) and to  push further the process of creating an UFS.  However, the creation of ‘alternatives imaginaries’ and cohesive  narratives around which to convene actors from a range of sectors and  backgrounds, almost inevitably raises the question of “who is left out”  from such processes? The two cases show that the partial depoliticization  of the multifaceted role of food at times failed to thoroughly address  issues of unequal power relations, and marginalisation of certain groups.  In this regard, it is important to note that the most prominent and active  individuals and groups engaged in policy entrepreneurship activities do  in fact belong to middle class, educated and privileged backgrounds.  Meanwhile and even before the arrival of the pandemic, growing  numbers of people have been presenting at food banks in Cork as a  consequence of increased economic precarity and the actions of corporate  retailers to dispose of surpluses (ie waste) through charitable donations  (Kenny and Sage, 2019). Sadly, while both the CFPC and the AR  have done much to raise the profile of local food in their two cities, in  neither case have the local authorities extended a commitment to provide  even modest ongoing financial support that would fund, for  example, a coordinator. Instead, both rely heavily on people’s individual  motivation and passion but thereby creating the conditions for disillusionment  and exhaustion, jeopardising the opportunity to involve more  marginalised groups in such practices and governance processes, to  develop just and inclusive UFS. | One of the cornerstones of the CFPC was – and remains - close  collaboration with the Cork Healthy Cities (CHC) initiative, a designation  acquired by CCC in 2012 under the WHO programme. This designation  is not a badge of achievement but a requirement for the local  authority to commit to a process and structure of working towards good  health outcomes. The Healthy Cities approach can be regarded as holistic  requiring ‘One Health’ joined-up thinking that places importance  on inter-agency collaboration and dialogue across sectors. Recognising  the role of food consumption practices in shaping health outcomes as  well as the socio-economic inequalities that prevent some households and communities from accessing healthier dietary options, there was natural synergy to the Healthy Cities initiative working alongside the  emergent CFPC. | Through sharing their knowledge within the community, actors  belonging to both the SC Network and AR as well as the CORES team  have supported the diffusion of a “culture of sustainability and citizenship”,  and raised awareness about being responsible about our daily  choices (Interviewee 17). Nonetheless, the different movements and  associations within the AR as well as within the SC network, mostly have  knowledge associated with their specific area of interest and activity.  The CORES researchers have played a crucial role in reconciling these  different types of ‘place-based narratives’ and thereby demonstrate the  tactic of defining problems and linking issues. This process is not simply  one of harmonizing different local understandings and experiences, but  also to find ways to connect them to a broader vision, including examples  of international ‘best practices’ that might inspire efforts to improve system sustainability. Moreover, the combination of broader environmental  and social concerns together with more locally embedded political  and food activism, fostered the development of a common vision  to build a macro level set of shared aspirations (Stephenson, 2011;  Westley et al., 2013).  The construction of shared aspirations and a common narrative  require linking actors and building networks to further encourage collective  action. The bonds between the various stakeholders have been  reinforced during the monthly meetings of the SC Network since  November 2007, and maintained through the sharing of information and  knowledge facilitated by the CORES group which has proven pivotal in  bridging the different worlds of Bergamo civil society’s activism  alongside external actors. This, as explained by the researchers themselves  when interviewed, can be considered a crucial function entailed  in their action-oriented research: being “translators/interpreters” of the  different narratives of institutions, grassroots movements, private and  third sectors. As highlighted by one of the interviewees (17):  “the great difficulty in bringing these actors together is overcoming ‘particularisms’  – both individual and sector-based – around perspectives and  interests that bring conflict”.  It is worth reminding ourselves  that while grassroots initiatives might be very active at the micro-level,  they do not necessarily see the bigger picture or know what is going on  elsewhere. So academic researchers with their international contacts  and experiences, offer a vital resource to locally based initiatives and,  indeed, help to legitimise these local actions building space for reflexivity  and deliberation within the everyday practices of grassroots  movements. In this regard, one interviewee (13) stressed:  “we always try to create space for reflection, but it is really hard, because  the ‘doing’ aspect takes over the rest. This is what the CORES team helps  us with”.  This facilitation role provided by the CORES team was key in triggering  the interest of the Mayor, who especially values their work in  establishing the relevance of these new practices, which he himself  considers a solid base upon which to start building a local food strategy  (Interviewee 15). |  |
| Godek, 2021, Food sovereignty policies and the quest to democratize food system governance in Nicaragua | Nicaragua; 2007-2018 | Community networks; SSAN (Law of Food and Nutritional  Sovereignty and Security) committees: COMUSSANs | Descriptive analysis of extent of democratization of Nicaragua’s food governance  Document analysis; participant observation; interviews with key informants | But, as García Rocha (2009, p. 17) points out, the CPCs, which are coordinated by political secretaries  of the FSLN, were partisan and their ability to act autonomously  was deeply questioned. This suggests the absence  of mechanisms for true direct democratic practices. Rather,  as Chamorro and Utting (2015), point out, decision-making  became increasingly vertical, rather than horizontal, as the  program’s vision suggested.  In addition to the three factors outlined above, two additional  and important criticisms of the BPA had significant  ramifications for democratic development and practice. The  first again concerns the role of the CPCs in the BPA recruitment,  suspected by CSOs and opposition parties as being  clientelistic, and the second is the issue of government transparency  and the design, implementation, and performance  of the program (Carrión 2018; Chamorro and Utting 2015;  García Rocha 2009; IEEPP 2011; Larracoechea Bohigas  2014; McBain and Leonhard 2007; Spalding 2009). There is  some evidence to support this, with women who identified as  being liberal (opposition) shifting their political allegiance  to FSLN after receiving the BPA (Larracoechea Bohigas  2014). Addressing the issue of clientelism, Orlando Nuñez  argued that this was a possibility in the first few years of the  program, but the allegations of clientelism and favoritism  were not worrying to him; rather, any shift in the political  allegiance on the part of the recipient was because the FSLN  was working to help people and the other political parties  had not organized and served the people:  “To a certain point, it’s normal that Sandinistas will  want to give to other Sandinistas. In the FSLN, there  is sectarianism like in any other political party, but  also the people from other political parties can go to  the countryside, organize their people, protest, exercise  pressure so that their people receive vouchers,  and later form cooperatives. No one is impeding the  liberals [opposition] from doing this. In any case, the  more vouchers we grant, the more food there will be in  the countryside, the more success our political policies  will have, and the more organized [the people] will be.  At the end, these people will return to being Sandinistas.  This is what has happened. In the countryside, the  people were about 30% [Sandinista] and now there is  more than 60% all thanks to Hambre Cero. (Gómez-  Bruera 2016, p. 245)”  The major vehicle for the SSAN Law’s implementation  was a multi-tiered and multi-dimensional institutional framework  that consisted of national, departmental, regional,28  and municipal SSAN committees, as well as an Executive  Secretariat, Sectorial Technical Councils, and an Inter-University  Council. The law detailed the composition of these  institutions, which at all levels included participation by  government representatives, government agencies, and civil  society. The law mandates the expansion and strengthening  of spaces for citizen participation in accordance with the  Law of Citizen Participation (Law 475) passed during the  previous Bolaños administration in 2003.29  For example, in addition  to creating the COMUSSAN, some municipalities also  passed laws against the use of GMOs (e.g., Martínez 2011),  and others wanted to expand membership to include farmers  and local producers, who were not explicitly named as forming  part of multi-scalar councils created by the law (e.g.,  Campo and Meza 2013). These ordinances were mainly  adopted in municipalities where GISSAN and/or other CSOs  and international NGOs were working (e.g., San Ramón,  Nandaime, Pantasma), and they highlight innovations that  resulted directly from the co-production of policy by different  local stakeholders, including local governments and  CSOs. Strategies developed by COMUSSANs emphasized  strong coordination and will to include a broad array of local  state and non-state actors (Campo and Meza 2013; Martínez  2011).32  Regarding citizen participation,  research suggests that the Family, Community, and  Life Cabinets (GFCVs), which replaced the CPCs in 2013  though with a similar mandate and connection to the executive  branch, participated in municipal-level implementation  of the law and the Citizen Power Cabinet was also taking  over the Sectorial Technical Council (Hurtado Diaz 2017,  p. 93). According to the minutes from a workshop on FS and  security in Nicaragua and the implementation of the SSAN  Law held in late 2015, some workshop participants saw the  participation of the GFCVs and Citizen Power Cabinets as  having negative implications for citizen participation in the  institutions created by the law:  “Citizen participation is null if we consider the population  that is not part of the party, since all actions move  under the party structure not allowing the participation  of the civil society in an integral manner (which was  effective in the COMUSSANs that were created until  the departure of Dr. Gonzalez in charge of the Executive  Secretariat). (Hurtado Diaz 2017, p. 98):  In the case of the BPA component of Hambre Cero,  while the program envisioned the creation of local-level  institutions for community policymaking, no evidence was  found to support their formation. There was more success  at establishing cooperatives among the beneficiaries  of the BPA, though experiences were mixed. The clientelistic  means by which beneficiaries were selected and  the decision not to involve NGOs point to limitations on  inclusivity in these spaces. This case illustrates a top-down  approach that suggests the heavy hand of the state compromised  the degree of citizen autonomy and mobilization. | Finally, the third process entailed the  formation of Community Development and Well-Being  Councils, the purpose of which would be to “organize all the  existing organizations and institutions in the locality, consciously  leading, articulating, and managing the economic  processes and policies of the community” (CIPRES 2005,  p. 17).  Furthermore, there is no evidence in  the literature of the formation of Community Development  and Well-Being Councils, envisioned to be the key territorial  mechanism for democratic participation, planning, and  development. It can be inferred that the CPCs were rather  intended to be the primary mechanisms through which local  participants directly participated in decision-making about  their communities. But, as García Rocha (2009, p. 17) points out, the CPCs, which are coordinated by political secretaries  of the FSLN, were partisan and their ability to act autonomously  was deeply questioned. This suggests the absence  of mechanisms for true direct democratic practices. Rather,  as Chamorro and Utting (2015), point out, decision-making  became increasingly vertical, rather than horizontal, as the  program’s vision suggested.  The municipal SSAN committees, known as COMUSSANs,  were envisioned as channels through which municipal  stakeholders could make their voices heard in policymaking  about food security. Their purpose was to carry out intersectoral  decision-making and coordination at the municipal  level, including the formulation and implementation of policies to promote SSAN according to the SSAN Law  (GRUN 2009a, Art. 23 and 25).  As a first step, many municipalities and their relevant  stakeholders formulated and adopted municipal ordinances  recognizing the national law and forming COMUSSANs.  These ordinances created additional municipal-level institutions  for implementing the right to food in their territories.31  The municipal ordinances, in some cases, also had a political  objective of recovering some elements of FS that were lost  in the negotiating of the national law. For example, in addition  to creating the COMUSSAN, some municipalities also  passed laws against the use of GMOs (e.g., Martínez 2011),  and others wanted to expand membership to include farmers  and local producers, who were not explicitly named as forming  part of multi-scalar councils created by the law (e.g.,  Campo and Meza 2013). These ordinances were mainly  adopted in municipalities where GISSAN and/or other CSOs  and international NGOs were working (e.g., San Ramón,  Nandaime, Pantasma), and they highlight innovations that  resulted directly from the co-production of policy by different  local stakeholders, including local governments and  CSOs. Strategies developed by COMUSSANs emphasized  strong coordination and will to include a broad array of local  state and non-state actors (Campo and Meza 2013; Martínez  2011).32  A 2016 study reports that some COMUSSANs were functioning,  but there was an absence of territorial articulation  and planning (Hurtado Diaz 2017).  The findings of Moncayo Miño and Yagüe Blanco’s  (2016) study of COMUSSANs in five municipalities in  northern Nicaragua provide further empirical support. They  found that public sector organizations, and namely local governments,  drove decision-making in the COMUSSANs, and  these processes were notably vertical and influenced by the  national level. Inclusion of other national actors was limited  with a notable absence of national CSOs in decision-making, and some CSOs, like national producer organizations, were  more likely to engage in policy implementation alongside  international NGOs. GFCVs, while not formally part of  the COMUSSAN structure as per the law, played a central  role in the functioning of COMUSSANs. final and critical  insight from this study concerned the issue of citizen  autonomy. Moncayo Miño and Yagüe Blanco (2016) noted  the “inverse relationship between the inclusion of an actor in  the [COMUSSAN] network and the autonomy that the actor  exhibited in their actions… in which there exists dependence  on decisions made at the national level” and further pointed  out how “this feature of the governance model hinders the  participation of actors who do not agree with processes promoted  by the national government” (p. 30).  A key critique of the SSAN Law and the COMUSSANs is  their lack of full implementation, thereby curtailing opportunities  for democratic participation in SSAN. However,  when they have been formed, the goal of creating inclusive  institutions where multiple stakeholders engage in decisionmaking  processes has not been realized. This points to a  serious problem with the law: the lack of political will on the  part of the government to implement it according to its content  (Montano 2009). In the National Human Development  Plan for 2009–2011, dated September 2009 (3 months after  the SSAN Law was passed), the SSAN Law was not mentioned  as part of the national strategy, nor were the COMUSSANs,  but CPCs were. While this changed in the 2012–2016  National Human Development Plan that called for the law’s  implementation (GRUN 2012), the preceding discussion  presents considerable evidence to suggest that it was not  implemented as intended. One explanation for this is that the  government never saw the law or its system as worthwhile,  especially vis-à-vis Hambre Cero. Orlando Nunez called the  SSAN worthless, stating, “Hambre Cero prevailed over it…  [the law] is pure paper. Laws are pure paper. Without a program  [like Hambre Cero], that law is not going anywhere”  (Gómez-Bruera 2016, p. 247). Another explanation has to  do with the legal definition of participation in the SSAN  Law. The law references the Law of Citizen Participation  (Law 475) to legitimize citizen participation, but as previously  mentioned, the Ortega government has completely disregarded  this law and instead fervently pursued its Citizen  Power approach by strengthening the CPC/GFVDs. This has  allowed the government to further limit opposition and more  easily advance its agenda without resistance. With respect  to the SSAN Law, as suggested by the findings of Moncayo  Miño and Yagüe Blanco’s (2016) investigation mentioned  above, GFVDs have served as a vehicle for state cooptation  of the COMUSSANs given they are partisan and an expression  of the national government’s will.  In the case of the BPA component of Hambre Cero,  while the program envisioned the creation of local-level  institutions for community policymaking, no evidence was  found to support their formation. There was more success  at establishing cooperatives among the beneficiaries  of the BPA, though experiences were mixed. The clientelistic  means by which beneficiaries were selected and  the decision not to involve NGOs point to limitations on  inclusivity in these spaces. This case illustrates a top-down  approach that suggests the heavy hand of the state compromised  the degree of citizen autonomy and mobilization. In  the case of the SSAN Law and COMUSSANs, the actions  of local governments and CSOs to develop ordinances and  strategic plans to support the implementation of the SSAN  Law suggested strong local state-society synergies. However,  co-optation of these spaces followed with the government  strategy of strengthening the involvement of CPCs/  GFCVs in the COMUSSANs. This could be seen as a violation  of local autonomy, evoking an example of “competing  sovereignties” (Schiavoni 2015), as well as a direct  attack on NGOs given the conflict between NGOs and the  Ortega government (see, for example, Obuch 2014). NGOs  were seen as a threat to the centralization of power that  the Ortega regime was able to exercise through the CPCs.  In an effort to impose their partisan model of participatory  democracy, the Ortega regime impeded the ability of  potential or known opposition groups to have access to  decision-making spaces while simultaneously disregarding  national laws in favor of presidentially-decreed policies  that suited their interests. |  |  |
| Hasson, 2019, Building London’s Food Democracy: Assessing the Contributions of Urban Agriculture to Local Food Decision-Making | London, UK; 2017-2019 | Food policy council (London Food Board) | Political space framework  Participant observation; informal discussions; grounded theory, content and Foucauldian discourse analysis of organisation websites, reports, and relevant literature. | Anyhow, the Food Board’s membership  was partly renewed in March 2017 and this allowed  for several community food activists to be appointed  by the Mayor among them an activist of the CFGN as  well as two representatives from Sustain (Levidow, 2018,  p. 371). | Another strategy of collaboration with mainstream institutions  is representation within the London Food Board,  this is the equivalent of a Food Policy Council as it puts the Mayor’s Food Strategy into practice, by coordinating  work and leading debate (GLA, 2006, 2018a). According to Hassanein (2003) or Sieveking (2019), Food  Policy Councils offer a concrete example of a deliberate  attempt to develop the practice of food democracy,  but despite the Mayor of London’s aspiration to create  a new food system for the city, it lacks power and resources  to fulfil such an ambition (Morgan & Sonnino,  2010). Moreover, this relatively mainstreamed nature of  London’s food policy is juxtaposed by the complexity of  the city’s local governance structure, consisting of 32 boroughs  and the City of London. These borough councils  are responsible for running most local services in their  areas, and thus there can be significant variation in food  policy priorities and implementation (Coulson & Sonnino,  2019, p. 177).  Anyhow, the Food Board’s membership  was partly renewed in March 2017 and this allowed  for several community food activists to be appointed  by the Mayor among them an activist of the CFGN as  well as two representatives from Sustain (Levidow, 2018,  p. 371). As a result of this participation by community  food activists:  “The Board advocated long-term secure tenancies  for more food-growing, as…basis for infrastructural  investment and organisational commitments in  such spaces…[and] advocated such policies within  London’s strategies for environment, health and  business—more important because these have a  statutory basis. (Levidow, 2018, p. 371)”  Despite this proposition, there was no change made to  the Mayor’s previous policies and the draft London Plan  (responsible for planning urban spaces) made no commitment  to secure food-growing spaces, although it mentioned  aquaponics and vertical growing which are more  relevant to commercial agriculture rather than community  gardening (GLA as cited in Levidow, 2018, p. 372).  This absence of a GLA response around their rationale  for not including any of the proposed policy changes is  problematic when assessing the level of democratisation.  It also indicates that even a quasi-insider role within the  GLA cannot effectively complement public interventions  by the Just Space network in order to facilitate UA, advance  its role in the local food movement and set up  some aspects of food democracy.  Considering all this, it seems that although the democratic  channels such as representation within the institutions  or public consultations exist, the policymaking process  is still centralised. Indeed, despite interesting collaboration  strategies along these channels, civil society  seems to not own the first or final say, and therefore  remain confined to a consultative/implementing role  rather than policy-formulating one. |  |  |
| Henson & Munsey, 2014, Race, culture and practice: segregation and local food in Birmingham, Alabama | Birmingham, Alabama, United States; 2010-2011 | Food policy council | Combination of geographic information systems and Bourdieuan social theory.  Participant observation; in-depth interviews; GIS. | The local food movement is deeply racialized and this became manifest through recent  interventions to create a food policy council. The move to create the council began 5 years  ago with the creation of Birmingham Friends of Food, an organization populated by local  food activists and food and health-related organizations that evolved to include larger  institutional allies. It is run jointly by Reynold’s Urban Farm and Harvest for the Poor, a  gleaning agency. In 2009 and 2010, members of Birmingham Friends of Food received  two large grants—one from a government agency and one from a private foundation. Part  of the money from these grants was used for the creation of a food policy council, and this  initiative has revealed the stark divides along racial lines.  The process began with convening a committee to develop a food charter that would  lay the ideological foundations of the movement and the food policy council. The  committee brought together activists, health professionals, academics, and urban farmers  to frame the message. Of the 20–30 participants (participation and participants varied  significantly from meeting to meeting), only about 5 were people of color, striking in a  city that is 73% Black (U.S. Census Bureau, 2013). Almost all of the participants were  college-educated professionals, and the meetings were held during the day on weekdays.  The language used was technical and focused on how to package food movement  ideology—the importance of local food and farmers, the benefits for health (specifically  childhood obesity)—in ways that would be palatable to middle- and upper-class Whites.  Convening committee participants repeatedly asserted the need for the right messaging  and called for education about the food system during the food charter meetings.  Many of the participants in the creation of the food policy council recognize the  process’s shortcomings regarding diversity, but struggle to develop solutions. This is  apparent in the makeup of the council, chosen in December of 2011. Of the 21 members,  only 5 are people of color, and there is no working-class representation. Notably, the  council lacks any representation from either Black churches or the neighborhood associations,  which constitute the institutional home of the Black community in Birmingham.  Though the council’s stated focus is on food deserts, the habitus represented by the  council is overwhelmingly White and economically privileged. Organizers of the council  understand that the council lacks diversity. As one facilitator commented,  “But I’m really excited to see this diversity around the table, a different type of diversity. . .  First and foremost, we’ve really tried to have diversity at the table, that is, diverse representation  of our community. So, we’ve worked hard to have the business voice at the table. (Swant,  2011, p. 3D)”  The quote is telling because it shows an implied recognition of the lack of class and racial  diversity with the phrase “a different type of diversity,” and that the BJFPC has essentially  a diversity of business, government, and nonprofit professionals. It is a council of elites.  Bourdieu and Passeron (1977) argue that the imposition of a culture is arbitrary and  requires an institution with the authority to impose that culture. Bourdieu further argues  that the institution most responsible for this pedagogic action is the education system.  While none of the institutions involved in creating the food policy council were educational  institutions, educating the public was integral to the mandate of the food policy  council. The government agency and nonprofits involved with the large government grant  collaborated to publish a colorful booklet that diagrammed the local food system and documented different food-oriented organizations working on local food in Birmingham.  However, the booklet lacked effective representation of people of color, had a dramatically  apolitical stance, and focused on health. From this, we can see that the culture being  promoted by the food policy council process was colorblind, technical, and centered on  promoting local food as an antidote to poor health defined in a mainstream, medical–  institutional conception of health. This cultural arbitrary shapes the practices available  within the field of the food policy council.  Mark Hassan (pseudonym) is one of the few Blacks who participated in the food  charter development. He stated that he felt his participation was tokenized; that is, that his  involvement was desirable only because of his status as a person of color (personal  communication). This reveals, not the racially insensitive intent of the group’s organizers,  but the incoherence of Black racial capital (Blackness) in White spaces and in the culture  correlative to that space.  Black and White social agents bring habitus that are forged in Black and White spaces  to the field created by the process of developing a food policy council. Since the  organizers and the majority of participants in the Birmingham Friends of Food are  White, the field created by the food policy council process is also racialized White. For  Blacks to participate in that field, they must make Black racial capital legible in a field in  which Black racial capital is largely incoherent as a capital.  It is clear that the dominant habitus in Birmingham’s alternative food and agriculture  movement is racialized White. The cadre of organizations that get the most funding, the  funders that provide resources, and those with inroads into policy are almost exclusively  dominated by Whites. Black leaders affiliated with various Black nonprofits stated that  local, White-dominated funders only make grants to “their friends” (personal communication).  However, we do not want to give the impression that these exclusionary  practices are purposeful; they are not. They are the result of the more or less automatic  operation of the habitus, produced in White space, and functioning in White fields. |  | Furthermore, Hassan often publicly framed his discussion of the low-income Black  neighborhood that he represents in terms of deficiency. He would frame his community as  poor, high crime, bad education, broken families, and dilapidated neighborhoods. This  stands in contrast to our private conversations outside White fields in which Hassan talked  about White privilege and White supremacy. In this analysis, we do not intend to imply  that Hassan is somehow dishonest or disingenuous—but instead we emphasize that he  implicitly understood what is acceptable in particular fields and culture. This transformation  of Black racial capital into cultural capital appropriate to a field emanating from a  White space is integral for Hassan to build power and influence, and ultimately serves his  community well. However, it is important to point out that, probably nonconsciously,  Whites are determining what is and is not acceptable to say in public. This is a result of  the different racial capitals that Blacks and Whites hold and the ability of Blacks to  transform those capitals into cultural capital legible in White fields.  Since the  organizers and the majority of participants in the Birmingham Friends of Food are  White, the field created by the food policy council process is also racialized White. For  Blacks to participate in that field, they must make Black racial capital legible in a field in  which Black racial capital is largely incoherent as a capital. The Blacks participating in the  food policy council process are therefore forced to wield Blackness in their favor, to use  tokenism to influence the process forming the council. This means that while their  opinions may be perceived as valid, they are not perceived as universally valid, but as  valid only from a Black perspective—that is they “speak for” Blacks. |  |
| Horst, 2017, Food justice and municipal government in the USA | Puget Sound region, Washington, United States; 2010-2015 | Food policy council | Food justice  Document analysis; observations; interviews. | The PSRFPC was established in 2010  as a multi-stakeholder advisory group to the metropolitan planning organization and to the MPO’s members.  While the moment was an important one, the PSRFPC has not sustained its focus over time on  indigenous food systems issues. One challenge is that the tribal member mentioned above stopped  participating in council meetings, and no other council members consistently bring up issues of indigenous  food systems. The PSRFPC has instead focused most of its work on expanding food access in  more conventional ways as discussed earlier, e.g. through direct market farms and farmers’ markets.  In its focus on  being collaborative and welcoming, the PSRFPC invites a diverse array of farmers and food producers to  the table. As a PSRFPC staff member put it, the PSRFPC prioritizes being “neutral” and “participation by  all farmers.” In welcoming all farmers, the PSRFPC is careful to not explicitly criticize industrial or other  environmentally harmful farming practices, nor does it explicitly champion agro-ecological practices.  However, the PSRFPC has not paid consistent or significant attention to labor conditions or the rights  of workers much since the initial meetings. One reason for this gap may be that the union representative  stopped participating in PSRFPC work, and few members have consistently brought up worker issues, even  with the rise of the $15 minimum wage movement nationally and locally, and with farm labor issues gaining  attention by activists and media in western Washington (DeMay, 2014). One challenge for the PSRFPC on  this issue is that labor issues have not historically been the domain of the PSRFPC at large, which, beyond  the PSRFPC itself, tends to focus more on attracting and retaining high-profile industries and high-wage  jobs. The PSRFPC also shies away from issues that are potentially controversial.  The PSRFPC struggles with engaging diverse people on the PSRFPC itself – a common challenge for  food policy councils (McCullagh & Santo, 2015). Some interviewees commented on the Seattle- and  King County-centric orientation of the PSRFPC, which dissuades those living elsewhere from participating.  This is a problem related to equity, particularly now when low-income people of color are  being displaced from the City to distant suburbs. Others commented on the overall dominance by  white professionals. One interviewee, an activist, noted, “There is a crisis of diversity at the table … I  am worried that whatever we come up [with] will be inadequate because of the council makeup.” A  representative from a non-profit organization said, “We have some trouble hearing from the people  who are in the trenches doing the day to day work … What do we do about that?” While the PSRFPC  continues to discuss these concerns, it has not yet succeeded in attracting, engaging and retaining  diverse members. | The PSRFPC promotes  best practices, distributes toolkits, and provides technical assistance to the more than 80 jurisdictions  within the region. The PSRFPC has made equity and justice issues part of its guiding principles.  One of the goals of the PSRFPC is to increase democratic participatory decision-making about food  systems issues. Individual PSRFPC members often talked about this aspect of the PSRFPC. One member, representing a community-based organization, noted the need for more balanced power in decision-  making, “There is significant corporate control of the food system and I want to be part of the  movement for local control. I would like to see democratic participation in the food system.” However,  participants on the PSRFPC feel frustrated by what they view as an inability of the PSRFPC to affect  major power imbalances. One member of the PSRFPC commented,  “We need more equitable systems of power – I think of who is able to influence the Farm Bill, and who is  benefitting ... The cuts to food stamps have huge impacts, but they [those who are impacted] have no or  very limited influence. How do they get themselves in systems of power, get engaged and have influence?” | The PSRFPC is making tentative progress in acknowledging past trauma, specifically around the  destruction of native food systems. In 2013, a PSRFPC member, also a member of the Muckleshoot tribe  and active in indigenous food sovereignty efforts, raised her hand to comment after a presentation  on the local food system. She noted that the presentation focused on conventional commodity foods  (e.g. potatoes, beef, and chicken), and reminded the other PSRFPC members that native communities  harvested over 300 kinds of food pre-white contact, none of which were mentioned in the presentation.  She went on to note that many tribal communities want not more grocery stores or farmers’ markets,  but more access to fishing salmon, hunting duck and elk, and harvesting berries and plants. Reflecting  on how her remark was received, she commented,  “I was impressed with how the statement was received – people were very supportive. Time was made for  me to give a presentation on native food sovereignty at a future meeting. I have felt heard so far – so while  it was stressful to speak up – it was one of my first meetings – it has been rewarding. A hundred years ago  it wouldn’t have been this way. There has been much progress in terms of ethics, practice, how to listen.”  In its focus on  being collaborative and welcoming, the PSRFPC invites a diverse array of farmers and food producers to  the table. As a PSRFPC staff member put it, the PSRFPC prioritizes being “neutral” and “participation by  all farmers.” In welcoming all farmers, the PSRFPC is careful to not explicitly criticize industrial or other  environmentally harmful farming practices, nor does it explicitly champion agro-ecological practices.  As a result, the issue of ecological stewardship remains unaddressed and unresolved. At a meeting in  spring 2015, during a discussion on the future use of King County-owned land (Tall Chief golf course,  purchased in 2013 by King County for $4.5 million for the purpose of turning it into farmland), the  co-chair of the PSRFPC suggested that King County consider requiring ecologically sound food production  practices on the property. In that member’s words, “this is how we transform the system.” However,  other PSRFPC members protested the idea, arguing that it would contribute to the “over-regulation of farmers.” A rancher commented, “The hair on the back of my neck stands when you tell me what to do.  Telling me what to do is naïve.” A staff member of King County admitted that County owned farmland  has not been well-managed in the past, but he likewise resisted “telling farmers what to do.” Another  King County Water and Land Resources Division employee agreed, “We don’t tell farmers what to grow  or how to farm.” As this dialogue illuminates, the PSRFPC faces political and personal challenges to  integrating the principle of agro-ecology into its support for local food producers. |  |
| Koski et al., 2018, Representation in Collaborative Governance: A Case Study of a Food Policy Council | A Western region of the United States (anonymised; referred to as WFPC); 2013-2014 | Food policy council | Representation in collaborative governance arrangements  Coding of council rosters and meetings; interviews | This local council  approached a regional governance and planning body when  members became interested in expanding the council’s service  area. The regional council provided the seed money for  the FPC after recognizing that food policy fits well with the  work of the regional council. To ensure a smooth transition  from the FPC to the WFPC, the regional council created a  transition committee that developed the structure of the  WFPC. The transition committee also gave the WFPC its  current name and came up with 20 seats (initially), to include  two cochairs (one has to be elected), a vice chair, ordinary  members, and some alternates. The WFPC is a committee nested within the regional council. The WFPC has a steering  committee, and both the WFPC and the steering committee  meet once a month (the steering committee is not under  study).  An  initial observation from these data is that the council is  designed to be a roughly equal distribution of nonprofit (29%)  and governmental (30%) sectors, but the reality of attendance  is that city and county officials make up a greater share of all  meetings (22% for nonprofits and 36% for government members),  and have a much stronger average rate of attendance.  Put another way, the  results in Figure 4 suggest a kind of “council within a council”  which equally shares the load as a small group. This small  group of actors’ influence is enhanced by consistently high  attendance at meetings in contrast to other organizations on  the council; however, high attendance does not guarantee high  levels of participation as there are organizations that attend  with greater frequency but participate less in meetings.  Our data characterize the WFPC as a collaborative arrangement  that is structured to represent a diverse array of interests in  the local food system, yet one that displays less substantive  diversity in actual participation in governance. The meeting  minutes offer little explanation on potential causes for group  attendance or participation. We turn to interview data of 13  members of the WFPC to uncover dynamics that contribute to  representation in the WFPC. ... Four dominant themes that shape the participation environment  in the WFPC emerge from our inductive analysis of these interviews:  shared goals, local norms, organizational structure, and  heterogeneity in member capacity.  Shared goals. Representation in a collaborative governance  arrangement is in part a function of members’ desires to participate  but also in the openness of the organization to hear  their contributions. Each of these issues emerges in interviews  with participants as influential in determining participant  contribution to food system governance.  In general, we begin with the expectation that a greater proportion  of shared goals will contribute to greater ability and  desire to contribute. “Shared goals” can be thought of as members  of the council bringing similar goals to the table and/or  agreeing to similar collective goals for the council. On the surface,  the council would seem to exhibit strong goal sharing  across members and for the group itself (e.g., improvement of  the food system). However, this is generally not the case. Given  that the council is designed to be diverse in terms of which  aspects of the food system it intends to address, we would  expect that people coming to the council would have a very  diverse set of goals. Thus, members do not join with the same  level of expertise and shared experience. The onus to encourage  participation and to create a shared set of goals so that participants  have a common sense of purpose falls to the council.  This means that the council needs to show that it has a clear mission, that people understand this mission, and that the council  is the primary vehicle for achieving this mission.  In general, the consensus is that the council is open  (Interviewees 1, 2, 6, 7, 8, and 11) to other groups, while there  is some healthy disagreement (Interviewee 10). This openness  leads to the lack of organization focus we identify in previous  discussion, but for some members, it is the precise reason for  the council’s existence—as an information clearinghouse of  food-related issues for the region (Interviewees 3 and 11). One  member worried that “equity voices [are] not strong—when  they show up they are very loud. Their access issue is a problem.  They are not underrepresented, but they just can’t make it  [to] the meeting” (Interviewee 9). Another member noted that  the culture and structure of the organization are open, but the  fact that the WFPC had reverted to conducting most of its substantive  discussion in the whole meeting (rather than subcommittees)  leads to less actual openness: “It is hard to hear  [members] equally” on “larger issues without involving many  people like 20 or so” (Interviewee 10). Nearly all interviewees  expressed some concern that the council does not or simply  cannot hear all of the issues facing the region’s food system,  though this is largely not thought of as the fault of the WFPC.  This structural openness helps to explain the findings that a  range of issues are represented in the council, even when a  range of individuals might not be (“Topic” findings in Figures  4 and 5).  Nonprofits and special districts, particularly underrepresented  in meeting participation in comparison with their roster presence,  have smaller staff and fewer resources. Private businesses have a  good record of attendance reflecting (in this case) a larger organizational  presence but are less influential in dictating the organizational  agenda of the WFPC. Governments not only have the  capacity to send representatives but also have the capacity to  send alternates. Thus, capacity presents a significant hurdle to  representation in the council. Interviewee 7 presents the classic  trade-off argument succinctly:  “A major issue in the region is that there is a lot of competition for  resources; in particular, there are a lot of nonprofits. [Our group]  tried to get grants through the Council to do more assessment.  Participation can be difficult because there is a cost to participation  and you don’t know what will come out of the process.”  Similarly, Interviewee 2’s statement captures the resource  constraint issue aptly: “Some council members from smaller  offices may face budget constraints and that impacts their  participation [in the WFPC].” | The WFPC’s role in the policy process is to  serve as a linkage between like-minded organizations, to  generate information regarding the food system for policy  makers, and to identify issues on the food policy agenda  of the region. Since its inception, the WFPC has completed  several projects, including a report assessing the  viability of farmers’ markets, a food policy blueprint,  comprehensive planning for urban agriculture, and the  establishment of a database of food-related policies within  the region.  Interview data show a range of statements regarding goals  and goal clarity. Each member envisages multiple priorities  for the food system—some overlapping and others independent.  However, uncertainty regarding the overall purpose of  the council is widespread. A local governmental member  noted, “But in terms of what the council actually does, I’ve  sat there trying to figure that out, to be honest” (Interviewee  4). Another interviewee indicated a catch-as-catch-can mission  that is a function of disparate individual preferences  rather than a coherent strategy: ...  At the same time, it must be noted that the purpose of some  members on the council is to learn about issues that emerge  in the local community. All interviewees valued, to some  extent, the importance of open-ended discourse regarding  food issues, which are understandably diverse. One member  specifically noted that without the council, he or she would  not know what is going on: “I do think they do a good job of  even broadening just a kind of—knowledge of the group.  Which I think is important (Interviewee 11).” Interviewee 2  also noted, “Really diverse group of people who are really  open-minded and that feel comfortable speaking about the  issues they are comfortable with . . . everybody is totally  willing and open to educate themselves.” | While Figure 3 shows that the overall diversity of members  shrinks as representation becomes more substantive,  Figure 4 indicates that the equitability score for significant contributions is much higher than for participation—meaning  that there are many organizations, who generally participate,  but a few members dominate the overall discussion; however,  within the group that offers significant contributions, there is  greater balance across contributors. Put another way, the  results in Figure 4 suggest a kind of “council within a council”  which equally shares the load as a small group.  Local norms. Local cultural norms regarding process are pervasive  through the interview data, suggesting that part of the  council’s success in attendance is a function of an emphasis  on hearing diverse perspectives. Such emphasis on incorporating  diverse perspectives is corroborated by Interviewee 2:  “The council often invites people in the community to present  on some issues to figure out how people can collaborate  and get to know the work of others.” In general, interviewees  laud the council’s sensitivities, but there are some that suggest  that the council is held back from addressing substantive  issues. “Right now, we’re really conflict averse” (Interviewee  1). Interviewee 4 also notes that the process focuses  more on consensus building through niceties than conflict:  “Here the process is that people are nice to each other and  sometimes it drives me absolutely batty. Because it takes a  long time to get anything done.” One member who spent  time in a previous governmental position strongly describes  the process: “I’ve been startled by how much stock we put  into really elaborate, thoroughgoing stakeholder processes to  try to get to a result . . . it’s unprecedented for me, how much  time we spend doing it” (Interviewee 8).  Few entrepreneurs emerge to guide the council beyond  what many see as informative, if benign, discussion. One  interviewee states: “You need a champion” (Interviewee 3).  Another interviewee agrees that “sometimes I think you need  a little bit of the squeaky wheel on the council” (Interviewee  5). The longtime chair is viewed as the principal entrepreneur  of the council by nearly all interviewees, and the representatives  of the regional government body in which the  WFPC is located are viewed as key organizational cogs.  Regarding representation, then, members attend meetings  because of norms of fairness and fairness-favoring candidate  recruitment. However, we see substantive participation distributed across a narrow, vocal range of members who  include WFPC founders and regional governmental representatives  who have particular incentives to ensure organizational  function (regional government representatives are not  formal members of the council). This is evident in the outsized  (in raw and percentage terms) representation of regional  governments and the chair in meeting presence found in  Figure 5, thus corroborating equitability findings in Figure 4. |  |
| Lange et al., 2021, Associations between Food Policy Councils and Policies That Support Healthy Food Access: A National Survey of Community Policy Supports | United States; 2014 | Food policy councils | Document prevalence of FPCs and examine associations between presence FPCs and four types of policyes and practices supporting healthy food access.  Survey |  | Having an FPC was significantly associated with municipal-level policies or practices  to improve access to healthy foods. Among municipalities with FPCs (n = 156), nearly all  (96.9%) reported having at least one policy support for healthy food access, compared to  84.9% of municipalities without FPCs (Table 3). After multivariable adjustment, municipalities  with FPCs had significantly higher odds of having any supports (aOR: 4.1, 95% CI:  1.6, 10.4), supports for food stores (aOR: 3.3, 95% CI: 2.3, 4.7), supports for farmers markets  (aOR: 3.3, 95% CI: 2.0, 5.5), transportation-related supports to increase access to healthy  foods (aOR: 2.4, 95% CI: 1.6, 3.5), and objectives in community planning documents (aOR:  3.4, 95% CI: 2.1, 5.5), compared to municipalities without FPCs. Table 3 contains aORs and  corresponding 95% CIs for the specific supports in each of the above categories. Except  for vans or shuttles as transportation to healthy food retailers, all of the specific policies  and practices examined within each category were also significantly associated with the  presence of FPCs. |  |  |
| MacRae, 1994, So Why Is the City of Toronto Concerned About Food and Agriculture Policy? A Short History of the Toronto Food Policy Council | Toronto, Canada; 1990-1994 | Food policy council | Narrative of personal experiences | Early in TFPC's development a decision  was made (after much difficult  discussion) to assume the form of a  roundtable — a structure deliberately  comprised of people with differing political  views from a variety of food system  sectors. The group assigned to create  the TFPC was itself very diverse,  reflecting the values and opinions of  many sectors (diversity of experience). Collectively, it also contained an  awareness of ecological and community  health promotion principles (experience  of diversity). The TFPC  would ultimately have representatives  from: the farm/rural sector, antipoverty  activists, community organizations,  food systems analysis, the conventional  business sector, the organic business  sector, education, labor, multicultural  organizations, the Toronto Board of  Health, and politicians sitting on  Toronto City Council. | Through the roundtable structure,  TFPC has obtained essential insights  into the agendas of various sectors  which permit potential problems to be  anticipated as proposals are developed.  It was decided that the TFPC should  be administered as a subcommittee of  the city's Board of Health, a standing  committee of City Council. This relationship  provides immediate access to  both the political machinery and preventive  health care knowledge and apparatus  of the city. TFPC's three staff persons are attached to the Department  of Public Health, and their salaries and  other TFPC expenses are paid by City  Council. This arrangement provides a  stable funding base and facilitates the  development of a lateral network of  bureaucratic allies and access to the  bureaucracy's information gathering  systems. It also gives TFPC staff credibility  within the civil service which,  in turn, enhances the TFPC's ability  to gather information and effect  change within the municipal government.  The sectoral structure of the TFPC  has also helped create linkages with  community groups. The TFPC plays a bridging role between community  agencies and the political and bureaucratic  machinery of the city. This role  is primarily catalytic and facilitative.  Members and staff help community  groups organize and coalesce, and provide  strategic advice on how to solicit  support from the municipal government.  We know  from informal communications that our  political advocacy efforts have favorably  affected provincial and federal  government decisions. | The TFPC discussions allow different  sectoral representatives to hear and  understand each other's views. At the  same time, decisions are not made to  reflect a "lowest-common-denominator"  position. |  |
| Mah & Thang, 2013 Cultivating Food Connections: The Toronto Food Strategy and Municipal Deliberation on Food | Toronto, Canada; 2010-2011 | Food policy council | Exploratory case study of the development of the Toronto Food Strategy.  Document analysis; interviews; field notes and observations. |  | While the TFPC has been successful in using its place within TPH to advocate for  change, in essence, identifying and animating food issues, the Food Strategy must function  directly within municipal structures, and must seek buy-in or support from across City Divisions in order to act. It must take on the active process of framing municipal priorities  and engaging various policymakers, planners, and public health practitioners. As such, the  Food Strategy must maximize existing resources and skills, and might be inclined to  pursue projects that are smaller-scale, measurable, and time limited (Morgan and  Sonnino 2010), although the longer the Food Strategy has been in operation, the greater  its capacity to leverage partnerships for more sizable gains. |  |  |
| Mangnus et al., 2019, New pathways for governing food system transformations: a pluralistic practice-based futures approach using visioning, back-casting, and serious gaming | Kyoto, Japan; | Food policy council (game) | Urban food futures  Experimental design with combination of visioning, back-casting and serious-gaming, including ‘Food Policy Council Simulator game’ |  |  | In the first workshop, the budget that the teams received was  100.000 units of a fictional currency similar to yen per round, or  per year in game time. In the first round, the two initiatives set up  by the two FPC-teams were a certification scheme and Oyako  Canteens, a parent-child community kitchen. In both cases the  players spent a significant amount of time discussing the budget,  and actively aimed to not spend their entire budget. In the second  round, the two competing initiatives were an educational initiative  targeting university students, and a scheme that would teach  children about tea farming. Both initiatives were successful over  two years: the tea farming scheme was the winner of round two  because of its level of detail. In the final round of voting, the tea  farm came out victorious as well.  To try and see if more innovative ideas could be stimulated, the  budget was raised for the first round of the second workshop to  1 million yen. The two initiatives that were started in the first  round were a combination of Edible schoolyard, ecolabel and  research center, and a Vegetable Dating Service: connecting  people to the farmers that farm their vegetables, and farmers to  people with rare indigenous seeds. The former won the round  because of the level of detail incorporated into its conception. In  the second round, the budget was said to be limitless. The first  team came up with the KodoMall (KidsMall), a department store  filled with food related activities, running on a virtual currency  that could only be spent by kids. The underlying aim was to  revitalize lost shopping areas while at the same time creating a  youth environment. The other team designed a plan for the Aori  School, an educational tour plan in which youth would take tours  all around Japan and help out and learn from older farmers on  their farms. Although the KodoMall won the head-to-head  matchup, in the final voting round, the Edible schoolyard label  and research center was voted best plan of the day because of its  ambitious goals and high degree of specificity (Appendix 6).  Figure 6 details the outcomes of the postgame surveys conducted  after both workshops. In both workshops most of the participants  indicated that they encountered many new ideas. Examples that  participants gave had mostly to do with the initiatives on the seed  cards that were new to them. The second question that addressed  learning was meant to inquire about the experiential effect of the card-based live role-playing game. The results indicated that all  but one participant had an increased level of understanding what  being a member of an FPC would entail. A majority of  participants in both workshops indicated that they would either  “probably” or “absolutely” join an FPC if given the opportunity.  This indicates a motivating effect of the card-based live roleplaying  game. In each workshop two-thirds of the participants  reported to at least empathize somewhat better with other people’s  roles. The survey results show that many people knew other  participants in both workshops. However, most people knew only  one other person, suggesting that they at most met six new people  in workshop 1 or 10 new people in workshop 2. The survey  outcomes were relatively the most ambiguous for empathy effects.  Some participants reported that they felt uncomfortable  representing other people while they were in the same group, or  uncomfortable representing people when they were not there.  Furthermore, the survey results indicate that participants  increased their understanding of other people’s perspectives more  by actually playing with these people, rather than impersonating  them. |  |
| Mooney et al., 2014, Food policy council movement in North America: A convergence of alternative local agrifood interests? | United States; 2006-2013 | Food policy councils | Convergence among alternative agrifood movements and between alternative and conventional practices.  Literature review; website analysis; email analysis. | The dominant diagnostic framing is adopted from Dahlberg (1994) who defined a food system as a cyclical interaction of five sectors: production, processing, distribution, consumption, and waste. This framing has diffused widely with most FPCs indicating an intention to democratically represent members from these five sectors. However, Harper et al. (2009) note that representation of processing and waste sectors is the least common. Our initial analysis suggests that agriculture, in most but not all states, is also perhaps underrepresented or at least represented by a rather narrow range of agricultural interests, particularly at the more local level. | One of the first concerns we must note is that the very label “Food Policy Council” may be something of a misnomer. Schiff (2008) makes this point by questioning the veracity of both the claims to be primarily about “policy” and/or to be organized as “councils.” ... The former concern is probably more significant insofar as Schiff notes that many FPCS do not really do much “policy” work, but rather tend toward program development. However, Schiff (2008, p.211) makes the interesting observation that FPCs might evolve in both directions:  “These FPCs often begin with a focus on policy development and shift to a greater focus on programs (policy implementation) once recommendations have been submitted. Organizations without this type of mandate usually operate in an opposite manner, focusing initially on program implementation and moving later into policy.”  Sherb, Palmer, Frattaroli, and Pollack’s (2012) recent overview noted that FPCs build partnerships, examine current policies, and support or create programs that address food issues. Their survey of 56 FPCs (Sherb et al, 2012) in the United States found that nearly all (86%) of those FPCs sampled see themselves as engaged in some “policy work.” Burgan and Winne (2012, p.6) echo this in pointing to “the three P’s of community food system work”: projects, partners, and policy. Their view is that FPCs work with partners to implement projects associated with policy goals that include: connecting economic development, food security, agriculture, and environment; supporting locally produced food; reviewing legislation and regulations; making recommendations to government bodies; and gathering/ disseminating information.  One of the more inclusive studies of FPCs was conducted as a joint collaboration between Food First and the CFSC in the form of a survey to which 40 FPCs responded (Harper et al., 2009). ... Despite diversity, key functions were identified: (1) monitoring, evaluating, and influencing policy; (2) fostering more effective communication and cooperation between the five food sectors; (3) serving as a public forum for discussing and addressing issues related to food and advising local government on them; (4) planning and supporting programs and services that address local food needs; (5) fostering research and education on food issues (Harper et al., 2009; see also Clancy et al., 2008).  FPCs reflect interesting tensions along these lines with variable integration of FPCs into official governmental structures. Earlier definitions of FPCs tended to emphasize the relationship with government entities. Some FPCs have been created by legislation or executive order (e.g., ...) and form a united within some administrative jurisdiction. Using CFSC data, Burgan and Winne (2012, p. 12) identify 40 FPCs as tied to government agencies and 111 FPCs that are categorized as independent. Our estimates indicate that especially since 2009, new FPCs are more likely to be relatively independent of the official government. However, few are completely independent of the state but ma operate with government grants, enjoy office space or staff resources in governmental building, or other quasi-official status.  Burgan and Winne (2012, p. 13) note that the strengths of being to tied to the state include: legitimation, public involvement, access to government staff, and coordination across government departments. These advantages can be offset by bureaucratic inefficiency, political infighting, less attention to community desires, and fluctuating support from election to election. Autonomy from the state is argued to have the advantages of more control by activists, less bureaucratic restriction, and access to diverse sources of funding, while this autonomy has the costs of less public accountability, lack of official standing with government officials, and reduced staffing. |  |  |
| Pothukuchi & Kaufman, 1998, Placing the food system on the urban agenda: The role of municipal institutions in food systems planning | United States | Food policy councils | Discussion of the urban food system and how municipal institutions can address it more comprehensively.  No (reflection on) methods | Food policy councils, sanctioned by local governments,  are usually comprised of representatives of  different segments of the food system community, e.g.,  members of farm, hunger prevention, retail food, nutritional  education, and sustainable agriculture organizations  – as well as some government officials. | Almost all try to monitor their  city’s food system and work to get various rips and  tears in that system mended. Some have been more  successful than others in pushing their communities  to address food policy in a more comprehensive way  (Dahlberg, 1994).  Food Policy Councils (FPCs) typically exist outside  government structures, and as the name suggests, function  in an advisory capacity. The Toronto Food Policy  Council is an exception. It currently operates as a  sub-committee of the Board of Health, but is unique  among city sub-committees in that it has a degree of  independence that most do not have. FPCs operate  with minimal resources and often have very little or  no staff support. Despite these challenges, FPCs have  taken on a range of actions in their communities. These  fall under broad categories of research and analysis,  community education, policy advocacy, community development through a food system focus, and food  related service delivery. |  |  |
| Prové et al., 2019, Politics of scale in urban agriculture governance: A transatlantic comparison of food policy councils | Ghent, Belgium & Philadelphia, Pennsylvania, United States; 2013-2015 | Food policy councils | Procedural justice and politics of scale  Comparative case study; interviews; document analysis; participant observation. | Similarly, in Philadelphia the efforts to support professional farmers  or connect them with the actions undertaken in the Philadelphia FPC  are mostly left to other organizations or market dynamics outside the  work of the FPC. An issue often mentioned is that professional farms are  difficult to involve in the FPC because they are located far from the city  center which poses serious logistical challenges.  A second scale (mis)match was found at the grassroots level in terms  of organization of civic engagement in urban agriculture and in the  FPC. Despite the broad range of urban agriculture actors that are present  in Ghent, the only stakeholder groups that show strong civic engagement  in the FPC are the professional farmers and the Working  group urban agriculture who appear to be strategically organized to  exert political pressure. Generally, interviewees identified that citizens  are involved in urban agriculture primarily for personal or social motivations;  a commitment to discuss or engage in politically sensitive  topics related to urban agriculture was largely absent. Furthermore,  urban agriculture stakeholders in Ghent show little or no attention for  the neighborhood level, except in one neighborhood which had acquired  the reputation of a success story in terms of the role of urban  agriculture in community development. As stated above, the Ghent FPC  lacks strategies and resources to include the concerns from the different  networks at different scales. Although the interviews with urban agriculture  stakeholders in Ghent revealed that clear and ample support  from the local government exists, the local government provides it in an  ad hoc manner and on a case by case basis. To advance urban agriculture,  strong collaborative action is required. An overreliance on individualistic  actions and involvement will prove unsuccessful.  Ultimately, the scale mismatch between grassroots concerns and the  Ghent FPC framing of the purposes of urban agriculture (i.e., CO2  neutrality, increase of local food) further decreases the incentives for  civic participation in FPC processes.  Compared to the Ghent FPC, there are more opportunities for citizens  in Philadelphia to translate their concerns in relation to urban  agriculture in the Philadelphia FPC and form a broader political  movement. The strong dynamic in civic engagement can be explained  first by the fact that in Philadelphia, the concerns among communities  and urban agriculture stakeholders - whether government, citizen or  other - in the field are shared to a great extent. Second, numerous  bridging actors or organizations (e.g., lawyers at Public Interest Law  Center of Philadelphia, Neighborhood Gardens Trust) provide important  assistance by collecting and translating citizens' concerns to  higher governance levels. Third, the interviews revealed contrasting  attention to the scale of the neighborhood. In Philadelphia, urban  agriculture stakeholders focus much of their efforts at the neighborhood  scale. Finally, food justice is a well-established vocabulary in  Philadelphia, as in other U.S. cities. Food and food access have become  embedded in a food justice frame that has gradually evolved into a  social movement throughout the USA. However, it should also be noted  that – as demonstrated in previous sections - objectives for urban  agriculture which are not aligned with food justice tend to become  excluded from the urban agriculture governance process (e.g. objectives  of professional farmers and entrepreneurs). This indicates that different  grassroots concerns related to urban agriculture do not find equal access  to the governance process. | The FPCs are policy advisory  organs. Formally, they do not dispose of legislative power nor  many resources to support their work (e.g., land, financial means to  support labor in urban agriculture). In the Philadelphia FPC, financial  support from the government is restricted to covering staff wages. It is  highly dependent on external funding. The Ghent FPC receives a larger,  but limited budget and has no external funding. Informally, both FPCs  differ in the impact they have. In the Philadelphia FPC, most impact is  created by forging partnerships with other departments and institutions  regarding relevant topics. Furthermore, it has also moved toward implementation  of its own policy advice. In Ghent, most impact is created  as a partner in various projects. The Philadelphia FPC has various  working groups (one for each policy objective) and it runs two standing  committees to ensure governance processes run smoothly. The Ghent  FPC has a global strategic plan with different goals and introduced two  thematic working groups in 2016: local food production and social  value of urban agriculture. |  | In both cities, thorough communication about the Ghent FPC's study  and the Land Bank in the Philadelphia FPC suggest that the professional  agriculture question in Ghent and the access to land issue in  Philadelphia are made explicit and tangible. The communication  around specific issues also facilitates organization around them and  increases the likelihood that new initiatives or civic engagement will  also revolve around these topics. This serves to confirm the scale(s) at  which urban agriculture will be developed according to the FPC objectives. |
| Roberts, 2010, Food Policy Encounters of a Third Kind: How the Toronto Food Policy Council Socializes for Sustain-Ability | Toronto, Canada | Food policy council | Personal reflection on development TFPC | In contrast to many citizen advisory boards, which have a competitive selection process to pick one individual at a time, I and the citizen co-chair of the TFPC nominate a slate to ensure diversity in the talents, potential and expertise of the collectivity; the Board of Healthy is free to accept, amend or replace this proposed slate, though in practice my recommended cross-section of talent has always been adopted unanimously.  In the Toronto model for food council membership, the slate as a whole features balance and diversity, but each individual is chosen strictly on the basis of talent.  Finally, the TFPC model of personal membership recruitment on the basis of individual talent contributes to sustain-ability by highligthening the centrality of the public interest, not just market interests, as drivers of sustainability measures. In many areas, particularly in the US, food council members are chosen because they represent a specific stakeholder group, such as supermarket retailers or food banks. I believe this undermines the reputation of the food council as a public purpose group, since the stand-out qualification of members becomes the organization they work for and their obvious purpose on the council is to ensure that the turf of the stakeholder paying their salary is protected. ... The purpose of membership diversity is not to provide representation, but practical knowledge of all sectors to strengthen the value of the council’s holistic perspective. | As many thoughtful and inspired food policy documents illustrate, including ones that I have written, food policy councils which take the policy in the middle of their name literally do burn brightly at first. But then they burn out, for the simple reason that there is no-one in government who has a real job with serious operational responsibility who has the time or mandate to hear, deal with, champion or implement a comprehensive and sustainable food policy.  Policy and its ingredients (a lot more than ideas, we’ll learn, as soon as we deconstruct what policy is) are the very capacity that food councils must work from below to bring into being; the content of policy directives that come down from above will only come later. So we don’t fool ourselves or get ahead of ourselves, it’s crucial for food policy actors to understand that this is how far back the starting line is, despite our fears that when it comes to the planet’s need for sustainability, we are perilously near the finishing line.  Before discussing work external to the TFPC (i.e. over and above maintenance of the TFPC), it’s important to understand that food policy councils don’t do implementation. It is inappropriate for the same agency to do advocacy and implementation because it is crucial in a democracy that people receive a public service without fear or favor of a civil servant’s views on any issues; especially in public health, there needs to be absolute faith in the objectivity of the service provider. ... The TFPC approach is to initiate and support community groups that will carry out negotiation and implementation of such joint community-city initiatives; we are an enabling and empowerment tool but we are not a substitute for communities taking power.  Another single issue, however, proved too much for the TFPC – public sector purchasing by City of Toronto of local food. The TFPC has always favored sustainable food, namely food grown with a minimum of toxic pesticides, Genetically-Engineered seeds, mistreatment of animals, abuse of natural ecosystems, and so on ... That proposal was unanimously defeated by a City Council committee which subsequently adopted a loosely-worded motion favoring more local purchasing. ... But the TFPC has to take its lumps too; it lacked the resources and ability to lead the way based on the primacy of relationship-based initiatives, and so when the matter stood or fell on policy, it fell.  Our defeat on the local sustainable food issue was a flashing red light indicating that the TFPC was playing in a bigger league – where well-funded foundations, links to the mayor’s office, and direct lines to the media counted – without the necessary heft, credibility or social relationships. | It has been my experience that food councils are well-suited to two sustainability-sustaining functions that are rarely supported elsewhere: councils can break free from narrow specialities to champion and embrace cross-disciplinary and cross-departmental collaboration; and, they can engage people as citizens from diverse backgrounds, rather than as representatives of varying special interest groups, and thereby uphold the goal of serving the public interest. Without such institutions mandated to engage governments in multi-departmental collaboration and engage citizens in deliberative democracy, sustainability efforts won’t get out of the starting gate.  Since we have such few resources, we can’t afford to concentrate them on conflict-laden causes since one cause would absorb all our capacity; so we work with people who want to work with us like people who work on beautification or community development.  Food councils, which seek input and consensus from people of many distinct interests and perspectives, are pretty much a guarantee that elegant solutions will be favored over the quick fixes chosen by people in monocultural departments with one-track mindsets who are far less likely to catch recommendations suffering from hardening of the categories. The membership norms of the TFPC provide the human group equivalent of a closed loop in natural systems, and thereby help food councils develop a capacity to think holistically and assess unintended and unwanted effects – what are usually and illogically called side-effects as if they are not predictable consequences of the prescription.  Any sensitive, difficult or complex issues are referred to the alternate month’s TFPC meeting, which is identified as a meeting to sort out troublesome details and usually only attracts TFPC members and close followers. Since meetings compete for time of people who have very little time te spare, every effort is made to ensure that public meetings downplay administrivia and play up unique networking opportunities and presentations that have to be experienced in the flesh.  Over and above the two educational presentations, the meetings are part of a learning organization, at least by the standards of learning theorist Senge. A learning organization is one ‘where people continually expand their capacity to create the results they truly desire,’ he writes, and ‘where new and expansive patterns of thinking are nurtured, where collective aspiration is set free, and where people are continually learning to see the whole together.’ (Senge 1990: 3)  Aside from providing a good time and producing some good results, TFPC meetings create an atmosphere that models processes of dialogue and consensus, establishing a crucial set of social skills as well as an important social-psychological undertone for sustain-ability messages. Consensus, dialogue and buy-in are critical to good energy – a positive and empowering counterpoint to the otherwise foreboding prospects that necessarily inform sustainability efforts in a grim and threatening ear. ... One big job of food council organizers is to concentrate that good energy by going the extra mile to overcome conflict or find a welcoming place of consensus or collaboration. On a broader level of human psychology, the TFPC’s approach to dialogue, consensus and buy-in means modeling and setting an expectation for win-win and we-we solutions that we can continuously improve, rather than win-lose and us-them proposals that end up causing analysis-paralysis. | [TFPC meetings receive guests and external stakeholders] |
| Sadler et al., 2015, Local food networks as catalysts for food policy change to improve health and build the economy | Flint, Michigan, United States; 2011-2012 | Food policy council in the making | Case description of development local food network and food policy council.  Participatory research; field observations; interviews | Specifically, some expressed concern that the concept of equity may be defined differently  among various stakeholders. An FPC board of institutional heads would likely be  challenged on the grounds that residents from certain neighbourhoods were not included,  which could result in alternative (possibly inferior) courses of action not reflective of community  input. Although most active participants agreed with this assessment, some did  lament the absence of institutional involvement. But the slow yet deliberate decisionmaking  process which caused attrition of institutional representatives may have unwittingly  alleviated the concern of institutional takeover. To date, there remains an absence of official  involvement of institutional heads perceived as outsiders – any active participants of the  prospective FPC who do belong to a formal institution are considered to be locals or insiders.  But the group also opposed aligning itself with the closely related LFN – which  recently incorporated as a 501(c)(3) non-profit organisation – citing the lack of advocacy  effectiveness of non-profit organisations. | Research participants expressed a central group of concerns related to the formation of an  FPC, including uncertainties about governmental support for and institutional takeover of  the FPC. Initially, participants indicated that the Detroit (Michigan) FPC’s Policy on Food  Security would be used as a framework for building an equivalent document for Flint. Many  stakeholders expressed a deep concern, however, that as the group became formalised, the  decision-making of the eventual FPC could be turned over to institutional heads, as was the  case in Detroit. Not only could a directorship-style FPC limit the voices of residents, but it  could also alter the popular CBDM framework. |  |  |
| Santo & Moragues-Faus, 2018, Towards a trans-local food governance: Exploring the transformative capacity of food policy assemblages in the US and UK | US and UK; 2016 | Trans-local food policy networks, connecting place-based local food policy groups (covering food policy councils and partnerships)  Also contains insights about LFPGs themselves | Study how local food policy groups connect across different scales; assemblage theory.  Comparison of two networks; participant observation; document analysis; interviews. | Moreover, LFPGs may 347 have varying “memberships” inherently built into their structure, as an informant states:  “I use ‘network’ loosely. We have a governance group, but no official membership” (FPN-349 academic advisor).  Many LFPGs’ memberships consist of an extensive listserv of interested citizens and organizational representatives, a smaller group which attends some meetings, an even smaller group which comes to most meetings and participates in working groups, and sometimes paid staff to organize daily logistics.  Meanwhile, FPN’s creation came as a consequence of the dissolution of another organization, 372 the CFSC (Footnote 3). Since assuming maintenance of CFSC’s listserv and resources in 2012, FPN 373 leadership has been exploring how to expand and amplify the support available for LFPGs. The July 2016 374 advisory committee meeting was the first time advisors had met in person to discuss FPN’s mission and 375 objectives; accordingly, FPN’s long-term role continues evolving.  As Table 1 shows, SFCN’s membership is considerably more structured than FPN’s. Groups must 377 apply to become affiliates, which requires LFPGs to demonstrate they have assembled a cross-sector 378 partnership of food system stakeholders to create and implement an action plan that addresses six 379 specific issue areas. In contrast, like many LFPGs, there is no official FPN “membership.” Instead, FPN 380 considers its primary audience the 284 known LFPGs in the US. However, its membership could also be 381 considered its 1,460 listserv subscribers, or even all those who have attended a presentation or training 382 by FPN staff. The indeterminate permanency and varying levels of affiliation and flexibility within the 383 initiatives under study impact their notions of identity and collective capacities.  For instance, debate exists over what constitutes a diversity of stakeholders. 567 Some view it as a cross-sectoral array of organizational representatives and decision-makers (“grass-568 tops”), whereas others emphasize grassroots community engagement. Some aim to connect 569 local/sustainable food advocates with congruent underlying values, while others urge the inclusion of 570 “conventional” stakeholders to achieve more widespread (though maybe less progressive) change.  Another example comes from discussions about the demographic composition of LFPGs. 671 Observations of SFCN listserv discussions, online resources, and meetings suggested that the priority of 672 engaging community members from diverse classes and races was off the radar. Compared to the US, 673 where every single interviewee brought up the issue of meaningful community engagement, diversity (if 674 mentioned) in UK LFPGs entailed achieving diverse sectoral representation (a requirement to join SFCN). 675 Led by experienced NGOs, SFCN has successfully institutionalized the importance of cross-sectoral 676 partnerships and collaborative development of local food policies, but has not emphasized the larger 677 social and racial justice themes prominent in US narratives. By revealing these trends, the trans-local 678 networks can play a role in fostering more holistic outlooks in LFPGs’ policy and programmatic priorities.  That said, the level of connectivity with actors addressing structural causes of food insecurity 680 and inequity, and the inclusion of diverse voices, varies greatly among LFPGs and remains untapped by 681 the national assemblages. For example, some interviewees pointed out how FPN trainings cater to 682 white, middle class norms, threatening its ability to effectively support LFPG members from different 683 sociocultural and political backgrounds.  These disparities also have a spatial dimension. In the US, informants discussed the low amount 685 of resources, trainings, and technical assistance for LFPGs outside of the East and West Coasts and a few 686 Midwest states, reproducing the wider political economy of the country. Many mentioned how most 687 LFPG work has focused on urban areas, thus rural areas might not see the relevance of creating LFPGs or 688 have as many resources to do so. 689  In the UK, regional geographical differences between the North and South predominated 690 concerns about how SFCN may disproportionately cater its resources. One interviewee discussed how 691 SFCN’s broad membership enticed its political leadership to join: 692  “[Our] Council really likes that other [SFCN flagship cities] are gritty Northern, ex-industrial towns 693 like Liverpool and Newcastle. If the other cities had all been Bristols, Baths, and Brightons, it 694 wouldn’t have been all that excited about the network” (SFCN-LFPG3).  Nevertheless, while SFCN membership includes LFPGs from different regions and political and 697 economic contexts (it intentionally funded flagship cities outside of Southern England), it notably has 698 conferred SFCN awards to almost all11 Southern English cities. Some attendees at SFCN’s 2016 699 conference expressed frustration that Northern LFPGs, who face more barriers to integrated food 700 system reform and are working from different baselines of citizen interest and resources, were not 701 recognized, nor were the “best practices” awarded relevant to their contexts. | Some viewed the loose and fluctuating membership of LFPGs as an impediment to influencing policy change or embedding programmatic sustainability. Without an organized structure and consistent membership, LFPGs may struggle to develop long-term relationships internally and externally or to compose an advocacy voice. Others, however, accentuate the flexibility it provides. For instance, LFPGs can adapt their actions to relevant issues for policymakers, funders, or the public.  Firstly, trans-local network engagement with processes such as Farm Bill or Brexit discussions requires 471 significant time and resources, especially of network staff, and may have limited returns compared to 472 less politically contentious and cumbersome action at local and regional levels. Second, most LFPGs, 473 with their relatively inexperienced and fluctuating memberships, might be unprepared to work on 474 national or international issues that require long-term commitments and organizational and political 475 sophistication. Third, logistical and organizational realities, such as how government-embedded LFPGs 476 cannot lobby on political issues, could also limit LFPGs’ capacity to engage at higher levels. Fourth, the 477 political process is fundamentally defined by scalar separations of political jurisdictions; obstructing it 478 requires convincing politicians to collaborate beyond their purviews in unprecedented ways. Lastly, 479 nearly all interviewees expressed how their advocacy roles were limited due to struggles in identifying a 480 common, shared platform among LFPG members—let alone among other LFPGs—to advocate for at any 481 level. In sum, the transitory nature and dynamic relations within and beyond LFPGs and SFCN/FPN offer 482 both opportunities for transcending traditional spatial imaginaries, as well as challenges in doing so 483 when constrained by political, economic, and temporal realities.  Such contestations were particularly notable in US LFPGs and FPN. Some LFPGs emphasized that 575 their focus was on engaging the community members most impacted by food poverty and lack of access 576 to healthy food—mostly lower-income residents and people of color. This often meant changing the 577 name and nature of the LFPG, shifting from policy to more educational and programmatic initiatives: 578  “FPN and other [LFPGs]...are so wedded to saying it’s food policy when the average person’s… 579 super turned-off by that terminology…when [the former LFPG leadership] were doing ‘food policy 580 listening sessions,’ they had a self-selected group of rich white people. But when I do ‘Food 581 Turnup’ events, I get lots of different people...” (FPN-practitioner advisor).  Debates over the inherent purposes of LFPGs, and the networks supporting them, did not 603 appear to be as concerning to SFCN affiliates. As one interviewee described, most UK LFPGs are called 604 food partnerships because local authorities do not have as many policy powers as their US counterparts. 605 It could also partly be due to the fact that SFCN’s established issue areas and application direct groups 606 towards having similar foci and membership compositions. 607  Ultimately, LFPGs are far from homogeneous. On the one hand, LFPG’s different names, 608 terminologies, and objectives demonstrated the modifications occurring as these new food governance 609 practices and policies travel to places with specific socio-cultural norms and political realities. On the 610 other hand, they raised underlying doubts about whether LFPGs within individual countries and 611 between the UK and US can even be categorized as part of the same phenomenon: 612  “They are very different. [LFPGs] in America and Canada do some of what food partnerships do 613 here… but it’s largely dependent on what and how the structure is set up, what level of funding it 614 has, how it’s integrated into the local authority” (SFCN-practitioner advisor).  On the one hand, LFPGs, and particularly their national umbrella networks, 643 are actively engaging with broader processes of social change. These relational political processes have 644 been particularly notable in how SFCN and FPN have begun to influence the narratives of decision-645 makers and, in an inchoate way, funders. By demonstrating and supporting the spread of LFPGs, they 646 have helped normalize the integration of food into municipal governments’ agendas: 647  “[SFCN’s] creating a food path in municipal politics… there was no mandate, no tradition of 648 talking about food… by making food visible, it allows us to view and value it in different ways. 649 That’s why it’s one of, if not the most, important innovation in the UK sustainable food 650 movement in the last 20 years” (SFCN-academic advisor). | Interviewees emphasized the appeals of participating in LFPGs and SFCN/FPN, including the 493 legitimacy these groups provided to their efforts, reduced feelings of isolation, and capacity to bring 494 diverse voices together to deliberate and identify collective goals.  The momentary and long-term collective identity of these groups is “not neat and tidy as it 534 sounds,” as one interviewee explained (FPN-LFGP2). Numerous debates exist both within LFPGs and the 535 trans-local networks connecting them over how to characterize their fundamental purpose—and hence 536 how to name them and which issues to address—and how to go about resolving these contentions. 537 These issues could be considered destabilizing forces, given their potential to divide members and 538 undermine LFPGs’ potential progress. 539  For many LFPGs, fundamental questions have surfaced around establishing objectives. For 540 instance, interviewees discussed how most LFPGs have pursued low-hanging fruits, “feel-good things” 541 (FPN-LFGP1) like farmers markets and healthy eating initiatives instead of more contentious, but also 542 perhaps more transformative food system issues such as land ownership reform, labor rights, 543 commodity subsidies, dietary recommendations, or Brexit. Practitioners expressed concerns about how 544 collective values and decision-making processes within and between LFPGs have not been determined.  Another coordinator echoed similar thoughts when describing how the LFPG, as a loose association of 555 interested people/organizations, has limited ability to engage with contentious but essential topics: 556  “We’ve been just synching up our work... but how do we actually take a position on something?… 557 We updated the urban ag zoning code, a real success. [But] that’s non-controversial… [When] 558 there was paid sick leave legislation in the city, it was difficult because we count amongst our 559 membership some restaurants that were opposing the bill. With no clear decision-making 560 structure, we weren’t able to make any advance beyond education, information sharing” (FPN-561 LFPG2).  Informants also raised topics that they thought were critical to achieving transformative food 703 system reform but were missing from FPN and SFCN narratives and resources, including food worker 704 labor relations, engagement with businesses, dietary shifts (e.g., away from red meat), and non-food 705 issues underlying food ones. For instance, as one LFPG coordinator described: 706  “Everyone thinks about subsidies, food deserts, food stamps, school lunch but where local 707 governments exert their influence is longer-term, more systems-shifting stuff. Like public finance, 708 structures around bonding and development incentives, land preservation, land acquisition for 709 beginning farmers, maybe even affordable housing… because it’s easier to skip a few meals than 710 a housing or rental payment. That issue-bridging in more substantial ways would be really 711 helpful.” (FPN-LFPG1) |  |
| Schiff, 2008, The Role of Food Policy Councils in Developing Sustainable Food Systems | United States & Canada; ? | Food policy councils | Grounded theory approach, examining emerging role of FPCs in developing sustainable food systems.  Interviews with 13 FPCs | In this manner, FPCs serve to create new networks  among members’ organizations and facilitate the expansion and  implementation of their interests by bringing them into the broader food  system context. This role carries through, outside of facilitating communication  between members’ networks and organizations, to facilitation  and networking among other organizations with which the FPC develops  relationships. Developing connections and capacity for communication  with organizations external to the FPC involves building relationships  with and among businesses, nonprofit organizations, government institutions,  and community groups. Drawing ideas from and facilitating these  networks is a crucial role for FPCs to fill, especially with respect to the  need to include a much wider range of participants than membership  structure might allow for and draw connections between all of the various  aspects of food systems:  “It’s good for us to see ways that we can connect. . . . I think that’s  the best thing we can do and not work in a vacuum. I mean that’s not  what a food system is.” (Interviewee 3B) | Three of the organizations represented in  interviews that used some of this alternate terminology stressed particular  opposition to the qualifier “policy,” stating explicitly that they did not do  policy work and therefore were not “food policy councils.” This opinion  remained despite the fact that these groups demonstrated adherence to a  majority of the aspects defining food policy councils in existent definitions.  As indicated in the above discussion of “food policy council” terminology,  the accuracy of the qualifier “policy” remains debatable. Jill Rubin’s  research (J. Rubins, e-mail communication, September 27, 2005) suggests  that food policy councils do very little policy work and instead focus on  programs or projects from within existing government and private organizations’  policy frameworks.  Several of those interviewed indicated a minimal involvement and  even disinterest in researching, writing, or recommending new policies or  changes to existing policies. Interviewees 1 and 7 discussed engaging primarily in policy work as problematic because of the tendency for this  type of activity to distract from or evade implementation:  “I would say we deal . . . with programs rather than policy. Program,  to me, is how you translate a policy into actionables and  deliverables. How do you budget it? What’s the cost? What’s the  parameter? Who’s responsible? . . . So to have food policy, I’m not  particularly interested in making a contribution to food policy any  more than I am into literary analysis. It’s just an academic area.  I’m interested in developing programs that can be delivered.”  (Interviewee 7)  “I think you could easily choke off the creativity and overly bureaucratize  the issues . . . by trying to just do some policy stuff.” (Interviewee 1)  Conversely, several interviewees described interest in policy issues in  terms of researching, amending, recommending, and writing new foodrelated  policy as an important focus or direction for their work. For those  organizations operating initially under government mandate, their primary  responsibility commonly remains developing policy recommendations  as directed by the sponsoring agency/agencies. Two FPC groups felt  that, during the stages of forming a food policy council, obtaining a government  mandate specifically to work on policy recommendation was of  primary importance in forming the organization:  “In order to make change the big picture has to change. So a group  that can look at policy and will be respected for policy, that is actually  their mandate: for changing policy; seemed like it’s the next  step.” (Interviewee 2A)  These FPCs often begin with a focus on policy development and shift  to a greater focus on programs (policy implementation) once recommendations  have been submitted. Organizations without this type of mandate  usually operate in an opposite manner, focusing initially on program  implementation and moving later into policy. The perception that FPCs  do little actual policy work may be attributed to the fact that most of these  organizations, when in the first few years of existence, do not possess the  resources, stability, networks, and respect to make policy recommendations  to government. Once the resources and reputation are established,  organizations can shift focus:  “Where we are right now is needing to sort of simultaneously do the  actual project work to build the expertise from which policy would  be developed.” (Interviewee 6)  “We will need some things that are more like policy statements, like  with farmers’ markets we need something there. We need a policy  statement on them that brings with it some specific code changes. . . But I think a comprehensive policy will probably come after we’ve  knocked off some specific pieces like that.” (Interviewee 1)  The degree to which FPCs can focus on policy issues also changes in relation  to cycles in government and the amount of support available from  elected officials. A lack of sufficient support can prevent FPCs from  being able to focus on policy change:  “I think it changes every six months . . . and six months from now the  group could have a much stronger relationship with the mayor or  twelve months from now. All of that stuff is constantly dynamic and  constantly changing. But in the two and a half years I’ve been here . . .  I haven’t seen them do much in terms of change policy in the city.”  (Interviewee 3A)  Although as described by this interviewee, some FPCs encounter  difficulties in approaching policy work due to relationships with government,  several organizations still maintained an intention to engage  with food policy. Ten of the FPCs interviewed, including those  expressing some disinterest in policy work, had previously worked  with or intended at some future point to engage in policy work. While  an investigation of the activities and programs of FPCs reveals  minimal involvement in amending and recommending changes to  food-related policies, interviews revealed contrasting interests and  sentiments. Even for those FPCs without a history of this type of  activity, the focus often remained on building credibility and  resources to eventually allow for greater focus in this area. Drawing  from interview responses, it appears that one potential role for FPCs to  fulfill is to raise the awareness of government as to policy, changes to  policy, and implementation mechanisms that can enhance food systems  sustainability. While data collection did not specifically address  the reasoning for the minimal focus on policy work of some FPCs, further  research may be needed in this area.  The majority of interviewees indicated that recognition under a government  ordinance was a critical and deciding factor in the creation of the FPC.  One interviewee suggested that in the initial stages of formation of the FPC,  the organizing group considered the advantages and disadvantages of creating  the organization as a government or a nonprofit entity. This group, as  did the majority of those interviewed, decided that association with government  or being created as a government entity was the preferred method for  operation of an FPC in their area. Several interviewees indicated the reasoning  behind operating as a government organization, with a majority claiming  that this strategy helped gain legitimacy for the organization:  “I think being affiliated with (a government department) has given us  legitimacy within the city.” (Interviewee 11)  “That’s kind of the beauty of the council, is that we’ve been given the  mandate and kind of the authority and the connection to the (government)  to look at these things and to move these ideas up.” (Interviewee 8)  Interviewee 13 expressed a similar sentiment, indicating that the strength  of the nonprofit sector in North America dilutes the effectiveness of FPCs  created solely as nonprofit organizations. When speaking to the nature of  operating as government organizations, several interviewees indicated  that, as a government entity, the ability to translate wider perspectives and  citizen voices and gain authority to implement recommendations through  food policy and planning remained one of the more significant roles of the  government-mandated FPC:  “We need the people that are on the ground, who know the issue  really, really well and have dedicated themselves to just that issue  but might not necessarily have the connection to get what they want  to get done at the (government) level done. So that’s hopefully kind  of the role that we’re trying to fill.” (Interviewee 8).  Whether functioning as a nonprofit, hybrid, or primarily  government-based organization, all of the FPCs interviewed described  one of their primary roles as being a voice for recommending new ideas  or changes to government activities surrounding food policy and planning. | An aspect of, and addition to, the role of food policy councils as voices  for system-wide changes in governance for food policy and planning is the  role that FPCs fulfill as networkers across the spectrum of food system  interests and facilitators in the networking and implementation capacity of  other organizations. Networking is central to the food policy council concept  in that the role of networker allows the FPC to draw connections  between disparate stakeholders in the food system. As articulated by Interviewee  9A, “The goal is to create a system out of which people can communicate  their ideas, talk to each other.” Interviewee 12 indicated that  information exchange and the networks created among members through  the operation of the FPC was one of the most valuable and lasting legacies  of the organization. In this context, FPCs relate to the concept of the learning  organization “where people continually expand their capacity to create  the results they truly desire, where new and expansive patterns of thinking  are nurtured, where collective aspiration is set free, and where people are  continually learning to see the whole together.”3  Bringing together this diversity of viewpoints, expertise, and experience  can create tensions among members such that staff, meeting chairpersons,  or other organizational leaders need to fulfill roles as facilitators to unite  and develop innovative ideas out of sometimes conflicting interests:  “We’ve got a wide range of people who have a different finger on  different pulses of a food system. We’re not all agricultural people, we’re not all food banking people. We’ve got nutritionists, we’ve  got gardeners, we’ve got a wide range of folks. So yeah, I kind of  think facilitating is really a large part of what we need to do.”  (Interviewee 3B)  In this capacity, the FPC as a whole fulfills the role of facilitator and convenor  for inclusiveness of viewpoints from across the food system. This  aspect of facilitation at the most basic level involved the members and  their viewpoints as individuals. At another level, this sort of networking  facilitation relates to the businesses, organizations, institutions, and community  groups that members represent. One role that members may play  is in bringing the interests and activities of the FPC to their own constituencies  for implementation or to help these ideas “come alive.” What the  FPC is asking members is “will you take our ideas to your constituency  and make them come alive?” and then bring their problems to us so we  can wrestle with it in this broader context. The FPC asks members “to  serve here as the dynamic, experienced people who can bring (the FPC)  issues” (Interviewee 7). In this manner, FPCs serve to create new networks  among members’ organizations and facilitate the expansion and  implementation of their interests by bringing them into the broader food  system context.  Through these interests, FPCs also fulfill a role as facilitator for community  consultation and voice for the concerns of community interest groups.  Interviewee 2A discussed this activity as one of the most significant roles of  the FPC in that it is “critical to get into it with other community groups absolutely . . . it’s the very heart of what we’re about.” Working with  community groups and other types of organizations in this respect not  only helps to create and broaden networks but also eases the implementation  of the FPCs planning and policy interests  Internal education, that is, communication, information exchange, and  education for council members, surfaced as one the foremost interests and  concerns of interviewees. The State and Local Food Policy Councils project12  highlights the importance of information-sharing activities in that FPCs:  “Can be an effective and efficient process to provide education and  share information where people involved in all different parts of the food system and government can meet to learn more about what  each does and consider how their actions impact other parts of the  food system.”  Another interviewee felt that the enduring legacy of connections and  shift from conventional to food systems perspectives among participants  was the most significant result and achievement of the group. This interviewee  also indicated food policy council meetings as a crucial point for  educational and communication activities. In the case mentioned above,  food policy council meetings allowed at least one hour of a two-hour  meeting exclusively for information-sharing among participants. Other  interviewees indicated a similar focus. Interviewee 8 identified “educating  ourselves to make our food system more effective and improve it,  move it forward” as one of the most significant accomplishments of the  organization.  The majority of interviewees described the inclusion of presentations  from outside individuals and organizations as an integral component of  food policy council meetings. As one interviewee stated, at FPC meetings  “you don’t just talk business but you bring in somebody with a new idea.”  Presentations usually focus on topics of interest to the food policy council  members. They may be based on topics or speakers suggested by council  members, items related to council priorities, on the proposal of the  presenter, or organized by staff to shift or manage the FPC’s focus and  priorities. Several interviewees described the invitation of certain speakers  as a deliberate move to educate and focus the conversation of the FPC  on particular subjects. For example, Interviewee 7 articulated a growing concern for the linkages between food and energy (oil) which demanded  education of participants on the nature and specifics of the issue.  “Our food policy’s gotta be based on an oil policy or an energy  policy. And so, we’ll be working, girding up, the food policy council  to become a battering ram on that issue and to have the education  and knowledge to move it out.”  Inviting outside speakers represents one avenue for the food policy council  to educate, connect, and communicate with external organizations and  individuals. Interviewee 3A described the mutual benefits gained by  members and outside participants through the invitation to others for  presentations at FPC meetings:  “On occasion . . . we would invite people to the meetings who would  tell us what they’re doing . . . and they would always be surprised  that this meeting even existed, that this group of people existed, and  they would tell us what they’re doing and then we would suggest,  you know “have you contacted this person?” because we’re a wealth  of knowledge.”  Another interviewee described the importance of remaining knowledgeable  about programs and activities occurring in the wider community.  Regular presentations were held during council meetings to achieve this  and to instigate discussion around related program, project, or policy  recommendations.  “We had them make a presentation about that so kind of a “let’s talk  about some really great programs out there that are really kind of  breaking barriers” and that got all the council members talking and  like “oh, we should do this and that and this” and it was really great.”  (Interviewee 8) | Overall, interviews and research revealed the primary functions, demonstrated  and proposed, of food policy councils as coordination points  and, through this networking, as communicators and educators on sustainability  and food systems. Communication and education occurs at two  different levels. The first level involves education of staff, members, and  their constituents. The second level involves coordinating communication  among and education of entire communities including government agencies,  businesses, other organizations, community groups, and individuals.  Communicating ideas, information, and education also occurs through a  variety of outlets. Some of these outlets are based in project activities of  FPCs such as information booths and events, publication of informational  material, and e-communication technologies. Other outlets for this communication  relate to the facilitation of other individuals and groups for the  implementation of project ideas and educational activities that occur as a  component of FPC meetings. Some more nebulous outlets of communication  exist as well in respect to a continuous exchange of knowledge and  information among council participants and their constituent organizations,  governments, businesses, nonprofit organizations, community  groups, and the public.  External education, i.e., educating those who are not members of the  FPC, occurs through the networks and affiliate organizations of members  as well. FPC members gaining new knowledge and broadening networks  through their involvement with the council communicate ideas and  project proposals to their constituent organizations so that the sphere of education and influence grows wider.  Outside of the direct lines of communication between members, staff,  and constituent organizations, FPCs also engage significantly in communicating  principles of sustainable food systems with communities, the  public, and partner organizations through project implementation activities.  One form of this type of communication occurs through release of  informational materials, use of communications technologies, and participation  in celebrations or events, while another form relates to the facilitation  of partners to carry out FPC program and project ideas. Informational  materials published by FPCs include resources such as “buy local”  guides, published reports (such as food system assessment reports), educational  or informational brochures, newsletters, or guidebooks. These  materials act as an important vehicle for public education on sustainability  and food systems, the roles and activities of the food policy council,  and guides for public involvement in food systems work.  All of the FPCs interviewed, with the exception of two very recently  formed, had published and distributed informational brochures and participated  in collaborative efforts to publish food guides or some type of food  system assessment. In contrast, virtually all of the food policy councils  shied away from and avoided contact with external media such as radio,  newspapers, journals, or television. A few interviewees mentioned that  although media exposure was not sought after, requests for interviews  would in most cases not be turned down. Interviewee 6 described the  interest of the media in pursuing interviews with the FPC and others on  one particular issue as useful for indicating public interest and where to  direct the focus of activities:  “We had media asking us for interviews. . . . It was all extremely positive.  It felt great that that was something clearly that (the public)  could get behind and see as a positive thing for the (community) and  there were . . . excellent articles on buying locally and exposing the  issue . . . just days before the interview that we did. . . . So that was  really great and that’s why we’re feeling that the buy local initiative  is very strong right now and there is incredible interest around it so  that’s what we’re focussing on.”  Three different reasons were indicated, however, for general avoidance  of the media. Some simply reported a lack of interest in pursuing these  lines of communication, whereas others cited a potential danger in attracting  possible adverse attention of public officials or organizations that  could threaten the stability of FPC political, public, and resource support.  One interviewee cited an additional reason, describing part of the FPC  role in facilitating other individuals and organizations as allowing these  groups to take the recognition for achievements. In many ways, allowing  politicians and struggling nonprofits to take recognition through the  media and otherwise helps the FPC to gain much-needed and valuable  political capital.  Another method for enabling communication and education of the  public occurs through the use of communications technologies such as  e-mail, electronic newsletters, listservs, and Web sites. Virtually all FPCs maintain  a Web site providing general and sometimes more specific information on membership, ordinances, organizational structure, meetings, accomplishments,  publications, and activities. The types of informational materials  mentioned above may also be published on FPC Web sites. Online  publication of “buy local” or food guides has been attempted by some  FPCs with considerable success, significantly increasing the number of  visitors to Web sites.  Communication and public education also occurs through hosting  or participating in conferences and other events. Hosting events or  conferences can be quite time demanding and needs to be considered  carefully before implementation in terms of logistics, resources, and  volunteer time available. However, this type of activity can serve as  an excellent avenue for attracting public attention, raising awareness,  and education. Another method for participation in public events and  celebrations can occur through holding information stalls or otherwise  participating in events hosted by other organizations. This expands  educational opportunities while avoiding some of the pressures and  responsibilities associated with organizing conferences, forums, and  other affairs. |
| Siddiki et al., 2015, How Policy Rules Shape the Structure and Performance of Collaborative Governance Arrangements | United States; ? (fpcs ‘currently active’) | Food policy councils | Collaborative governance and institutional analysis and development (IAD) framework  Content analysis; interviews | Th ese data show that most FPCs identify the  participants who make up the council. Some council mandates  identify the specifi c types of organizations from which participants  should be selected, while others generally require a certain number of participants from various food sectors. Only a couple of the  council mandates identify credentials that participants should hold.  Across the councils, we found that the councils have variable representation  by the 24 general food system stakeholder groups contained.  Figure 2 represents the diversity of participants groups formally  represented on councils. Councils (represented by gray circles) are  connected to participant groups (represented by black triangles). Th e  number of participant groups represented by councils varies from 1 to  11 groups. Entities coded as belonging to state or local departments  are all government agencies, while some other participant group  categories (e.g., education and food safety) can be composed of some  mix of private, public, or nonprofit entities.  From our analysis, we learned that public FPCs  altogether represent 24 general food stakeholder groups that work  together therein, with one council made up entirely of at-large public  members and one council representing more than 14 food community  stakeholder groups.6 We found that the majority of councils  have members that represent (1) nutrition, health, and hospitals;  (2) urban, local, or sustainable agriculture; and (3) antihunger,  food assistance, food justice, or food access. As would be expected,  these same issue areas are also predominant in councils’ issue foci.  We also found that all but two councils have formal state or local  government representation. As an alternative method for depicting  councils’ stakeholder representation, we used affi liation network  methods to develop the network and to calculate network size,  stakeholder group representation, and dispersion of representation  for each council. Th e results of this analysis show that councils vary  considerably in size and group representation, indicating the importance  of the pool of available community actors in shaping councils.  In general, smaller jurisdictions have fewer interests than larger jurisdictions, but there are important variations within jurisdictions.  Given the overall variation in policy outputs, community actors—at  the city, county, and state levels—are important in shaping FPCs.  We begin to answer this question by pairing measures derived from  our network analysis—mainly, network heterogeneity—with policy  output data. We found that FPCs in category 1 of table 5 (diverse  foci, diverse range of issues addressed in policy outputs) have a larger  number of stakeholder groups represented than those in the other  categories (average heterogeneity score across councils equals 7.46).  FPCs in category 2 (narrow foci, diverse range of issues addressed in  policy outputs) generally follow the same pattern of diverse stakeholder  representation as councils in category 1 (average heterogeneity  score across councils equals 6.68). Category 3 (diverse foci, narrow  range of issues addressed in policy outputs) is mixed in terms  of council size, and councils in this category have less stakeholder  diversity than those in categories 1 and 2 (average heterogeneity score  equals 5.69), but more than councils in category 4, which on average  are the least diverse (average heterogeneity score equals 5.10). In  general, the data indicate that councils with more diverse stakeholder  representation have more diversity in purpose and/or policy issues  addressed. Due to sample size, statistical tests to determine diff erences  across groups would have little power; thus, we qualitatively  investigate these groups further to corroborate our fi ndings.  We found that, in some cases, this collaboration is  facilitated by the mandated presence of certain stakeholders  participating on the councils. That is, council members serve as  liaisons to other stakeholders within the community focused on  issues similar to those of the council. To this end, the formalization  of certain actors’ presence on councils is  representative of the broader array of food  system actors working on a particular issue  within the same jurisdiction. Thus, we get a  better picture of the food policy landscape  and the actors operating within it by  examining who is required to participate in  councils through state and local policies.  First, we fi nd there  is substantial variation in the diversity of  stakeholder groups represented across public  FPCs in the United States—many FPCs have  mandated representation from local and/or  state government, but some do not. Th us, we fi nd variation not only  in whether local governments are part of the FPC, but also variation  within government representation. Additionally, we fi nd variation  in nongovernmental actors’ presence—specifi cally, the health and  food access sectors.  Th is study outlines specifi c mechanisms by which FPCs can be  structured to require ties to specifi c interests through institutional  representation. However, increased inclusion is not always a panacea  for collaboration issues, as we see in our results: the most homogenous  organizations are unable to represent the will of the community  because of a paucity of interests, and the most diverse are  unable because of a cacophony. Part of this  is a function of a broad number of interests,  but our qualitative interviews suggest that  some actors participating in diverse councils  are intentionally representing the groups they  serve at the expense of the food system. | While we probed explicitly about policy  statements and recommendations, we found that interviewees  shared specifi c information about other types of policy outputs as  well (e.g., draft policy language, policy related research) that we  accounted for in our assessment of council outputs.  Overall, table 4 shows that the majority of public FPCs have  produced at least one type of policy output, most often in the  form of policy recommendations. One-third of the councils have  also engaged in policy research, and almost the same number have  produced draft policy language or at least one full policy relating to  food system issues in their respective jurisdictions.  First, our data show that many of  the councils that have narrow or no outputs  lack stable funding from local or state governments. Also, we found that the majority of councils with  narrow outputs are situated at the city level, whereas the majority of  councils with diverse outputs, irrespective of foci scope, are situated  at the state or county level. | In  general, the data indicate that councils with more diverse stakeholder  representation have more diversity in purpose and/or policy issues  addressed.  One prominent fi nding that emerged from our  analysis of interview data is that councils that collaborate with other  food policy stakeholders not represented in the council produce  outputs covering a wide range of issues, despite their purposes being  diverse or narrow. Specifi cally, we found that councils with narrowly  focused outputs do not collaborate with food system stakeholders  outside of those represented within councils, or they collaborate  only with other FPCs. In contrast, councils with diverse outputs  cited a wider range of allies relating to their programmatic and/or  policy work.  Absent other data, the coding of policies produced an inconsistent  result: diverse council foci usually contribute to diverse policy outputs,  but they may not. Our interview data is critical in explaining this  inconsistent fi nding, adding nuance and ultimately strengthening the  link between FPC organizational structure and policy outputs. In a  nutshell: diversity in membership does not always contribute to  diversity in policy output. We found that councils in which members  represent their personal perspectives, independently of or in  conjunction with their organizational perspectives, have more diverse  outputs than councils in which members primarily represent  organizational perspectives. For this latter council type, we found that  some members left their councils because they were not able to  express their personal opinions on the council.  As an example, an interviewee from FPC 14 highlights the decisionmaking  constraints that government offi cials face. Th e interviewee  indicated that the council decision-making process was slowed  because governmental representatives on the FPC served as proxies  for agency heads rather than as informed participants free to make  decisions. Government representatives had to take all issues back to  their administrators for approval or disapproval, which led to a narrower  range of possible policy outputs. Th is same interviewee commented  that organizational representation constrained the types of  activities they could engage in: “A lot of members, because of their  affi liations, cannot engage in advocacy activity, which impacts what  the council can actually do in garnering public support for policy  recommendations.” Th is fi nding regarding individual versus organizational  representation could be explained, in part, by the rationale  that council members are generally vested food community stakeholders  who have both interest and expertise in at least one food  issue. Precluding members’ ability to speak to their areas of expertise  could ultimately thwart a council’s ability to address a wide range  of community issues, even when the council formally has diverse  foci. One interviewee from a “diverse foci, narrow outputs” council  corroborated this sentiment when asked about whether members  represent their individual or organizational interests on the council:  “Th e FPC conversation was never about underlying perspectives or  individual interests—which is why the FPC didn’t succeed as much  as it could have.”  Second, these variations have important implications  for policy outputs and help to make broader claims about the  revealed preferences of how local governments characterize local  food systems. Greater council representation in the broader network  of community food stakeholders is associated with more diversity in  policy outputs foci, and this connectivity can be facilitated by ties  that council members have to other food stakeholders. Th ird, individual  versus organizational representation by council members can  temper whether diversely focused councils end up producing equally  diverse policy outputs. Collaborative governance scholars agree that  who participates in collaborative venues matters (Ansell and Gash  2008). Th is fi nding off ers a more nuanced understanding of how  participation links to outputs therefrom, namely, by qualifying  the eff ect of participation according to the nature of participants’  representation. |  |
| Sieveking, 2019, Food Policy Councils as Loci for Practising Food Democracy? Insights from the Case of Oldenburg, Germany | Oldenburg, Germany; 2016-2018 | Food policy councils | Food democracy dimensions of Hassanein (2008)  Participant observations; interviews; document analysis | After this event, a core group of about ten volunteers  prepared the formation of the FPC and launched the preformation  stage one year later. During this event, four different  committees (see Figure 1) formed and the participants  started planning future activities. The committees  on different food-related topics had the main function of  bringing together pre-existing transformative activities  in Oldenburg and creating a network. These committees  were meant to be open for everyone interested in participating  based on their interests and resources. More formally,  the 15 members of the representative body of the  council (see Figure 1), equally covering civil society, public  administration/politics, and business were formally elected for an initial period of two years shortly before  the official establishment in October 2017.  During this presentation, the initiators  also outlined the need for dialogue between different  stakeholders, e.g., producers and consumers, but also  processors, retailers, and public officials. They also emphasized  the ideal of having all of these groups being  involved in the council, either as a representative or as  an active member in one of the committees. The FPC initiative  strives for collective action towards sustainability  based on a broad group of stakeholders agreeing on a  shared set of values regarding more sustainable food production  and consumption.  The potential for democratising the food system  through FPCs could be assessed by who is represented  in these councils. Considering that most FPCs in Germany,  but also elsewhere, are initiated by civil society and primarily  build on volunteers, FPCs mainly rely on those  who are willing to become part of FPCs. Trying to cover  different societal realms as in the case of the Oldenburg  FPC is just one approach to think about member composition.  Another attempt would be the approach referred to  above (i.e., to have all food system sectors represented),  which is often the case in FPCs initiated through government  policy in the US. | As regards to influencing policymakers and public  officials, the initiative’s members—despite many disappointments  in the beginning—also experienced cases  where they actually had an impact, e.g., the minister of  food and agriculture becoming the FPC initiative’s patron,  the positive approval of a funding request, or the invitation  to be part of a working group on improving the city’s  school catering. A strong motivator to go ahead with the  actual establishment of the council was the strong resonance resonance  manifested in new people joining the group after  the pre-formation event. As one interviewee said: “After  a long period of discussion, also including phases of internal  difficulties manifested in less capacities for preparing  the event, we just needed such a success to go ahead”  (Interview 7). | In its early stages, the FPC initiative offered numerous  opportunities for learning about the food system, simply  by making it possible for individuals to get in touch with  one another. Coming together on this multi-stakeholder  platform, individuals who were ready to collaboratively  strive for a transformation of the current system, encountered  a number of different aspects of the food system.  This diversity of perspectives was also a result of different  ways to be involved, ranging from voluntary engagements  in existing food initiatives, e.g., food sharing, to  formal professional work, e.g., as a restaurant owner or  employee of a retail company. The initiative mostly focused  on the local food system, but dissatisfaction with  the globalised food system often framed their activities.  At the first workshop, for example, a food activist from  South Africa, Zayaan Khan, gave a presentation about  current challenges in the global food system and the  need for local responses.  The committees, as initiated at the pre-formation, particularly  supported self-organized learning in the four different  thematic areas (see Figure 1) chosen by participants.  Despite huge interest in the work of the Education  and Events Committee, it was initially difficult to find  people willing to take on responsibility because of limited  resources. In the following, the committees’ activities  ranged from excursions to farms in the region, harvesting  and processing locally grown food to workshops  in schools. These activities provided learning opportunities  about how to enact alternatives to the predominant  methods of food production and consumption in  daily life.  In its emerging phase, the FPC initiative provided  space for discussion and deliberation in various ways.  Internally, the coordinating group was organized on a  grassroots basis, implying a commitment to consensus  and openness to new members. In practise, decisions  were often prepared by a small group of people (e.g., the  formulation of the initiative’s aims or a concept for an  event), which were then discussed and agreed upon in a  plenary session (Interview 2). This practise implied that  some people were more involved in certain steps than  others; however, they always fed the results back into  the whole group for comments and took decisions collectively  to try to find a consensus. Majority voting was  only rarely used. | All meetings, activities,  and events of the initiative were open to the public  and announced in advance on the homepage.  At the pre-formation event roughly a year later, the  coordinating group defined education and the raising of awareness as central tasks of the initiative. The members  presented examples of food-related events in Oldenburg  where they informed the public about the initiative’s  goals (e.g., a sustainability week at the local university or  a food truck event). On these occasions, the group members  tried to make people think about food issues, for  example with a memory game on the CO2 emissions of  different vegetables (presentation pre-formation event).  Someone always took minutes of the  meetings so people were able to follow what had been  discussed.  Regarding external communication, the group members  approached a huge number of people from different  backgrounds (e.g., the mayor or different parties) and  also participated in public food-related events, such as  panel discussions with representatives from the conventional  farmers’ organization where they were also con-fronted with those who did not share their vision of sustainability.  As time went on, it became increasingly difficult  for the members to monitor their activities (Internal  Meeting 23). Even though the initiative tried to have regular  reports from each committee in the representative  body’s meetings, they did not always have this update  due to a lack of presence or other topics being given  greater priority. For newcomers, it was sometimes not  clear whom they should talk to. Once, for example, a  woman came to the representative’s body meeting to report  on a potentially interesting topic for the initiative  but was then sent directly to the Edible City Committee. |
| Thompson et al., 2020, Assemblage, food justice, and intersectionality in rural Mississippi: the Oktibbeha Food Policy Council | Oktibbeha County, Mississippi, United States; 2016-2019 | Food policy council | Case study of how food policy council can be generated and evolve in historically marginalized rural communities of the South.  Participatory analysis (individual reflections); field notes; document analysis | The OFPC attracted a  diverse group of citizens with a substantial representation of farmers and community members.  As indicated in Table 1, the demographic composition of the OFPC meetings was diverse. At the  meetings in 2017 and 2018, there was an important presence of Blacks farmers and community  members as well as Black MSU faculty and students. During 2017 and 2018, the OFPC meetings  were attended by White women and men, with a great percentage of Black women, Black men  and those who identified as other. In this group, those identified as other were Latinx who were  either MSU faculty or students.  The data from attendance sheets show the diverse and inclusive membership of the OFPC,  which became influential in focusing the discussions on intersectional aspects of the local agrifood  challenges. The fact that many of the MSU scholars (i.e. faculty and students) and community  members (i.e. Black farmers and representatives of institutions working with low income  families) attending to the OFPC were either Blacks or Latinxs had an important impact on introducing  and addressing social justice issues and intersectionality to discuss historic and structural  struggles experienced by people of color in this region of Mississippi.  The genesis of the OFPC was, in part, possible due to existing local institutions already  working on strengthening local food systems and food security in this region. Institutions that  have actively contributed to the OFPC, have been the MFIP, the Stennis Institute of  Government, the Rural Sociology Program at MSU, MSU Extension, the City of Starkville Public Housing, the Winston County Self Help Cooperative (WCSHC), and the Unlimited  Community Agricultural Cooperative (UCAC), among others. Members of the WCSHC and  the UCAC are limited resource Black farmers who have been involved in the OFPC since its  inception. Some members of the UCAC have attended most of the OFPC meetings, significantly  contributing to the mission and work of this assemblage. The involvement of limited  resource Black farmers has represented a substantial foundation for the OFPC, who along  with other representatives from groups and institutions working with low-income families of  color, have been able to acknowledge the opportunities this assemblage represents to tackle  existing local needs and disconnections between food consumers and producers among vulnerable  populations of the community. |  |  |  |
| Zerbian & de Luis Romero, 2021, The role of cities in good governance for food security: lessons from Madrid’s urban food strategy | Madrid, Spain; 2019 | ‘Food policy platform’, which supervises the urban food strategy’s implementation | Good governance  Direct observations; interviews; document analysis | Following the pressure from social movements such as Madrid AgroEcológico (MAE), a  community forum pursuing an agroecological transition, Madrid’s municipality signed the  MUFPP in 2015. The food policy platform (‘La Mesa de Seguimiento del Pacto de Milán’)  was formed shortly afterward, with the challenge of developing a sustainable food system in  environmental and social terms. It is mainly coordinated by the city council, but civil society  and the private sector are also directly involved. Although it is formally recognized, it is still  not institutionalized and officially registered.  Currently, the food policy platform  includes actors from six municipal departments, two municipal bodies, the FAO Office in  Spain and representatives of six social entities.  Civil society  organizations that focused on social exclusion were invited to participate during the strategy  development, and specific initiatives were derived from these interactions.  The general aim of the food policy platform and participatory processes of the strategy was to  reach all concerned actors of the food system and create a space where civil society could co-create  public policies with the municipality. As the need to work with a broader spectrum of agents was  identified through these processes, the membership of the platform was increased. The new configuration  of the food policy platform formed a rich pool of knowledge from the social, private,  and public sectors, including nutrition, commerce, equity, social restoration, and agroecology.  Despite the assertion that the most relevant stakeholders of Madrid’s food system were represented,  interviewees agreed that participation processes were adequate at incorporating the  views of interested civil society groups (‘the usual ones’) but not to include hard-to-engage  groups, the general public, and communities at risk of exclusion. Yet, interviewees acknowledged  that participation is a progressive process and that new members will bring new possibilities of  collaboration and increased representation of different groups. For example, interview results  suggest that due to the inclusion of new members, vulnerable groups were represented through  an organization that works closely with marginalized communities.  Moreover, Madrid’s case demonstrates that urban food governance is not necessarily more  inclusive. Vulnerable and hard-to-engage groups were not included in the food policy platform  or strategy development. Consequently, the promise of urban food governance to democratize  food policy-making remains unfulfilled. For urban food governance tools to develop egalitarian  governance configurations based on alternative governance conceptions (McKeon, 2015), UFSs  and food policy platforms need to redistribute genuine power to marginalized groups and the  interested civil society. Thus, they should be characterized by creating a space where civil society  can voice concerns about food, overcome barriers of public institutional structures, and reconfiguring  power imbalances and exclusion (Moragues-Faus & Morgan, 2015). | In 2017, this platform launched a process for the  development of the UFS. The development of the strategy followed three steps: a diagnosis of  the urban food system of Madrid, the identification of best practices for reference for possible  interventions, and a participatory process to gather the opinion and suggestions of civil society  and Madrid’s citizens.  New responsibilities of the platform after the  development of the UFS are the coordination between local government areas and social actors;  the monitoring and evaluation of the UFS; assessment of the annual report; elaboration of  studies, programmes and projects; development of municipal tools; and the coordination with  other municipal plans and strategies.  Another identified shortcoming was the absence of successful mechanisms to monitor the  actions that result from the platform’s agreements. The document analysis showed that the strategy’s  monitoring process was inefficient. Municipal officials did not deliver updates promptly.  Sometimes, actions were not followed up, leaving it to the interested platform members to  bring up the topics and ask about updates during meetings, without any structured monitoring  framework.  While there was a high level of participation in the development of the strategy, participation  in the implementation of actions was limited. The strategy was perceived as a commitment of the  municipality. Thus, the social sector was not highly involved in its execution, nor did it have the  same power as the municipality in final decisions:  “Our capacity is to propose. The decisions at the end depend on … the executor and the technicians and  the structure of the city council. In fact, I believe that in the platform decisions, decisions, are not taken.  Issues are raised, eh … sometimes discussions are generated and moved to the corresponding spaces, but,  but it’s like a dialogue table, it’s not one, a space, or my perception as a social movement, it’s not a decisive  space.” (S4) | Nevertheless, the inclusion of new food policy platform members challenged network management  mechanisms and questioned how meetings ought to be conducted to ensure deliberation.  While the integration of new members increased areas of action and synergies, it also set  the need for better time management as meetings typically lasted about 1.5–2 hours. During  this time, members took turns to report achievements and advocate for relevant causes. Considering  the increased number of stakeholders, the structure of meetings hindered in-depth debates  and left no knowledge exchange opportunity. | Since its inception, one of this food policy platform’s aims was to publicize the city council’s commitment  to the MUFPP, and related processes, including the strategy. This was reflected  through many initiatives, such as a communication campaign during the launch of the strategy.  Other actions to enhance visibility included an exhaustive communication campaign about the  strategy in 2018 and involvement in advertising events, radio and universities.  The quantity, quality, and access to information had improved since the introduction of the  platform due to the development of a municipal blog that displayed relevant reports, events, and  the city council’s commitments. During data collection, a reform of the blog to include everything related to the strategy and MUFPP in digital format and improve information availability  was suggested. To support the communication strategy of the municipality, civil society  members of the platform also disseminated information about the strategy in their communication  channels and websites. Other efforts to make the initiatives visible included developing  the memory of actions and the annual assembly.  However, while the quantity, quality and access to information had improved, and there was a  transparent attitude from the local government towards civil society, relevant information did not  reach the general public. Thus, the strategy and governance structures were still unknown to  regular citizens. Many interview participants attributed this to disinterest on the part of citizens.  “regarding organized civil society with an interest.… I believe that transparency is great. Regarding other  actors it is less … and well sometimes it’s a little more complicated. How to reach society as a whole? …  well … that’s complex … it’s true that a lot of the public is not worried about whether the actions of the  city council are made through a strategy, if there is no strategy, the organization, that is, what sometimes  interests them is that the services of the city council are of quality and that they have information about  them to be able to use them.” (S1)  Nevertheless, as noted by some interviewees, the lack of interest of the general public did not  mean that visibility efforts should be ignored. Instead, it meant that efforts were not being channelled  effectively and efficiently.  Furthermore, these case study’s findings demonstrate that aligning efforts to increase transparency  is not enough to go beyond the interested civil society and reach the general public. A  lack of awareness among possible beneficiaries may lead to the failure of policies and initiatives  to reach those who need them the most (FAO, 2011). |
