# Supplementary material for: Power to the people? Food democracy initiatives’ contributions to democratic goods
Source: Agric Human Values. 2022 Jul 6;39(4):1477–89. doi: 10.1007/s10460-022-10322-5 (PMC9258474; doi:10.1007/s10460-022-10322-5)
Supplement: Supplementary file 3 — Supplementary file3 (DOCX 13 kb) [file 10460_2022_10322_MOESM3_ESM.docx]

**Online resource 3 Codebook**

**Code book**

**A Inclusiveness**
*The ability of citizens from across different social groups to evenly participate in political decision-making. Includes both formal characteristics of selection mechanisms and the extent to which in practice institutional inducements motivate the engagement of citizens from across groups, so as to avoid marginalization or exclusion.*

***1 Groups*** (i = (largely) included, e = (largely) excluded; e.g. A1ai = citizens general included)
a Citizens general
b Citizens low social-economic status
c Citizens from specific ethnic groups
d Farmers
e Government officials or politicians
f Researchers/ academics/ students
g Food chain actors (other than primary production and consumption) and businesses
h NGOs/ civil society
i Schools and educational institutes
j Healthcare and public health
k Labour unions

***2 Selection mechanisms***
a Participants assigned by government
b Participants selected by non-governmental actors
c No selection
d Sortition

***3 Presence of institutional inducements to engage citizens from across groups***

***Inclusiveness general reflection:*** Code for (meta-)reflections on the importance of inclusiveness.

**B Popular control**
*The degree that participants are afforded increased influence and control within the decision-making process, covering problem definition, option analysis, option selection and implementation.*

***1 Agenda-setting***
a Large influence/ control
b Some influence/ control
c No or hardly any influence/ control

***2 Policy formulation***a Large influence/ control
b Some influence/ control
c No or hardly any influence/ control

***3 Decision-making***
a Large influence/ control
b Some influence/ control
c No or hardly any influence/ control

***4 Implementation***a Large influence/ control
b Some influence/ control
c No or hardly any influence/ control

***5 Evaluation***a Large influence/ control
b Some influence/ control
c No or hardly any influence/ control

***Popular control:*** Code for (meta-)reflections on the importance of popular control.

**C Considered judgment**
*The capacity of citizens to make thoughtful and reflective judgments, including understanding of both the technical details of the issue under consideration and the perspective of other citizens.*

***1 Types of knowledge and information considered*** (i = (largely) included, e = (largely) excluded)
a Scientific knowledge
b Experiential knowledge of food system actors and stakeholders (e.g., farmers, NGOs)
c Cultural or indigenous knowledge
d Views and perspectives of marginalized groups (e.g. those who suffer from food insecurity)
e Policy or political knowledge

***2 Process of deliberation*** (i = (largely) present, e = (largely) absent)
a Deliberation and/or exchange of knowledge taking place
b Presence of (neutral) process facilitators
c Equal opportunities to engage in deliberation/ share perspectives
d Participants engage in deliberation without instruction from or consultation with principal
e Public good prevails over private interests
f General willingness to reflect on own and others’ frames and come to shared understanding
g Use of inclusive language

***Considered judgment general reflection:*** Code for (meta-)reflections on the importance of considered judgment.

**D Transparency**
*The openness of proceedings to both participants and the wider public.*

***1 Toward participants***
a Efforts taken to open proceedings to participants
b No or limited efforts taken to open proceedings to participants

***2 Toward wider public***
a Efforts taken to open proceedings to wider public
b No or limited efforts taken to open proceedings to wider public

***Transparency:*** Code for (meta-)reflections on the importance of transparency.
